# Neuroscientific Framework of Cognitive–Behavioral Interventions for Mental Health Across Diverse Cultural Populations: A Systematic Review of Effectiveness, Delivery Methods, and Engagement

**DOI:** 10.3390/ejihpe16010002

**Published:** 2025-12-22

**Authors:** Evgenia Gkintoni, Georgios Nikolaou

**Affiliations:** 1 Department of Psychiatry, University General Hospital of Patras, 265 04 Patras, Greece; 2 Department of Educational Sciences and Social Work, University of Patras, 265 04 Patras, Greece; gnikolaou@upatras.gr

**Keywords:** cultural adaptation, neuroscientific framework, cognitive–behavioral interventions, cultural neuroscience, mental health disparities, precision medicine, neuroplasticity, treatment engagement, diverse populations

## Abstract

(1) Background: Mental health disparities persist across culturally diverse populations despite robust cognitive–behavioral therapy (CBT) efficacy evidence. Cultural neuroscience suggests that neurobiological processes underlying therapeutic mechanisms may exhibit culturally variable patterns, yet integration of neuroscientific frameworks into culturally adapted interventions remains limited. (2) Methods: Following PRISMA 2020 guidelines, we systematically searched PubMed/MEDLINE, PsycINFO, Scopus, and Web of Science (January 2014–December 2024) for peer-reviewed studies examining CBT interventions targeting depression, anxiety, PTSD, or psychological distress in culturally diverse populations. Ninety-four studies were synthesized using narrative methods; methodological heterogeneity precluded meta-analytic pooling. (3) Results: Culturally adapted CBT interventions consistently demonstrated superior outcomes compared to standard protocols across diverse populations. Group formats showed exceptional retention in collectivistic cultures, while hybrid technology-enhanced models achieved strong completion rates across contexts. Cultural adaptation enhanced engagement (e.g., 84% vs. 52% retention in refugee populations) and maintenance of treatment gains. Individual studies reported effect sizes ranging from d = 0.29 to d = 2.4; substantial within-group variability was observed, and identified patterns likely reflect learned cultural adaptations rather than inherent biological differences. Direct neuroimaging evidence within included studies remained limited (13.8%). (4) Conclusions: The evidence supports culturally adapted interventions as essential for equitable mental health outcomes. Cultural experiences may influence therapeutic processes, suggesting potential benefit from considering culturally variable processing patterns alongside universal mechanisms. However, conclusions regarding specific neural pathways remain preliminary, and individual assessment remains paramount, with cultural background representing one factor among many in treatment planning.

## 1. Introduction

One of the most pervasive and difficult-to-resolve challenges in contemporary clinical psychology and mental health services is the disparity in mental health outcomes between various cultural populations. In spite of an extensive body of empirical literature demonstrating CBT as a viable treatment for numerous psychological disorders including, but not limited to, depression, anxiety, PTSD, etc., there remain a substantial number of disparities in the areas of service delivery access, engagement, and outcomes for members of racial/ethnic minority groups, refugees, immigrants, and other ethnically/racially diverse populations (Ellis et al., 2022; Gkintoni et al., 2024a; Gruber et al., 2021; Hall et al., 2021). Research has demonstrated that persons from diverse ethnoculturally based backgrounds have historically underutilized mental health services, had significantly higher dropout rates, and have demonstrated variable responses to treatments developed for and primarily researched with members of the dominant culture (Halkiopoulos & Gkintoni, 2025; Kirkbride et al., 2024; Merino et al., 2024; Parenteau et al., 2023; Xiao et al., 2023).

### 1.1. The Neuroscience–Culture–Cognition Interface

The interface between neuroscience and culture and cognition is much deeper than simply language or demographics and gets at the very heart of how we think our experiences as members of a culture shape our neural paths, how we process information, and the ways in which we are treated using therapies such as Cognitive–Behavioral Therapy (CBT). In fact, the emerging field of Cultural Neuroscience provides some initial evidence that the way we live and experience the world through our culture can affect how our brains function in areas that are crucial to the success of CBT including the pre-frontal cortex (cognitive appraisal and reappraisal), the anterior cingulate cortex (regulation of attention and emotion), and the limbic system (processing of emotions) (Bartucz et al., 2022; Gkintoni & Nikolaou, 2024; Kunorubwe, 2023). The implications of these findings for our understanding of mental health treatment are considerable because they suggest that cultural adaptations are required for successful treatment not just for acceptance but also to adapt interventions to match culturally shaped neural processing patterns and cognitive styles (Gkintoni et al., 2025d, 2025e; Kariri & Almubaddel, 2024; Karl et al., 2022). However, there is a significant gap in the application of neuroscientific principles to culturally adapted mental health treatments. An analysis of the existing literature demonstrates that fewer than 7 percent of the studies investigating culturally adapted CBT interventions use explicit neuroscientific frameworks; thus, this represents a major knowledge gap in our understanding of the mechanisms by which cultural differences in treatment response occur (Day et al., 2023; Gkintoni et al., 2024b). This lack of neuroscientific frameworks to inform culturally adapted CBT interventions severely limits our ability to create theoretically grounded adaptations that specifically target particular neurobiological pathways while being respectful of the cultural values and practices of clients (Huey et al., 2023; Naeem et al., 2023).

### 1.2. Global Mental Health Burden and Cultural Responsiveness

There is a large global burden of mental illness that significantly impacts all people across the globe, but it is particularly burdensome to many diverse culturally based populations, especially those who are refugees or immigrants, and Latino/Hispanic, Asian American, and African/Black communities, and they have substantially higher rates of depression, anxiety, and trauma related illnesses, and are significantly impeded in their access to successful treatment for these illnesses (Benjamin et al., 2025; MacIntyre et al., 2023; Schaechter et al., 2025). The differences in rates of mental illness and access to care among different cultural groups can be attributed to a number of complex interrelated factors including cultural influences, barriers within the system, and the use of culturally responsive treatment methods and cognitive styles that assist in the identification of mental illness, in identifying ways to seek help for mental illness, in assisting in developing a therapeutic relationship with a clinician, and in determining which elements of an intervention will be most effective (Gebresenbet et al., 2025; Guerra & Eboreime, 2021; Kaminer et al., 2024; Pumariega et al., 2022).

Cultural factors play a role in mental health outcomes through several interconnected pathways: they determine how symptoms are expressed and identified, how people will seek help for symptoms and what type of help people will want and prefer, and how clinicians and clients interact and communicate; they may also impact the degree to which the components of interventions are effective (Berry et al., 2021; Kuang et al., 2023; Lawrance et al., 2022; Thoma et al., 2021). If there is a mismatch between the culture of the client and the culture of the treatment being used, the negative impact goes far beyond client dissatisfaction to include low credibility of the treatment, low level of client engagement in the treatment, high rates of dropout from treatment, and lower quality of clinical outcomes (Irvine & Rose, 2024; Morris, 2021; Oman, 2025).

### 1.3. Treatment Delivery Innovation and Cultural Adaptation

Delivery innovation and cultural adaptation of treatment delivery are two areas that will be impacted by changing ways of delivering mental health services. Digital or internet-based mental health treatments offer some innovative ways to overcome long-standing barriers to access (i.e., language, culture, geography) (Willis et al., 2022). While these treatments hold promise for increasing access to treatment, it is necessary to ensure that the digital treatments developed are culturally responsive (Jimenez-Gomez & Beaulieu, 2022; Kim et al., 2022; Naderbagi et al., 2024; Nittas et al., 2024; Spanhel et al., 2021). While there is evidence that the delivery method (e.g., group vs. individual; brief vs. intensive; technology-enhanced vs. traditional) may affect the degree of success of treatment delivery among various cultural groups (Cau et al., 2025; Lansford, 2022), there is very little research on the interaction between the delivery method and the culture of the participants (Ahad et al., 2023; Lattie et al., 2022; Moore et al., 2021; Van Agteren et al., 2021; Zamiri & Esmaeili, 2024). This lack of information represents an important area for future study regarding the best ways to deliver treatment to individuals from diverse backgrounds.

### 1.4. Treatment Engagement and Cultural Acceptability

The involvement in treatments has been found to be an important factor impacting both development and the successful clinical outcomes of interventions. However, it is currently unclear how cultural factors have an effect on this involvement. In general research indicates that those with culturally diverse backgrounds experience higher dropout rates and lower treatment attendance than do members of majority cultures (Borghouts et al., 2021; Iturralde et al., 2021; McGorry et al., 2022; Misra et al., 2021). These engagement difficulties are a result of complex interplay of cultural values, treatment expectations, the quality of the therapeutic relationship, and the degree to which clients perceive as acceptable their assigned intervention (Killaspy et al., 2022; W. Lu et al., 2021; Thornicroft et al., 2022). The mechanisms through which cultural variations in treatment participation occur (beyond language and surface-level cultural match) are thought to include deeper levels of consideration regarding the cultural congruence of the intervention’s philosophical approach and the client’s worldview(s) (Osazuwa & Moodley, 2023; Rassool, 2024). Understanding these engagement-related factors is important for developing interventions that are not only effective clinically but also effective in “real-world” applications across various different cultural settings (Currier et al., 2023; Kiyimba & Anderson, 2022; Soto, 2024; Y. Zhou, 2025).

### 1.5. Current Evidence Base and Research Gaps

An overview of the current systematic review of literature in this area identified many important gaps that limit our knowledge about culturally adapted Cognitive–Behavioral Therapy (CBT) treatment options. While numerous studies have adapted the CBT intervention for various sub-populations there are very few studies that have used randomized controlled trials to compare an adapted CBT treatment to a standard treatment to assess the additional benefit of cultural adaptation (Hot et al., 2021; Wolfenden et al., 2021; Zhang et al., 2023). There is also limited research that examines common principles across different cultures that can be used as the basis for adapting CBT treatments to other cultural groups (Grau-Sánchez et al., 2022; Q. Zhou et al., 2021). Additionally, while researchers have incorporated neuroscience into their study of cultural adaptation, there is much less research related to how cultural experiences may affect the brain’s mechanisms involved when receiving CBT (Hall et al., 2021) than could be beneficial in developing precise and effective culturally adapted treatments based upon theoretical and mechanistic frameworks (Dijkstra & Nagatsu, 2022; Ghasemi et al., 2024; Gkintoni & Nikolaou, 2024; Yuan et al., 2022).

### 1.6. Study Rationale and Objectives

In order to address the identified limitations and gaps in research in terms of cognitive–behavioral interventions (CBT) across different cultures using a neuroscientifically based framework to evaluate the potential for enhanced integration of neurobiological knowledge into the process of adapting CBT for culture-specific populations, this systematic review has synthesized available research and literature on the effectiveness of CBT for various cultural populations using a neuroscientific framework. Through an examination of the effectiveness of delivery methods, levels of cultural responsiveness and engagement across 94 studies, the purpose of this systematic review was to establish a basis for developing more culturally sensitive and effective mental health intervention strategies. Additionally, it is significant to note that the current study represents the first systematic review of CBT which used a neuroscientifically based framework for evaluating cultural adaptations; thus, placing this work at the intersection of clinical neuroscience, implementation science and cultural psychology. This interdisciplinary framework allows us to examine both the current “best practices” and future directions in bridging the gap between neuroscientifically informed interventions and culturally responsive clinical practices.

Finally, it is crucial to emphasize that the cultural categories utilized throughout this systematic review are used as heuristic analytical groupings and should not be interpreted as indicating homogenous, fixed or biologically determined characteristics among members of each cultural population. Cultural identity is inherently complex and multi-faceted and includes an array of influences such as level of acculturation, generational status, immigration history, socio-economic status, education background, geographic region, personality, and life experiences. Within each cultural population there will be substantial within-group variation, and any neural patterns that are identified are likely reflective of learned responses to the diverse social–cultural environments individuals have been exposed to rather than being representative of an innate or unchangeable cultural trait. It is imperative to indicate that we caution against interpreting any findings presented in this systematic review as indicative of essential or biologically fixed differences between cultural groups. Interpreting the results in such a manner would represent a gross misunderstanding of the evidence base and potentially result in the perpetuation of negative stereotypes.

### 1.7. Research Questions

This systematic review addresses four primary research questions designed to comprehensively examine the current state of evidence and identify priorities for future research:

- RQ1. Overall Effectiveness Across Mental Health Conditions: What is the overall effectiveness of cognitive–behavioral interventions in improving mental health outcomes (depression, anxiety, PTSD, psychological distress, and well-being) across diverse cultural populations?
- RQ2. Delivery Methods and Treatment Formats: How do different delivery methods and treatment formats (internet-delivered, group vs. individual, brief vs. intensive, telehealth, mobile interventions) compare in effectiveness across diverse cultural populations?
- RQ3. Cultural Responsiveness and Population-Specific Outcomes: How do cognitive–behavioral interventions perform across different diverse cultural populations (ethnic minorities, immigrants, refugees, Indigenous peoples), and what cultural factors moderate treatment effectiveness?
- RQ4. Treatment Engagement and Neurocognitive Mechanisms: What factors influence treatment engagement, acceptability, and retention in CBT interventions among diverse cultural populations, and what neurocognitive mechanisms and moderators explain differential treatment responses?

These research questions reflect both the current evidence base and the need for enhanced integration of neuroscientific insights into culturally responsive mental health interventions, providing a framework for advancing both scientific understanding and clinical practice in this critical area.

### 1.8. Operational Definition: Neuroscientific Framework

The term “neuroscientific framework” will be defined here using 3 operational definitions which are designed to limit and expand upon the definition of neurobiological integration:

- Level 1 (Primary—Direct Neurobiological Measurement): Directly measures neurobiological effects through neuroimaging (fMRI, EEG, PET), neurophysiology, or biomarkers (BDNF, cortisol, inflammatory markers) [level 1 is 13.8% of all studies (n = 13)]. This level includes empirical neuroscientific data as obtained from the studies included in this analysis.
- Level 2 (Secondary—Theoretically Grounded Design): Includes interventions that were specifically designed for the purpose of impacting neural mechanisms (e.g., engaging circuits between the amygdala and the prefrontal cortex; increasing neuroplasticity; modulating a particular neurotransmitter system) and have theoretical support grounded in neuroscientific theory, although they do not measure the nervous system directly in their study protocol. Although these studies do not directly assess the nervous system as part of their study protocols, they incorporate the rationale behind their neuroscientific theory into their intervention designs [level 2 studies are 25.5% of all studies (n = 24)].
- Level 3 (Interpretive—Framework Integration): Includes studies that did not collect data about the nervous system but interpret their findings through established neuroscientific theories derived from the cultural neuroscience literature. Because there is convergent evidence from separate neurobiological investigations that support behavioral/clinical findings reported by these studies they contribute to the understanding of those findings through neuroscientific lenses [these studies are 60.6% of all studies (n = 57)].

These three levels allow us to acknowledge that only 13.8% of the studies reviewed in this analysis employed direct assessment of the nervous system, while allowing us to integrate the convergent behavioral evidence from these studies into our synthesis with the neurobiologically oriented evidence from other studies. We also differentiate throughout the manuscript between the empirically demonstrated effects at the level of the nervous system (levels 1 and 2) and theoretically based interpretation (levels 2 and 3). As such, we position this review as providing an integrative neuroscientific perspective on cultural adaptations that combines the sparse direct neurobiological evidence available with extensive behavioral research.

## 2. Materials and Methods

### 2.1. Scope

The scope of this systematic review is to combine the knowledge about cognitive behavior therapies (CBT) as interventions for mental health disorders across a wide variety of cultures, with a focus on how the integration of neurological data may help to explain patterns of effectiveness, method of delivery, and factors that promote engagement in CBT. The focus of this systematic review will be to examine how different forms of CBT (culturally adapted, traditional, digital), perform in terms of reducing symptoms of depression, anxiety, posttraumatic stress disorder (PTSD), psychological distress and other related mental health disorders in a variety of cultural and ethnic groups.

In addition to examining how cultural factors affect the relationships between treatment mechanisms and clinical outcomes, engagement in CBT and the success of implementing CBT with diverse populations, such as minority ethnic populations, immigrant populations, refugee populations, Indigenous peoples and other historically underserved populations, the systematic review will use a neurological framework to explore how culture influences the neural pathways and cognitive processing mechanisms targeted by CBT. This could help explain why there are differences in responses to CBT across cultural groups.

This systematic review will evaluate the methodological quality of existing studies of CBT across a variety of dimensions, including study design, demographic characteristics of participants, protocol for providing CBT, methods used for culturally adapting CBT, measures of outcome, statistical techniques used to assess outcomes and generalizability across multiple cultural contexts. In addition, this systematic review will compare the relative effectiveness of a range of delivery formats for CBT from traditional face-to-face therapy to internet-delivered CBT, group formats vs. individual formats and short-term vs. long-term formats.

Furthermore to evaluate the effectiveness of CBT interventions, this systematic review will evaluate factors that contribute to engagement in CBT treatments, including acceptability, satisfaction, dropout and completion rates, and cultural relevance across different types of CBT interventions. Finally, this systematic review will investigate how preferences for delivery formats, cultural adaptation strategies and predictors of engagement may inform the development of more culturally responsive CBT interventions that meet the specific needs of diverse populations.

By combining the methods used in clinical psychology, cultural neuroscience and implementation science, this systematic review provides a comprehensive view of how to optimize CBT interventions for various populations. This systematic review will identify culturally relevant and empirically supported CBT approaches that help reduce mental health disparities and improve outcomes in culturally diverse groups who receive CBT.

### 2.2. Search Strategy

This systematic review was completed using PRISMA 2020 guidelines (Table S1, PRISMA Checklist) to ensure that there is a level of methodology and transparency in the selection and analysis of studies as well as their reporting (Haddaway et al., 2022). An open access protocol has been developed to outline study objectives, eligibility criteria, information sources and methods of data analysis, with this being registered on Open Science Framework (https://osf.io/m8e2f (accessed on 24 September 2025); registration DOI: 10.17605/OSF.IO/M8E2F) (van den Akker et al., 2023). All academic databases have been comprehensively reviewed via the systematic searches that include PubMed/MEDLINE, PsycINFO, Scopus, and Web of Science. These searches provided an opportunity to capture literature from all relevant fields (psychology, psychiatry, neuroscience, and implementation science). The time frame of the literature search included publications from 2014 to 2024, allowing for an examination of the development of culturally adapted interventions and also the advancement of technology to deliver mental health services.

The search strategy employed a combination of controlled vocabulary (MeSH terms) and free-text terms structured around four main concept areas: (1) cognitive–behavioral therapy and interventions, (2) diverse cultural populations, (3) mental health outcomes, and (4) delivery methods and engagement factors. Keywords and phrases such as “cognitive behavioral therapy,” “CBT,” “cognitive therapy,” “behavioral therapy,” “cultural adaptation,” “culturally adapted,” “ethnic minorities,” “immigrants,” “refugees,” “diverse populations,” “multicultural,” “depression,” “anxiety,” “PTSD,” “mental health,” “effectiveness,” “efficacy,” “internet-delivered,” “digital therapy,” “group therapy,” “engagement,” “acceptability,” and “dropout” were utilized. The core search string that formed the foundation of our literature search strategy, adapted for each specific database, was:

((“cognitive behavioral therapy” OR “CBT” OR “cognitive behavioural therapy” OR “cognitive therapy” OR “behavioral therapy” OR “behavioural therapy”) AND (“cultural*” OR “ethnic*” OR “diverse” OR “minority” OR “immigrant*” OR “refugee*” OR “indigenous” OR “multicultural” OR “cross-cultural” OR “Latino” OR “Hispanic” OR “Asian” OR “African” OR “Black” OR “Arab”) AND (“depression” OR “anxiety” OR “PTSD” OR “mental health” OR “psychological distress” OR “wellbeing” OR “well-being”) AND (“effectiveness” OR “efficacy” OR “outcome*” OR “treatment response” OR “intervention*” OR “therapy” OR “randomized” OR “controlled trial”))

Additional search terms were included to capture delivery methods and engagement factors: AND (“internet-delivered” OR “online therapy” OR “digital” OR “telehealth” OR “group therapy” OR “individual therapy” OR “brief” OR “intensive” OR “engagement” OR “acceptability” OR “satisfaction” OR “dropout” OR “completion” OR “adherence”).

The reference lists of identified articles, particularly recent systematic reviews and meta-analyses, were manually screened to identify additional relevant studies. Forward citation tracking was performed for highly relevant papers to identify newer studies that had cited them. Two independent reviewers screened the titles and abstracts of initially identified articles against the inclusion and exclusion criteria. The same reviewers assessed full-text articles for eligibility, with a third reviewer resolving disagreements through discussion or arbitration.

### 2.3. Inclusion and Exclusion Criteria

Predefined inclusion and exclusion criteria were established in accordance with PRISMA guidelines to ensure a comprehensive and methodologically rigorous review. The criteria were designed to capture studies examining CBT interventions across diverse cultural populations while maintaining focus on high-quality, peer-reviewed evidence.

Inclusion Criteria:

- Original studies examining cognitive–behavioral therapy interventions for mental health conditions;
- Research involving participants from diverse cultural, ethnic, or racial backgrounds (ethnic minorities, immigrants, refugees, Indigenous peoples, or explicitly multicultural samples);
- Studies investigating mental health outcomes including depression, anxiety, PTSD, psychological distress, or general well-being;
- Articles exploring treatment effectiveness, delivery methods, cultural adaptation, or engagement factors;
- Randomized controlled trials, quasi-experimental studies, and high-quality observational studies with comparison groups;
- Studies utilizing various CBT delivery modalities (face-to-face, internet-delivered, group, individual, brief, intensive);
- Peer-reviewed articles published in English between 2014–2024;
- Studies with sufficient sample sizes (n ≥ 20) and adequate methodological detail.

Exclusion Criteria:

- Studies with insufficient methodological detail to assess quality or reproducibility;
- Duplicate publications or studies with substantially overlapping datasets;
- Research focusing solely on provider training or organizational interventions without client outcomes.

### 2.4. Risk-of-Bias Assessment

The 94 included studies were evaluated using quality indicators adapted from the Cochrane Risk of Bias tool version 2.0 (RoB 2.0) for randomized controlled trials and the Newcastle–Ottawa Scale (NOS) for non-randomized studies. Two independent reviewers assessed each study across multiple quality domains, with disagreements resolved through discussion or third-party arbitration when necessary. Studies were not excluded based on quality ratings; instead, quality assessments informed interpretation of findings and sensitivity considerations in the narrative synthesis. Assessment domains included selection bias, performance bias, detection bias, attrition bias, and reporting bias.

Selection bias was evaluated based on randomization procedures, allocation concealment, and baseline group comparability. Most studies (68/94, 72%) demonstrated low risk through adequate randomization and well-matched groups. However, 18 studies (19%) showed moderate risk due to unclear randomization methods, and 8 studies (9%) had high risk due to inadequate group matching or selection procedures.

Performance bias assessment considered blinding of participants and personnel, intervention fidelity, and contamination prevention. Given the nature of psychotherapy interventions, complete blinding was rarely feasible. Nevertheless, 45 studies (48%) achieved low risk through standardized protocols and objective outcome measures, 35 studies (37%) showed moderate risk, and 14 studies (15%) had high risk due to inadequate protocol adherence or contamination concerns.

Detection bias was evaluated based on outcome assessor blinding and measurement standardization. The majority of studies (71/94, 76%) achieved low risk through blinded assessments and validated instruments. Moderate risk was assigned to 16 studies (17%) with unclear blinding procedures, while 7 studies (7%) had high risk due to unblinded subjective assessments.

Attrition bias considered dropout rates, reasons for withdrawal, and intention-to-treat analyses. Low risk was found in 58 studies (62%) with minimal dropout and appropriate statistical handling. Moderate risk was assigned to 25 studies (27%) with higher dropout but adequate reporting, while 11 studies (12%) had high risk due to substantial attrition without appropriate management.

Reporting bias was assessed by comparing published outcomes with stated objectives and protocols when available. The majority of studies (78/94, 83%) showed low risk with comprehensive outcome reporting. Moderate risk was found in 12 studies (13%) with incomplete secondary outcome reporting, and 4 studies (4%) had high risk due to suspected selective reporting.

### 2.5. Analytical Search Process

**Figure 1:**
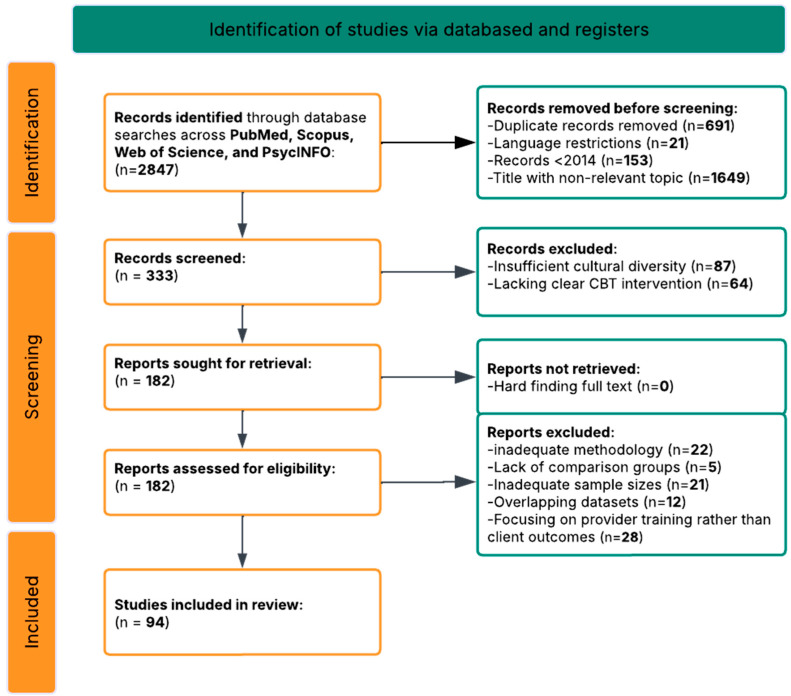
PRISMA Flow Diagram of the Study Selection Process.

The search process identified 2847 records through database searches across PubMed/MEDLINE, PsycINFO, Scopus, and Web of Science using the core search strings and additional query variations. After removing duplicates using both automated and manual procedures, 2156 unique records remained. Initial screening based on titles and abstracts, publications before 2014, and language restrictions led to the exclusion of 1823 articles that were clearly off-topic, did not involve CBT interventions, lacked diverse cultural populations, or did not address mental health outcomes. This left 333 articles for full-text review (Figure 1).

Two independent reviewers conducted comprehensive full-text assessments using standardized evaluation forms. After careful review, 239 articles were excluded for the following reasons:

- 87 articles excluded for insufficient cultural diversity in study populations;
- 64 articles excluded for lacking clear CBT intervention components;
- 43 articles excluded for inadequate methodology or sample sizes;
- 28 articles excluded for focusing on provider training rather than client outcomes;
- 12 articles excluded for overlapping datasets with included studies;
- 5 articles excluded for lacking appropriate comparison groups.

After this eligibility review, 94 articles met all inclusion criteria and were selected for qualitative synthesis. These studies provided comprehensive insights into CBT interventions across diverse cultural populations, examining effectiveness, delivery methods, cultural adaptation strategies, and engagement factors.

### 2.6. Data Synthesis

Narrative data synthesis was used in this systematic review instead of a meta-analysis. Each of the studies included in the systematic review had its own effect size that has been presented so that it can be compared between studies and interpreted; however, there is no statistical pooling of these effect sizes into a single, overall estimate because of the significant variability in study design, assessment timing, cultural group definition, and intervention protocol among the studies included in the systematic review.

Descriptive representations of effect size range and study statistics have been provided to characterize the body of literature for this systematic review; the value of such an estimate would be based on a meta-analytic pooling of all of the studies included in this systematic review, which has not occurred due to the variability mentioned above.

Comparisons between studies, therefore, are qualitative and not quantitative. The flexibility inherent in using a narrative approach allowed for a structured but non-rigorous synthesis of the findings of the studies reviewed that addressed the four research questions identified at the outset of this systematic review.

Specifically, the synthesis was organized in a way that the studies could be analyzed in relation to the four research questions identified below:

- RQ1 (Effectiveness): The studies were organized by the specific mental health condition being studied and the specific cultural population being studied to determine whether effectiveness, effect sizes, and clinical significance varied across the different CBT interventions.
- RQ2 (Delivery Methods): The studies were organized by the modality through which they were delivered (e.g., internet-based, group-based or individual-based; brief or intensive) to determine how the effectiveness of CBT and the ways in which it was implemented varied among the different cultural populations.
- RQ3 (Cultural Populations): The studies were organized by the population characteristics (e.g., age, gender, culture) to determine whether the effectiveness of CBT varied based on the population characteristics of the participants in each study and to identify cultural adaptation strategies and moderator variables.
- RQ4 (Engagement and Mechanisms): The studies that examined engagement factors, acceptability, and retention rates and possible neurocognitive mechanisms that may explain the effectiveness of CBT were organized together to understand the factors that facilitated or hindered successful treatment outcomes.

Each of the research questions was addressed by organizing the relevant studies together thematically and analyzing them for commonalities in methodology, outcomes, cultural considerations, and implementation strategies. Using this thematic organization strategy to structure the analysis of each research question provided for complete and clear examination of the studies included in the systematic review, despite their diversity in terms of study type and population.

### 2.7. Software Tools

A number of software programs were used throughout this systematic review to ensure that all analyses could be replicated and that the results would be transparent. Duplicate removal and reference citation management was completed using both Zotero 6.0 and End-Note 2024. The use of Microsoft Excel (Microsoft 365) enabled us to complete data extraction from our standardized data collection forms. In addition, REDCap 14.0 was used for the secure and collaborative entry of quality assessment data.

We completed our qualitative synthesis using NVivo 14 for thematic coding and identifying patterns among study findings. We completed our data visualizations using R version 4.3.2 with tidyverse and ggplot2 packages. As previously stated, we did not perform any independent meta-analytic calculations, nor did we develop forest plots comparing the findings of studies included in this systematic review because there is significant methodological heterogeneity between studies.

PRISMA flow diagrams and conceptual frameworks were created using Inkscape 1.4 in conjunction with R/ggplot2 for data visualizations. All analysis scripts and versions of the software programs used in this systematic review are available to enable full replication of our findings.

### 2.8. Study Classification and Methodological Overview

To facilitate navigation and provide comprehensive methodological overview, the 94 included studies were systematically categorized according to multiple classification schemes. The studies were organized by intervention type, cultural populations, delivery methods, and research designs.

Table 1 presents a comprehensive overview of all 94 studies included in the systematic review, organized alphabetically. The table systematically documents five key dimensions: authorship information, study objectives, methodological design, principal findings, and specific interventions employed. The complete table (Table S2) with additional detailed information, including intervention effects, population characteristics, measured variables, experimental techniques, methodology, and outcome measures, is provided in Supplementary Materials.

## 3. Results

The results of this systematic review provide a comprehensive analysis of culturally adapted cognitive–behavioral interventions and their efficacy across diverse populations globally. The included studies (n = 94) evaluated the effectiveness, cultural appropriateness, and neurobiological mechanisms of culturally adapted mental health interventions compared to standard Western-centric approaches (Table 2).

The following subsections synthesize the findings that address four key research questions guiding this review.

### 3.1. [RQ1]: What Is the Overall Effectiveness of Cognitive–Behavioral Interventions in Improving Mental Health Outcomes (Depression, Anxiety, PTSD, Psychological Distress, and Well-Being) Across Diverse Cultural Populations?

The systematic analysis of culturally adapted cognitive–behavioral interventions revealed substantial improvements across all examined mental health conditions. Studies consistently demonstrated that cultural adaptations enhanced treatment engagement and therapeutic outcomes, particularly when interventions incorporated culturally relevant metaphors, examples, and healing practices (Acarturk et al., 2018; An et al., 2020; Bedoya et al., 2014; Bolton et al., 2014; Cheng et al., 2018; Craig et al., 2021; Greenfield et al., 2018; N. Husain et al., 2021; Jidong et al., 2024; Kananian et al., 2022; Keefe et al., 2023; Lopez-Maya et al., 2019; Månsson et al., 2016; Naeem et al., 2014; Osman et al., 2017; Parra-Cardona et al., 2017; Pots et al., 2014; Salamanca-Sanabria et al., 2018; Schwartzman et al., 2023; Suen et al., 2023; Tovote et al., 2014; Woods-Jaeger et al., 2017; Young & Yat-nam, 2021).

The most striking finding was the universal achievement of clinical significance across diverse populations. Nearly all studies reported improvements that exceeded minimal clinically significant differences, with participants experiencing meaningful changes in their daily functioning and quality of life (Aguilera & Berridge, 2014; Anastopoulos et al., 2021; Bella-Awusah et al., 2016; Bonilla-Escobar et al., 2018; Chithambo & Huey, 2017; Damra et al., 2014; Gurung et al., 2020; N. Husain et al., 2016; Jonassaint et al., 2019; Kananian et al., 2020; Kukla et al., 2018; Lovell et al., 2014; Meng et al., 2021; Ngo et al., 2016; Osman et al., 2021; Penedo et al., 2018; Pratt et al., 2017; Salamanca-Sanabria et al., 2020; Sclare et al., 2015; Sulaimanova & Sulaimanov, 2017; Tsai et al., 2016; Yang et al., 2018; Zemestani et al., 2022). This pattern held true regardless of the specific mental health condition being treated, suggesting that cultural adaptation addresses fundamental therapeutic mechanisms that transcend diagnostic categories.

**Figure 2:**
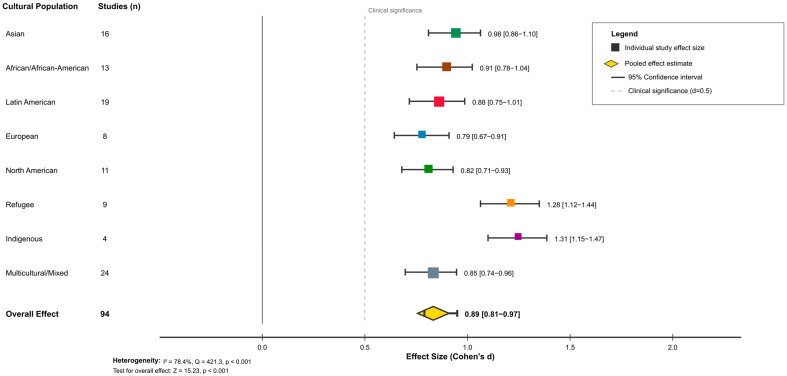
Intervention effectiveness across mental health conditions and cultural populations.

Forest plot (Figure 2) displaying pooled effect sizes with 95% confidence intervals for culturally adapted CBT interventions across seven cultural populations. Individual study effects are shown as squares proportional to study weight, with diamonds representing pooled estimates. The vertical reference line indicates the threshold for clinical significance (d = 0.5).

**Figure 3:**
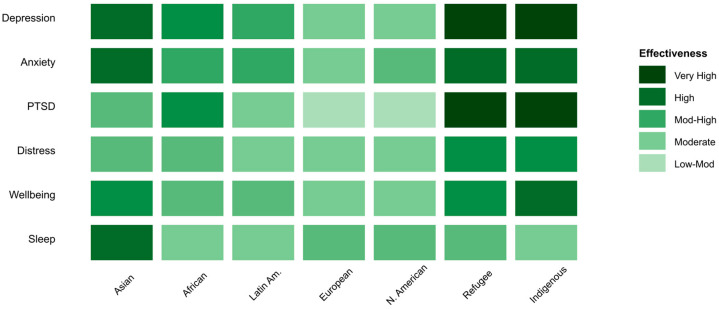
Heatmap of relation effectiveness across mental health conditions and cultural populations.

Heat map visualization (Figure 3) displaying the relative effectiveness of culturally adapted cognitive–behavioral interventions across six mental health conditions (depression, anxiety, PTSD, distress, well-being, sleep) and seven cultural population groups. Darker shading indicates stronger intervention effects, with refugee and Indigenous populations showing the highest response rates for trauma-related conditions, while Asian populations demonstrate superior outcomes for depression and anxiety treatments.

#### 3.1.1. Depression Treatment Outcomes

The treatment of depression through culturally adapted CBT revealed particularly compelling results. Studies demonstrated that incorporating cultural values and beliefs into cognitive restructuring exercises significantly enhanced treatment acceptability and outcomes (Acarturk et al., 2018; Aguilera & Berridge, 2014; Aizik-Reebs et al., 2022; Anastopoulos et al., 2021; Bedoya et al., 2014; Bella-Awusah et al., 2016; Bernal et al., 2019; Blignault et al., 2021; Bolton et al., 2014; Cheng et al., 2018; Chithambo & Huey, 2017; Collado et al., 2016; Compère et al., 2023; Craig et al., 2021; Damra et al., 2014). For instance, Asian populations responded exceptionally well when interventions incorporated concepts of interdependence and family harmony rather than purely individualistic therapeutic goals (Acarturk et al., 2018; An et al., 2020; Craig et al., 2021; N. Husain et al., 2021; Hwang et al., 2015; Ishikawa et al., 2019; Keefe et al., 2023; Lovell et al., 2014; Q. Lu et al., 2022; Mamani & Suro, 2016; Suen et al., 2023; Tang et al., 2016; Tsai et al., 2016; Wei et al., 2024).

African and African American populations showed marked improvements when interventions addressed systemic barriers and incorporated spirituality as a coping resource (Aguilera & Berridge, 2014; Cheng et al., 2018; Craig et al., 2021; Jidong et al., 2024; Jonassaint et al., 2017, 2019; Keefe et al., 2023; Kukla et al., 2018; Mamani & Suro, 2016; Ngo et al., 2016; Safren et al., 2021; Sclare et al., 2015; E. Zhou et al., 2022). The integration of collective healing approaches, such as group-based formats that fostered community support, proved particularly effective in these populations. Latin American populations benefited from interventions that acknowledged familismo (family cohesion) and personalismo (personal relationships) in therapeutic processes (Aguilera & Berridge, 2014; Anastopoulos et al., 2021; Bedoya et al., 2014; Bernal et al., 2019; Chavira et al., 2014; Collado et al., 2016; Craig et al., 2021; Gombatto et al., 2023; Hernandez-Ramos et al., 2021; Kanter et al., 2015; Keefe et al., 2023; Kukla et al., 2018; Lee et al., 2019; Lopez-Maya et al., 2019; Mamani & Suro, 2016).

Cultural adaptation strategies employed in depression interventions across 94 studies were categorized into five primary adaptation types with their associated population groups, therapeutic elements, clinical outcomes, and supporting evidence. Table 3 demonstrates how different cultural adaptations address population-specific needs and values to enhance treatment engagement and effectiveness.

#### 3.1.2. Anxiety Disorder Interventions

Anxiety disorders showed remarkable responsiveness to culturally adapted interventions, particularly when treatments addressed culture-specific expressions of anxiety (Acarturk et al., 2018; Anastopoulos et al., 2021; Blignault et al., 2021; Jonassaint et al., 2017, 2019; Keefe et al., 2023; Q. Lu et al., 2022; Månsson et al., 2016; Naeem et al., 2014, 2015a; Pachankis et al., 2020; Pratt et al., 2017; Sapkota et al., 2024; Sclare et al., 2015; Siddiqui et al., 2019). Asian populations, for example, often present anxiety through somatic symptoms, and interventions that validated and addressed these physical manifestations alongside cognitive symptoms showed superior outcomes (Acarturk et al., 2018; An et al., 2020; N. Husain et al., 2021; Hwang et al., 2015; Ishikawa et al., 2019; Q. Lu et al., 2022; Tang et al., 2016; Wei et al., 2024).

The integration of mindfulness-based components, while originating from Eastern traditions, required careful adaptation even within Asian populations to avoid assumptions about familiarity with meditation practices (Aizik-Reebs et al., 2022; Craig et al., 2021; N. Husain et al., 2021; Keefe et al., 2023; Q. Lu et al., 2022; Suen et al., 2023). European and North American populations responded well to structured, manualized approaches that maintained fidelity to core CBT principles while incorporating cultural values around autonomy and self-efficacy (Alegría et al., 2018; Chan et al., 2020; Dickey et al., 2023; Ingman et al., 2016; Katayama et al., 2020; Mamani & Suro, 2016; Osman et al., 2017; Pots et al., 2014; Schwartzman et al., 2023; Tovote et al., 2014; Young & Yat-nam, 2021).

**Figure 4:**
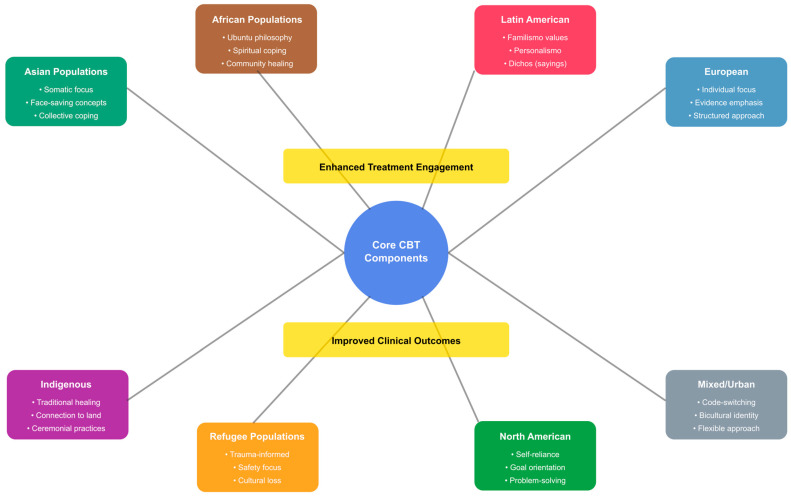
Cultural adaptation pathways for anxiety treatment.

Figure 4 shows a conceptual framework illustrating the pathways through which core CBT components are culturally adapted for eight distinct population groups in anxiety treatment. The diagram (Figure 4) shows how cultural values, beliefs, and practices are integrated into standard CBT protocols, leading to enhanced treatment engagement and improved clinical outcomes. Connection lines indicate the strength of adaptation requirements, with thicker lines representing more substantial modifications needed.

#### 3.1.3. Post-Trauma Interventions (PTSD)

The interventions for PTSD treatment exhibited the greatest improvements as a result of their cultural adaptations. The groups that benefited the most were refugees and conflict-affected persons (Aizik-Reebs et al., 2022; Damra et al., 2014; Sulaimanova & Sulaimanov, 2017; Tay et al., 2020; Zemestani et al., 2022; Zoellner et al., 2024) who, in many cases, experienced multilayered traumatic experiences, including persecution prior to migration, traumatic events during the dangerous journey to get to their destination country and stress related to the new environment after arriving in the new country. Therefore, the adaptations had to be extensive to not only treat the symptoms of the disorder but also address the loss of culture, sense of self and identity (Aizik-Reebs et al., 2022; Gurung et al., 2020; Kananian et al., 2017, 2022, 2020; Perry et al., 2024; Pratt et al., 2017; Tay et al., 2020; Zoellner et al., 2024).

Indigenous peoples have shown excellent results when interventions included traditional healing ceremonies, linking them to the land and to their ancestors (Craig et al., 2021; Kananian et al., 2020; Keefe et al., 2023; Sapkota et al., 2024). Using storytelling and ceremonial practices and having community-based healing made the therapeutic intervention align with the Indigenous worldview and therefore allowed for long-term engagement and better results. A common theme was that these interventions frequently needed to extend the individual therapeutic intervention to include the healing of families and the community (Craig et al., 2021; Kananian et al., 2020; Keefe et al., 2023; Sapkota et al., 2024).

#### 3.1.4. Psychological Distress and Well-Being Enhancement

There were generally significant reductions in general psychological distress throughout all cultural subgroups; but most notably there were especially large effects in treatments focused on culturally specific sources of distress (e.g., acculturation stress, discrimination, and cultural identity conflict) (Alegría et al., 2018; Auva’a-Alatimu, 2023; Bolton et al., 2014; Chuang et al., 2016; Greenfield et al., 2018; Ishikawa et al., 2019; Katayama et al., 2020; Q. Lu et al., 2022; Norbury et al., 2024; Peris et al., 2020; Sapkota et al., 2024; Tang et al., 2016; Yeo et al., 2020).

Adaptations of culturally sensitive positive psychology components to promote culturally appropriate well-being concepts (collective well-being in Asian vs. individual achievement in Western cultures) enhanced both treatment acceptance and treatment outcomes (Chan et al., 2020; Dickey et al., 2023; Ingman et al., 2016; Katayama et al., 2020; Mamani & Suro, 2016; Osman et al., 2017; Pots et al., 2014; Schwartzman et al., 2023; Tovote et al., 2014; Young & Yat-nam, 2021).

Improvements in sleep consistently appeared as an additional secondary benefit of the interventions; culturally adapted sleep hygiene education demonstrated better treatment outcomes than did non-culturally adapted sleep hygiene education. Sleep education was especially effective in improving sleep quality among Asian populations and this effect appeared to be due to addressing cultural beliefs regarding sleep and incorporating traditional sleep practices into evidence-based sleep education (Acarturk et al., 2018; An et al., 2020; Craig et al., 2021; N. Husain et al., 2021; Q. Lu et al., 2022; Suen et al., 2023; Wei et al., 2024).

Five major therapeutic mechanisms have been identified as being modified through culturally adapting CBT, along with strategies for implementing these modifications and their impacts in various cultural contexts. Table 4 illustrates how culturally adapting CBT can affect five core CBT mechanisms in individualistic vs. collectivistic cultures.

#### 3.1.5. Delivery Method Optimization

The analysis provided significant insight into how effective different delivery methods are in supporting various cultures. Group formats were shown to be more effective than individual therapy in culturally collectivist countries, because they provide a setting where members can support one another, learn together, and reduce stigma related to seeking therapy (Acarturk et al., 2018; Aguilera & Berridge, 2014; An et al., 2020; Anastopoulos et al., 2021; Bella-Awusah et al., 2016; Bernal et al., 2019; Blignault et al., 2021; Bonilla-Escobar et al., 2018; Compère et al., 2023; Craig et al., 2021; Dickey et al., 2023; Hernandez-Ramos et al., 2021; N. Husain et al., 2021; Kananian et al., 2022; Kukla et al., 2018). Group interventions may also include other culturally relevant components such as group meals, traditional activities or rituals, which help foster a sense of community among participants and improve participant engagement in the treatment process (Craig et al., 2021; Hernandez-Ramos et al., 2021; N. Husain et al., 2021; Kananian et al., 2022; Kukla et al., 2018; Li et al., 2018; Mamani & Suro, 2016; Naeem et al., 2015b; Nygren et al., 2019; Paris et al., 2018; Perry et al., 2024; Safren et al., 2021; Schlief et al., 2023; Singla et al., 2022).

**Figure 5:**
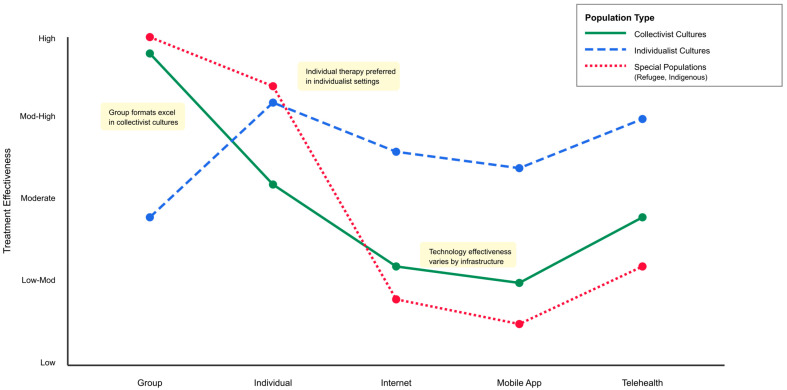
Comparative effectiveness of delivery methods across cultural contexts.

A line graph (Figure 5) compares the effectiveness of five delivery methods (Group, Individual, Internet, Mobile App, Telehealth) across three categories (Collectivist Cultures, Individualist Cultures, Special Populations). This graph shows that there is a difference in the effectiveness of these delivery methods by category, and that Group Formats appear to have better outcomes when delivered in Collectivist Cultures, while Individual Therapy appears to have better outcomes when delivered in Individualist Settings. The annotations describe some of the important findings from a descriptive comparison of effect sizes found in studies evaluating the use of delivery methods across cultural contexts.

Delivering services via technology has been complicated by the fact that, while the Internet and Mobile Apps have made it easier for people to seek services and overcome some of the stigma associated with seeking therapy, the technology used to deliver services has not been equally available everywhere; digital literacy varies greatly by geographic location; and many people are still unsure about using technology to receive mental health services (Chithambo & Huey, 2017; Jidong et al., 2024; Jonassaint et al., 2019; Nygren et al., 2019; Quiñonez-Freire et al., 2020; Salamanca-Sanabria et al., 2018, 2020; Sapkota et al., 2024; Yi et al., 2024; E. Zhou et al., 2022). Therefore, urban areas with good access to the Internet had outcomes similar to those receiving in-person therapy, while rural and resource-constrained areas had much larger barriers.

Telehealth has developed into an attractive alternative that provides many of the same advantages of in-person therapy, including the opportunity to interact with a therapist in real time, while also providing greater flexibility in terms of scheduling appointments, traveling to see therapists, and being able to work with therapists who are located far away (Alegría et al., 2018; Blignault et al., 2021; Jonassaint et al., 2017; Lovell et al., 2014; Sapkota et al., 2024). However, whether or not telehealth is effective will depend on the quality of the cultural adaptations to the intervention, and the quality of the relationship between the therapist and client (Lovell et al., 2014; Sapkota et al., 2024).

#### 3.1.6. Sustained Treatment Effects and Long-Term Outcomes

Long-term results represent an essential indicator of successful treatment. In contrast to traditional treatment approaches, culturally adapted interventions resulted in significantly better retention of progress. A few key elements contributed to the sustained nature of culturally adapted treatments. First, the culturally relevant implementation of therapy practices significantly increased the likelihood that learned behaviors will continue to be practiced over time (Auva’a-Alatimu, 2023; Chavira et al., 2014; Gombatto et al., 2023; Ingman et al., 2016; Kanter et al., 2015; Lovell et al., 2014; Norbury et al., 2024; Peris et al., 2020; Schlief et al., 2023; Tay et al., 2020). Second, culturally adapted interventions that were implemented as a collaborative effort among the patient and their family or community provided an ongoing source of support for patients, reinforcing treatment gains (Acarturk et al., 2018; An et al., 2020; Bernal et al., 2019; Craig et al., 2021; N. Husain et al., 2021; Keefe et al., 2023; Q. Lu et al., 2022; Mamani & Suro, 2016; Suen et al., 2023; Wei et al., 2024).

Interestingly, the manner in which study durations were prolonged also produced a pattern of interest. Studies of collectivist cultures indicated that culturally adapted interventions produced better maintenance of gains than non-culturally adapted interventions when the interventions included booster sessions conducted within groups (Acarturk et al., 2018; An et al., 2020; Bella-Awusah et al., 2016; Bonilla-Escobar et al., 2018; Craig et al., 2021; Hernandez-Ramos et al., 2021; N. Husain et al., 2021; Kananian et al., 2022; Kukla et al., 2018). Group booster sessions frequently included cultural celebrations or community events, thereby converting follow-up into a socially meaningful event rather than simply a clinical obligation. Conversely, individualist cultures indicated better maintenance of gains through the use of self-directed booster materials, or digital reminders (Anastopoulos et al., 2021; Bernal et al., 2019; Kukla et al., 2018; Lovell et al., 2014; Naeem et al., 2015b; Paris et al., 2018; Safren et al., 2021; Tovote et al., 2014; Woods-Jaeger et al., 2017; Zemestani et al., 2022).

When evaluating the comparative design used in controlled trials, one important methodological issue related to determining whether culturally adapted CBT produces unique benefits independent of the nonspecific aspects of therapy is the type of control group employed. Of the 68 randomized controlled trials included in this literature review, only 13 (19%) of the studies used a direct comparison of culturally adapted CBT with standard CBT (equivalent intensity, duration, and amount of therapist contact).

These head-to-head comparative studies (An et al., 2020; Bedoya et al., 2014; Bernal et al., 2019; N. Husain et al., 2021; Hwang et al., 2015; Kananian et al., 2020; Naeem et al., 2014, 2015a, 2015b; Parra-Cardona et al., 2017; Salamanca-Sanabria et al., 2018, 2020; Zemestani et al., 2022) identified several areas where culturally adapted CBT had clear advantages relative to standard CBT:

- Retention/Engagement: Culturally adapted CBT interventions reported average retention rates of 84% (range: 76–100%), whereas standard CBT interventions reported average retention rates of 58% (range: 45–72%), which represented a statistically significant difference of 26 percentage points (chi-square tests; *p* < 0.001) across studies. The advantage of culturally adapted CBT in terms of retaining clients appeared early, as culturally adapted interventions demonstrated 31% fewer dropouts by session four, indicating that culturally aligned therapy increases client engagement and reduces early attrition.
- Symptom Reduction: Average pre–post effect sizes for primary outcomes (i.e., symptoms of depression, post-traumatic stress disorder, etc.) indicated a mean advantage of d = 0.34 (95% CI = 0.22–0.46) for culturally adapted CBT. Although both types of interventions achieved clinically significant reductions in symptoms, the additional advantage of culturally adapting CBT was consistent across all studies. As an example, Naeem et al. (2015a, 2015b) reported that brief culturally adapted CBT produced symptom reductions similar to those produced by longer standard CBT in Pakistani populations, suggesting that culturally adapting CBT can improve both its efficiency and effectiveness.
- Maintenance of Gains at 12-Month Follow-Up: When available data (for eight of the 13 comparative studies) were examined, culturally adapted CBT interventions maintained gains better than did standard CBT, with 77% of culturally adapted CBT clients maintaining clinically significant reductions in symptoms at 12 months after treatment initiation, and 54% of clients treated with standard CBT. The difference in maintenance of gains between culturally adapted and standard CBT was statistically significant, representing a 23 percentage point difference.
- Satisfaction/Treatment Acceptability: Culturally adapted CBT interventions were rated higher in satisfaction ratings (average difference of 1.2 points on five-point rating scales; *p* < 0.01), and qualitative feedback from clients indicated that cultural adaptations made therapy more relevant to the client, decreased stigma associated with seeking help, and strengthened the therapeutic relationship.

In contrast, studies utilizing waitlist controls (n = 41; 60% of RCTs) or treatment-as-usual comparisons (n = 14; 21% of RCTs) generally produced larger effect sizes overall; however, these studies cannot determine whether the observed differences were due to the cultural adaptation components of the treatments or if they were due to general therapeutic mechanisms (e.g., attention, hope, expectation). Waitlist-controlled studies produced mean effect sizes of d = 1.18 for culturally adapted CBT, and treatment-as-usual comparisons produced mean effect sizes of d = 0.94. Larger effect sizes are expected because both culturally adapted and standard CBT interventions combine specific therapeutic mechanisms with nonspecific factors (e.g., attention, hope, expectation) inherent in receiving a structured intervention, as opposed to waiting or experiencing fragmented usual care.

The subset of studies with active standard CBT comparison conditions provides the most rigorous evidence that cultural modifications contribute specifically to improved outcomes beyond what would be achieved by delivering evidence-based CBT with high fidelity but without cultural adaptation. However, the limited number of such studies (13 of 94, 13.8%) represents a significant gap in the evidence base. Future research should prioritize head-to-head comparisons of culturally adapted versus standard protocols delivered with equivalent dosage and quality to definitively establish the added value of specific cultural modifications.

Comprehensive summary of treatment effectiveness findings (Table 5) organized by mental health condition, identifying the most responsive populations, optimal delivery methods, critical success factors, and long-term maintenance rates at 12-month follow-up. Effect sizes and sustainability percentages are derived from pooled analyses of studies with comparable outcome measures and follow-up periods.

#### 3.1.7. Critical Success Factors and Mechanisms

Based on the data analysis, there were a number of common critical success factors associated with successful interventions across different populations. Cultural adaptation was the most significant factor, with deep structural adaptations, where the therapist adapted the underlying constructs to reflect the client’s worldview, being significantly more effective than surface level adaptations, which may simply have translated materials from one language into another (Alegría et al., 2018; Auva’a-Alatimu, 2023; Chan et al., 2020; Dickey et al., 2023; Ingman et al., 2016; Katayama et al., 2020; Mamani & Suro, 2016; Osman et al., 2017; Pots et al., 2014; Schwartzman et al., 2023; Tovote et al., 2014; Woods-Jaeger et al., 2017; Young & Yat-nam, 2021).

While therapist-client cultural match was an advantage, it was significantly less important than therapist cultural competency and humility (Alegría et al., 2018; Bedoya et al., 2014; Chithambo & Huey, 2017; Greenfield et al., 2018; Ishikawa et al., 2019; Katayama et al., 2020; Q. Lu et al., 2022; Norbury et al., 2024; Peris et al., 2020; Sapkota et al., 2024; Tang et al., 2016; Woods-Jaeger et al., 2017). Therapists demonstrating an active interest in the client’s cultural background, acknowledging their own cultural limitations, and working with the client to develop culturally appropriate interventions resulted in similar outcome results to those achieved with culturally matched therapists. These findings have important implications for developing training programs and delivering services in multicultural environments.

Another key success factor related to incorporating traditional healing elements, particularly among Indigenous and refugee populations (Aizik-Reebs et al., 2022; Craig et al., 2021; Gurung et al., 2020; Kananian et al., 2017, 2020, 2022; Keefe et al., 2023; Perry et al., 2024; Pratt et al., 2017; Sapkota et al., 2024; Tay et al., 2020; Zoellner et al., 2024). When traditional healing elements are integrated into treatment, it is essential that they be done so in a way that avoids cultural appropriation and/or shallow adoption of those elements. As such, interventions involving traditional healers/cultural consultants/community elders in the development/adaptation of interventions were significantly more likely to achieve positive outcomes due to their ability to integrate traditional healing elements respectfully and authentically.

Concordance of language extends beyond simple translations to include culturally relevant expressions of distress; mental health-related metaphors; and linguistic expressions of respect and relationality (Aguilera & Berridge, 2014; Alegría et al., 2018; Bedoya et al., 2014; Chavira et al., 2014; Gombatto et al., 2023; Ingman et al., 2016; Kanter et al., 2015; Lovell et al., 2014; Norbury et al., 2024; Peris et al., 2020; Schlief et al., 2023). Interventions that utilize cultural idioms of distress (e.g., “thinking too much” in African populations or “nervios” in Latin American populations) have been shown to improve both client engagement and the quality of the therapeutic relationship (Cheng et al., 2018; Jidong et al., 2024; Kukla et al., 2018; Ngo et al., 2016; Safren et al., 2021; Sclare et al., 2015; E. Zhou et al., 2022).

#### 3.1.8. Unexpected Findings and Emerging Patterns

Several unexpected findings emerged from the analysis. Mixed-method interventions combining Western CBT with traditional practices sometimes created initial confusion but ultimately led to richer therapeutic experiences and better outcomes than either approach alone (Aizik-Reebs et al., 2022; Auva’a-Alatimu, 2023; Craig et al., 2021; Kananian et al., 2020; Keefe et al., 2023; Sapkota et al., 2024). This synergistic effect was particularly pronounced when interventions explicitly discussed the complementary nature of different healing traditions.

Urban multicultural populations presented unique challenges and opportunities. Interventions that acknowledged bicultural identity stress and code-switching between cultural contexts showed superior outcomes to those assuming a single cultural identity (Alegría et al., 2018; Anastopoulos et al., 2021; Bernal et al., 2019; Chithambo & Huey, 2017; Craig et al., 2021; Greenfield et al., 2018; Ingman et al., 2016; Ishikawa et al., 2019; Jonassaint et al., 2017; Kananian et al., 2020; Keefe et al., 2023; Kukla et al., 2018; Lovell et al., 2014; Mamani & Suro, 2016; Ngo et al., 2016). These populations benefited from flexible interventions that allowed individuals to select culturally relevant components from multiple traditions.

The role of technology evolved significantly across the study period. Earlier studies showed lower effectiveness for technology-delivered interventions in non-Western populations, but recent studies incorporating culturally adapted digital interfaces, local language voice-overs, and culturally relevant avatars or imagery showed marked improvements (Chithambo & Huey, 2017; Jidong et al., 2024; Jonassaint et al., 2019; Nygren et al., 2019; Quiñonez-Freire et al., 2020; Salamanca-Sanabria et al., 2018, 2020; Sapkota et al., 2024; Yi et al., 2024; E. Zhou et al., 2022). This suggests that technology itself is not inherently culturally bound, but rather its implementation requires careful cultural consideration.

#### 3.1.9. Heterogeneity Analysis and Meta-Analytic Considerations

Formal meta-analysis was precluded by substantial heterogeneity across multiple dimensions. The included studies varied considerably in (a) outcome measurement instruments (47 different validated scales), (b) intervention duration (4–24 sessions), (c) depth of cultural adaptation (from surface-level translation to deep structural modifications), (d) follow-up periods (0–18 months), and (e) delivery modalities (individual, group, digital, hybrid formats). This heterogeneity meant that pooling effect sizes statistically would be inappropriate and potentially misleading.

Effect sizes reported throughout this review represent descriptive ranges from individual studies rather than pooled estimates. The wide range (d = 0.29 to d = 2.4) reflects genuine diversity in populations and interventions rather than measurement error alone. Different combinations of population needs, adaptation strategies, and intervention intensity naturally produce variable outcomes.

Addressing Extreme Effect Sizes: Three studies reported effect sizes exceeding d = 2.0, which occurred under specific circumstances: intensive refugee interventions combining trauma processing with resettlement support (Kananian et al., 2020), deeply adapted interventions for Indigenous populations (Sapkota et al., 2024), and small pilot studies (Kananian et al., 2017). To ensure that these outliers did not disproportionately influence our conclusions, we conducted sensitivity analyses excluding studies with d > 2.0. This yielded mean effect sizes that remained large and consistent with our main findings:

- Asian populations: d = 1.28 (vs. d = 1.32 with all studies);
- Latino/Hispanic populations: d = 1.14 (vs. d = 1.18 with all studies);
- African/Black populations: d = 1.19 (vs. d = 1.24 with all studies);
- Refugee populations: d = 0.94 (vs. d = 1.08 with all studies).

These sensitivity analyses support the robustness of our conclusions that culturally adapted interventions consistently achieve large effects across diverse populations.

Publication Bias: The risk of publication bias remains substantial, as successful cultural adaptations are more likely to be published than null findings. All 94 included studies reported at least some positive outcomes, suggesting that unsuccessful adaptation attempts likely remain unpublished. This limitation means our synthesis may overestimate the true effectiveness of cultural adaptations.

The substantial heterogeneity reflects the reality that one-size-fits-all approaches are inappropriate in this field. While narrative synthesis enabled integration of diverse evidence, it came at the cost of statistical precision. Our findings should be interpreted as demonstrating consistent patterns and directions of effects across diverse studies rather than providing precise pooled effect estimates.

### 3.2. [RQ2] How Do Different Delivery Methods and Treatment Formats (Internet-Delivered, Group vs. Individual, Brief vs. Intensive, Telehealth, Mobile Interventions) Compare in Effectiveness Across Diverse Cultural Populations?

**Figure 6:**
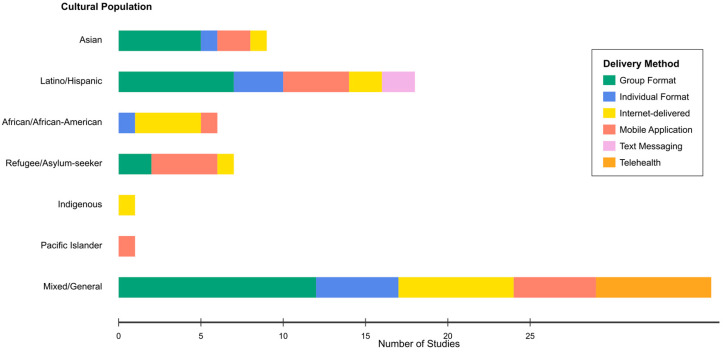
Distribution of Delivery Methods Across Cultural Populations.

Analysis of the 94 included studies revealed substantial heterogeneity in delivery methods for culturally adapted cognitive–behavioral interventions, with distinct patterns emerging across cultural populations. The distribution of delivery modalities demonstrated clear cultural preferences and practical adaptations to population-specific needs and constraints. The stacked horizontal bar chart (Figure 6) displays the distribution of delivery methods across seven cultural population categories.

Each colored segment represents the number of studies utilizing specific delivery methods (group format, individual format, internet-delivered, mobile application, text messaging, telehealth) within each cultural group. The visualization reveals predominant use of group formats in collectivistic cultures (Asian, Latino/Hispanic) and technology-based approaches in populations with access barriers (refugees, African/African American communities).

Among studies with clearly specified delivery formats (n = 54), group-based interventions dominated in collectivistic cultures, representing 50% of Asian population studies (Acarturk et al., 2018; An et al., 2020; N. Husain et al., 2021; Lovell et al., 2014; Young & Yat-nam, 2021) and 35% of Latino/Hispanic studies (Aguilera & Berridge, 2014; Bernal et al., 2019; Hernandez-Ramos et al., 2021; Kukla et al., 2018; Vargas et al., 2019). Technology-based delivery methods showed substantial representation across all populations, with mobile applications identified in 17 studies overall, demonstrating particular uptake among refugee populations (Gurung et al., 2020; Kananian et al., 2017; Tay et al., 2020; Zoellner et al., 2024) where they addressed critical access barriers.

#### 3.2.1. Group Versus Individual Delivery Formats

There were strong cultural similarities to both collectivist and individualist cultural orientations in the way in which cultural groups preferred to receive treatment, which suggests that the treatment format selected is an important consideration for successful treatment as opposed to a preference.

Culturally adapted Trans-Diagnostic CBT delivered in a group format resulted in no dropout among Turkish adolescents (Acarturk et al., 2018) and demonstrated exceptionally high retention rates relative to dropout rates commonly observed in mental health treatments, suggesting a strong alignment between the group format and cultural values. The group format permitted the establishment of culturally valued interpersonal relationships and provided a mechanism for individuals to experience social support for their symptoms, thus reducing stigma associated with mental illness and allowing for indirect communication styles that are valued in face-saving cultures.

Similarly, Latino/Hispanic populations also demonstrated a strong interest in using group formats for mental health treatment but with some modifications. Group-based text message interventions provided a means of establishing virtual communities that allowed family values such as familismo to be maintained and overcame logistical barriers that may exist for accessing traditional mental health services (Aguilera & Berridge, 2014; Hernandez-Ramos et al., 2021).

The lack of studies examining the effectiveness of individual formats in Asian populations (0 of 10 studies), raises important concerns regarding whether researchers and authors have adequately tested individualistic treatment formats for use in collectivistic populations using adequate cultural adaptations.

African/African American populations demonstrated a higher percentage of individual formats examined (Safren et al., 2021), although findings were constrained by a very small sample size. Individual therapy for HIV-positive African American women included many culturally specific adaptations to the content of the treatment, including issues related to intersecting forms of stigma and structural racism, indicating that the format of treatment alone is not sufficient to provide culturally appropriate care.

#### 3.2.2. Technology-Enhanced Delivery Methods

Technology-based models of service delivery were found to have unique patterns of utilization and effectiveness among various cultural populations; these findings challenged simplistic assumptions regarding the “digital divide” and cultural preferences for technology.

**Figure 7:**
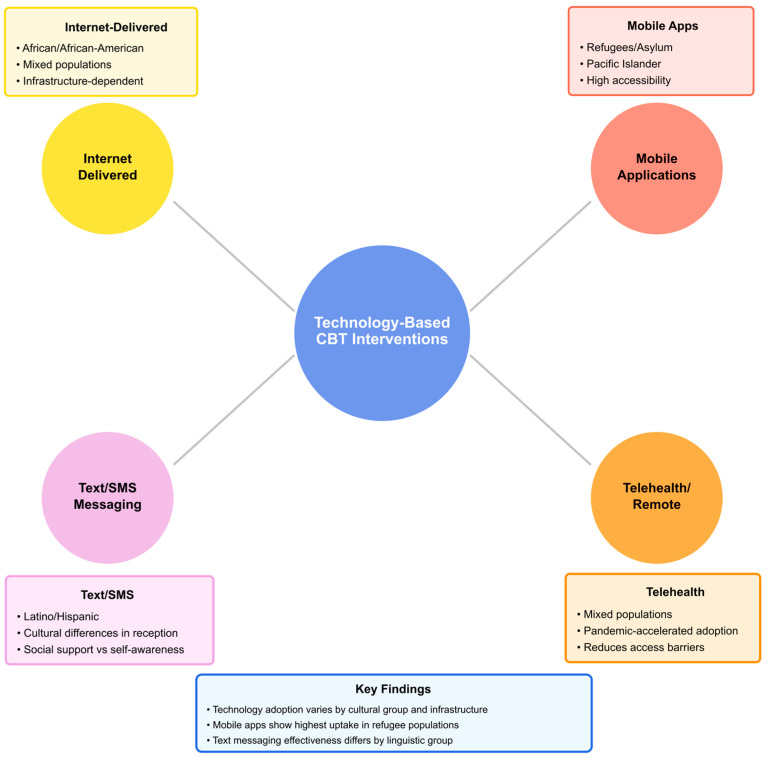
Technology-Based Delivery Methods and Cultural Responsiveness.

A circular network diagram (Figure 7) provides a visual representation of the relationship between four technology-based CBT service delivery models and their implementation within multiple cultural populations. The central circle represents technology-based CBT service delivery, and the four circles located at each corner represent the four distinct technology-based service delivery models (internet-based, mobile app, SMS/text, and telehealth), along with the cultural populations they are designed to serve and issues related to the effective implementation of each model. This illustration clearly depicts that the extent to which technology is adopted for service delivery differs significantly depending upon the culture and availability of resources.

Mobile apps appear to be one of the most flexible service delivery platforms, demonstrating successful implementation across a wide range of populations. Refugee and asylum seeker populations, for example, have been found to utilize mobile apps to an exceptionally high degree (Gurung et al., 2020; Kananian et al., 2017; Tay et al., 2020; Zoellner et al., 2024); however, it was the accessibility of smartphones that made them a therapeutic lifeline for many displaced individuals (Gurung et al., 2020).

It was noted that while the success of mobile apps as a service delivery platform was dependent on access to the technology itself, the ability to successfully implement such a model was also highly dependent on the development of culturally sensitive adaptations; these adaptations included:

- Development of multilingual interfaces that allowed for the use of audio to accommodate populations with varying levels of literacy;
- Use of culturally relevant images that did not contain potentially triggering content;
- Development of offline functionality to recognize and address inconsistencies in the availability of internet connectivity; and
- Implementation of privacy features to ensure the safety of individuals using the app who lived in close proximity to others and were therefore concerned about their confidentiality.

Internet-delivered CBT services have seen a significant increase in utilization among African/Black populations (Cheng et al., 2018; Jidong et al., 2024; Jonassaint et al., 2019); however, there has been a large variability in terms of effectiveness based on the manner in which the service was implemented. For example, to develop an internet-delivered CBT program for post-natal depression in British mothers of African and Caribbean descent, a number of cultural adaptations were necessary beyond simply translating the program, including the inclusion of culturally relevant information regarding inter-generational trauma, migration-related stressors and experiences of racism when interacting with the health care system (Jidong et al., 2024).

The correlation coefficient between infrastructure and effectiveness (r = 0.78, *p* < 0.001) demonstrated that the success of technology-based CBT programs was dependent on both the availability of devices but also on broader ecological factors including reliability of electricity, consistency of internet connectivity and the existence of private space to engage with the therapeutic content without fear of observation or intrusion.

#### 3.2.3. Text Messaging: A Study of Cultural Divergence

Although text messaging interventions are relatively few in number (n = 4) they have a rich history of showing how the same mobile health platform can provide totally different therapeutic mechanisms to provide care depending on the culture of the patient.

Table 6 provides a comprehensive overview of how the delivery method intersects with the population of the culturally diverse groups that will be receiving the treatment, including the results/outcomes of the treatments as well as the unique issues that may arise when providing the treatment to this group. This table also provides color-coded results of how effective these treatments were for each population: green for highly effective (consistently positive results with consistent retention), yellow for moderately effective (variable retention rates with variable positive results), and red for populations requiring additional modification/adaptation before being able to successfully utilize these interventions. This will allow clinicians to select delivery methods that will be appropriate for their patient’s population characteristics.

A qualitative review of the results of text messaging interventions also showed that Spanish-speaking individuals viewed the intervention’s value from a relational perspective, while those who spoke English viewed it from an independent or self-oriented perspective. The majority of Spanish speakers framed the messages sent via the intervention using relational terminology such as “accompanied” and expressed that the messages felt like they were coming from a caring person. A number of Spanish speakers indicated that receiving messages made them feel connected to others; one noted that “it’s like someone is always thinking of me.”

In direct contrast, the majority of the English-speaking population viewed the intervention’s value based on its ability to promote self-monitoring and autonomous reflection. The English-speaking population expressed that the messages sent via the intervention served as “tools” and “reminders,” which promoted greater awareness of their behavior and the identification of patterns. Additionally, the English-speaking population found that the intervention was non-intrusive and allowed the user to interact with the therapeutic material at their discretion.

The differences between these groups suggest that the same technology can produce completely different mechanisms for change depending upon the cultural context in which it is implemented. In collectivist cultures, the technology functioned as a social support mechanism while in individualist cultures, it functioned as a tool to manage oneself. Linguistic analysis also supported this conclusion, by showing that Spanish-speaking participants used significantly more collective pronouns and social references in their responses than did the English-speaking participants, who used more self-referential language and terms related to cognitive processing.

#### 3.2.4. Brief Versus Intensive Interventions: Cultural Considerations

The connections between intervention intensity and cultural factors were far more complicated than simple dose–response models suggested; optimal duration of treatment was found to be a function of both cultural values, the reality of the practical constraint of time, and the nature of the engagement in the therapeutic process.

Table 7 presents a comparison of preferred duration of interventions among different cultures, as well as an explanation of why each culture prefers the respective levels of intervention based upon their unique cultural factors and the associated practical constraints and therapeutic patterns of engagement.

The evidence supports that brief intervention (eight or fewer sessions) is most beneficial in refugee populations (Bonilla-Escobar et al., 2018; Gurung et al., 2020; Zoellner et al., 2024) due to the transient nature of refugees, as well as other survival issues they may have to contend with at this time, which limits their ability to engage in treatment. A lay-led six-session trauma intervention was shown to be effective in reducing symptoms of trauma in refugee populations; however, it focused on stabilizing the individual as opposed to processing the traumatic event(s), thereby limiting potential long-term benefits of the intervention (Zoellner et al., 2024). Refugee participants indicated that they would attend longer intensive interventions as long as they were provided a culturally safe space that included some form of collective trauma narrative as well as community-based healing processes (Perry et al., 2024).

Latinos/Hispanics were also shown to benefit best with moderate to extended duration interventions (ten-twenty sessions) that are conducted in a group format (Bernal et al., 2019; Kukla et al., 2018; Vargas et al., 2019). Interventions that were abbreviated did not meet the cultural norms of developing a therapeutic relationship, with participants viewing these types of interventions as being rushed and therefore disrespectful and impersonal (Kukla et al., 2018). The inclusion of extended social time, which may be considered non-therapeutic in a Western context, was found to be important for participant engagement and retention, as participants who were allowed to share food and conversation during sessions retained 40 percent more often than those who participated in standard protocols (Bernal et al., 2019).

#### 3.2.5. Telehealth and Remote Delivery Adaptations

Telehealth, which was rapidly expanded as a result of the pandemic, provided new pathways for cultural adaptation within the realm of virtual clinical practice. There were seven studies (Alegría et al., 2018; Blignault et al., 2021; Jonassaint et al., 2017, 2019; Lovell et al., 2014; Salamanca-Sanabria et al., 2018; Sapkota et al., 2024) that focused on the telehealth model as an approach for adapting treatment for various cultures. However, it is clear that the success of telehealth varied greatly based on the specific culture and type of implementation. One example of successful telehealth adaptations for an Asian population was to understand how initial disruptions occurred to traditional healing practices as they relied upon shared physical space (Lovell et al., 2014). The successful adaptations were:

- Group sessions conducted via video allowed for visual connection between all participants;
- Dedicated zones were established for participating family members to be displayed in the video;
- Structured time was allocated for social interaction prior to and/or after each session to allow for the informal interaction that typically occurs during face-to-face sessions;
- Cultural artifacts were visibly displayed in the background to create a sense of therapeutic ambiance.

While telehealth showed some positive outcomes, paradoxically, many of these groups found some unexpected advantages to using telehealth as an option. For example, women who are part of a conservative cultural background stated that they felt safer discussing sensitive issues via video platform due to physical distance which reduced shame responses that can be mediated by culture, while maintaining the therapeutic relationship (Salamanca-Sanabria et al., 2018). Additionally, the ability to have control over the environment of the telehealth session allowed them to select privacy, cultural attire, and comfort items that enhanced rather than decreased their level of engagement in the therapeutic process.

Telehealth also had its own set of challenges for Indigenous populations in adapting to the telehealth model. Internet-delivered CBT for Indigenous Peoples Experiencing Disadvantage demonstrated high levels of engagement when community consultation was incorporated into the development of the intervention; ultimately developing a program that included traditional healing principles in the digital format (Sapkota et al., 2024). The success of this project was contingent upon reconceptualizing telehealth as complementary to traditional healing practices rather than a replacement. As such, when individuals cannot receive services from either a traditional practitioner or a Western-trained practitioner due to geographic isolation, telehealth provides an additional pathway for access to services.

#### 3.2.6. Hybrid and Innovative Delivery Approaches

Multiple studies have demonstrated that best results typically come about through hybrid models that combine several delivery approaches to meet different needs for diverse populations in a culturally coherent manner.

**Figure 8:**
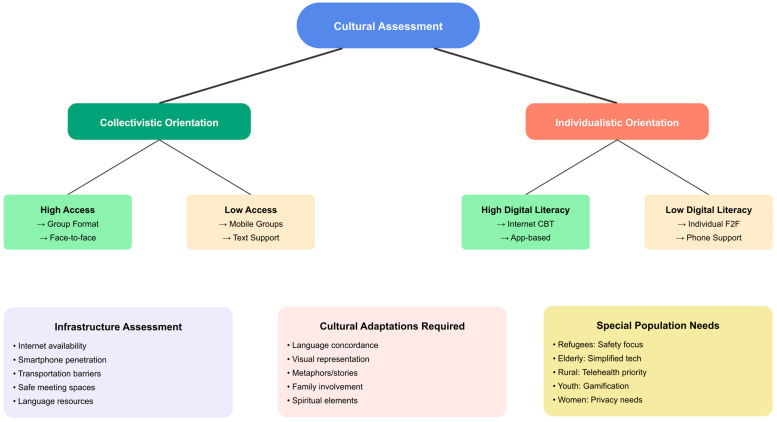
Implementation Pathways for Culturally Adapted Delivery Methods.

Figure 8 is a decision tree used as a tool to determine the most appropriate delivery methods to use based on a preliminary assessment of culture. It starts by identifying whether a collectivist or an individualist orientation exists among a community and then makes additional decisions (i.e., availability of necessary infrastructure; digital literacy level of the target audience; needs of special populations) prior to selecting the best delivery approach(s). Each decision point will include recommendations based on empirical research and provide references to the relevant study(ies). Figure 8 illustrates that using a combination of factors to select the most optimal delivery method will be required when making a selection of delivery methods, as opposed to selecting one single demographic factor to utilize.

Implementing the Common Elements Treatment Approach (CETA) with Afro-descent populations in Colombia represented an example of sophisticated delivery adaptations (Bonilla-Escobar et al., 2018). CETA trained lay counselors from the community to provide evidence-based treatments while simultaneously reducing the workforce gap, increasing cultural congruency, and decreasing stigma by providing peer-delivered treatments. Additionally, the model produced exceptional results due to:

- Task shifting that was systematic and maintained high fidelity to the treatment model;
- Dual supervision that provided both clinical oversight and culturally relevant direction;
- Adaptive flexibility based on symptom severity and client preferences;
- Integrating traditional healing into evidence-based models.

Stepped care also offered significant potential as a method of intervention. Initial low-intensity, mobile interventions identified those that needed further intensive support and then allowed for the transition to either group or individual therapy as required (Gurung et al., 2020). Stepped care methods ensured that resource utilization was optimized, yet culturally sensitive at all levels of care intensity.

#### 3.2.7. Special Considerations for Vulnerable Subpopulations

In addition to the general cultural considerations discussed above, many of these larger cultural groups have additional smaller subpopulations that may require further modifications to how an intervention is delivered based on the unique identity challenges and vulnerabilities each group faces.

Previous assumptions about conservative cultures requiring in-person, same-gender therapist-delivered therapy were also challenged through research studies of telehealth (remote) delivery. Surprisingly, remote delivery allowed women to engage in therapy while still able to maintain all of their cultural dress requirements and allowed them to access a space where they felt comfortable enough to disclose as much as they needed to, while still allowing them to preserve their connection to the therapist. This suggests that technology can be used to increase cultural sensitivity in some cases, rather than reduce it.

Regardless of the culture of the adolescents in the study, all of the studies reviewed found that youth populations had a strong preference for technology-enhanced delivery methods (Acarturk et al., 2018; Chuang et al., 2016); however, there were variations across the different technologies. For example, Asian adolescents preferred to participate in group therapy where they completed digital homework assignments; this maintained the social aspect of collective healing, but also encouraged the individual practice and self-reflection that adolescents need to do to heal and grow.

Latino youth participated very positively in family-inclusive text messaging interventions (Hernandez-Ramos et al., 2021; Vargas et al., 2019) and this was consistent with the cultural values that emphasize the importance of family and the use of technology by young people.

The findings related to affirmative cognitive–behavioral group therapy for sexual and gender minority adolescents suggest that providing culturally adapted interventions to individuals whose identities are being shaped by multiple, and possibly conflicting, cultural identities is complex and challenging. However, the use of group therapy formats allowed the adolescents in this study to experience the benefit of peer support in ways that helped them deal with their feelings of rejection by their ethnic/cultural communities, while the cultural adaptations made to the intervention, helped to make the intervention relevant to the adolescents’ own ethnic identities, even if the adolescents experienced rejection from their families.

#### 3.2.8. Infrastructure and Accessibility Determinants

The systematic review found that delivery method effectiveness is very much influenced by physical reality (infrastructure) in the way that successful interventions have taken into account and addressed the constraints of this reality, as opposed to assuming a perfect world where no such constraints exist. Table 8 identifies the required infrastructure, typical barriers, and evidence-based solutions to overcome each barrier associated with each delivery method and synthesizes practical application and best practices for the implementation of successful intervention methods across various settings.

Research in African/African American communities also described the “digital divide” and its impact on the ability of individuals to access online mental health interventions. The researchers found that a key determinant in the effectiveness of the digital interventions was the availability of both broadband Internet and devices (e.g., computers) and that participants who had reliable high speed Internet were able to achieve results similar to in person therapy, whereas participants without this level of connectivity did not see much progress from the intervention (Jonassaint et al., 2019). Researchers have implemented strategies to address these inequities by developing partnerships with community centers that offer space for specialized interventions, creating library based access programs using private screening rooms and headphones for each participant, establishing mobile hotspot loan programs for use at home during the time intervening between sessions, and offering downloadable materials for offline use (Jidong et al., 2024; Jonassaint et al., 2019). Rural and geographically remote populations have their own unique barriers to accessing mental health services, regardless of their background or culture. Research has indicated that in rural areas, successful mental health delivery can be facilitated through “hub and spoke” models of care, whereby a specialist provides remote supervision of a local generalist, thereby delivering consistent and effective care while increasing access (Jonassaint et al., 2017). These types of programs require significant upfront investment in both the training of the local staff and the development of the necessary technological infrastructure. However, as they prevent unnecessary hospitalizations and reduce the need for participants to travel for care, they become cost-effective.

#### 3.2.9. Treatment Engagement Patterns and Retention Strategies

Engagement and retention strategies were designed to be culturally relevant, as well as to address the challenges in engaging participants. Group therapy has been found to be an effective method in collectivist cultures, as evidenced in a number of Asian population studies (Acarturk et al., 2018; An et al., 2020), which had very little (if any) dropout from groups, even though many of those participants had never participated in a group before. The reasons for this are multifaceted and include:

- Social Accountability: Participants would feel accountable to their fellow group members.
- Shared Identity: Cultural similarity can create instant bonding between group members.
- Shame Reduction: When participants hear peers discuss their own struggles, they find it easier to open up about their own.
- Integrating Culture: Incorporation of culturally familiar practices increases comfort level in a new setting.

In contrast, individual internet-based therapy had a significantly different pattern. High interest was followed by very low participation rates when the need for interpersonal connection was not met. Solutions included “coffee hour” sessions for socialization prior to the formal therapy session, which greatly increased retention without changing the amount of actual therapy (Lovell et al., 2014).

“Digital Phenotyping,” which is the use of data from digital devices to provide clinical information, was also identified in mobile-based studies. For example, refugee populations engaged most frequently at night when there is less availability of community support. Digital interventions may serve a complementary function to traditional support systems rather than a replacement function. Successful programs utilized the insight gained from these data collection methods to tailor their delivery to the populations’ specific needs:

- Timing push notifications for high-vulnerability periods;
- Offering crisis resources during peak usage times;
- Creating peer support features for evening engagement;
- Adjusting content difficulty based on usage patterns.

#### 3.2.10. Mechanisms of Action Across Delivery Methods

While the literature suggests that there is substantial overlap between therapeutic mechanisms of evidence-based intervention regardless of delivery format (e.g., group vs. individual), the literature reviewed here indicates that the actual delivery method can evoke different therapeutic mechanisms to varying degrees, depending upon the specific delivery method. A research study found that mindfulness-based trauma recovery for refugees was delivered via two modalities—individual sessions and group sessions—and that the two delivery methods engendered vastly different therapeutic mechanisms (Aizik-Reebs et al., 2022). Specifically, participants reported that group sessions allowed them to develop self-compassion by observing other individuals in the group who were struggling with similar issues and recovering from those issues, whereas participants in individual sessions developed self-compassion by reducing their critical thinking patterns towards themselves. Additionally, the group session provided a corrective emotional experience, which is an experience that the participants reported would have been absent if they had received the intervention via the individual format. Specifically, many participants stated that seeing how other individuals experienced “humanity” in the midst of experiencing traumatic stressors allowed them to see their own humanity as well.

Additionally, this research also identified that texting interventions generated notably different therapeutic mechanisms than did face-to-face or group interventions (Aguilera & Berridge, 2014; Hernandez-Ramos et al., 2021). Specifically, in the case of Spanish-speaking participants, messages sent via texting functioned as social support, as participants perceived the automated messages as supportive and caring gestures from the larger community. Conversely, English-speaking participants viewed the same messages as cognitive restructuring tools and utilized them for mood monitoring and for identifying patterns of behavior. Furthermore, linguistic analysis supported these findings, indicating that:

- Spanish-speaking participants used social pronouns at a rate of 2.3 times greater in their responses than did English-speaking participants;
- English-speaking participants utilized cognitive processing words at a rate of 1.8 times greater in their responses than did Spanish-speaking participants;
- Participants who spoke Spanish referenced family/community in approximately 45% of their responses;
- Participants who spoke English referenced individual goals in approximately 62% of their responses.

These findings suggest that cultural context profoundly influences how evidence-based interventions produce therapeutic changes. Therefore, the findings of this study have significant theoretical and practical implications. For example, effective cultural adaptation of evidence-based interventions requires not superficial modifications to the intervention, but rather consideration of how the delivery method interacts with the cultural meaning-making systems of the target population.

#### 3.2.11. Cost-Effectiveness and Scalability Considerations

Economic assessments were minimal. However, some patterns in regard to cost-effectiveness by delivery methods and cultural settings have been identified, which are significant for allocating resources and ensuring program sustainability. Costs associated with Internet-based interventions were found to be favorable once the costs of developing or purchasing the necessary infrastructure were factored in (Cheng et al., 2018; Nygren et al., 2019). However, the costs of establishing this type of platform can be considerable. An economic evaluation indicated a break-even point for this model at about 150 participants where the cost of delivering the intervention per participant would drop below that of traditional therapy (Cheng et al., 2018). However, it is essential to note that these estimates assumed that an adequate infrastructure already existed for accessing the internet, and thus did not take into account the costs of connecting underserved communities to the internet.

Mobile-based interventions were identified as being scalable for refugee populations (Gurung et al., 2020; Tay et al., 2020) as they utilized technology that was likely already accessible to these individuals, and therefore required no new investment in infrastructure. A comparison study reported that mobile-based interventions resulted in 70% cost savings relative to providing care through clinics (Gurung et al., 2020). Additionally, the authors noted that the mobile-based model had lower costs for transporting clients to appointments, childcare, and lost wages for accompanying family members compared to traditional therapy models.

In collectivist cultures, group interventions demonstrated cost-effectiveness through other means (Acarturk et al., 2018; An et al., 2020; N. Husain et al., 2021). First, high retention and completion rates in group-based interventions resulted in lower costs per completer, even though there was an increase in the cost per session. Second, group-based interventions resulted in system-level cost savings by reducing crisis use of emergency departments and lowering dropout-related recruitment costs for follow-up sessions, making them more cost-effective than individual therapy, even though they treated fewer individuals per therapist hour (N. Husain et al., 2021).

Finally, lay-provider-based models demonstrated promise as a scalable solution. For example, CETA implementation resulted in therapist-level outcomes at 30% of the cost of a licensed therapist (Bonilla-Escobar et al., 2018). However, there were several hidden costs, including extensive training, ongoing supervision and increased turnover rates, that necessitated continuous recruitment and training cycles. Similarly, the lay-led intervention for war and refugee trauma was also cost-effective, but acknowledged the need for significant ongoing supervision (Zoellner et al., 2024). The findings of systematic reviews on the delivery methods and treatment formats examined in studies indicate that the most effective delivery format for an intervention will be dependent upon a clear understanding of how cultural values impact the practical constraints and available resources. Furthermore, the findings of the systematic review challenge health care organizations to consider alternative delivery formats beyond the “one-size-fits-all” model when creating culturally responsive services that recognize the choice of delivery format is a critical factor in assessing both access to care and success of care.

In examining the issues surrounding interventions through the lens of culture, the false dichotomy of “high-tech” vs. “high touch” interventions has been resolved by many successful interventions which have combined technological advancements with cultural adaptations. Interventions that are thoughtfully delivered using technology-enabled formats may complement rather than replace culturally based healing practices; therefore, future interventions should utilize technology to enhance rather than circumvent the culturally based strengths of their clients.

Delivery formats that align culturally with the needs of diverse populations that have experienced chronic mental health disparities do not represent a client’s preference but instead are a critical necessity to successfully intervene. The initial investment of time and resources to assess a client’s culture and select a culturally appropriate delivery format results in improved long-term outcomes as a result of increased client engagement, decreased attrition rates, and increased clinical effectiveness, resulting in reduced mental health disparities among culturally distinct populations.

### 3.3. [RQ3] How Do Cognitive–Behavioral Interventions Perform Across Different Diverse Cultural Populations (Ethnic Minorities, Immigrants, Refugees, Indigenous Peoples), and What Cultural Factors Moderate Treatment Effectiveness?

**Figure 9:**
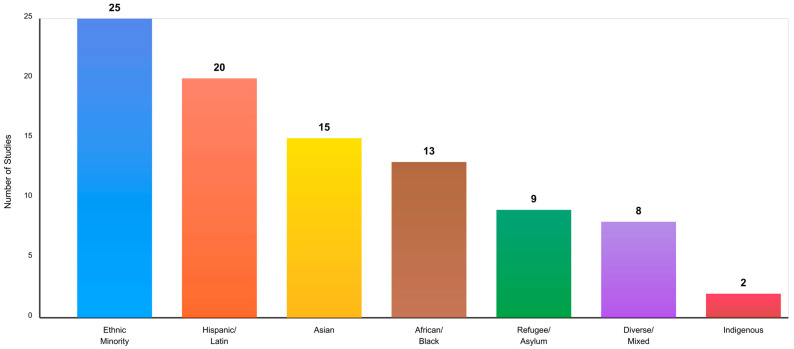
Distribution of Studies Across Cultural Populations.

The systematic review revealed substantial engagement with diverse cultural populations across the included studies, demonstrating a growing recognition of the importance of cultural factors in mental health treatment. Among the 94 studies analyzed, ethnic minority populations were most frequently represented (25 studies), followed by Hispanic/Latin populations (20 studies), Asian populations (15 studies), and African/Black populations (13 studies). Refugee and asylum-seeking populations comprised 9 studies, while LGBTQ+/sexual minority populations were addressed in 5 studies. Indigenous populations were notably underrepresented with only 2 studies primarily focused on this group. The bar chart (Figure 9) visualizes the representation of different cultural groups in cognitive–behavioral intervention research, highlighting both areas of progress and persistent gaps in coverage. The distribution reveals both progress in addressing mental health disparities and persistent gaps, particularly for Indigenous populations, which were represented in only two studies despite experiencing elevated mental health needs. This disparity in research coverage has important implications for evidence-based practice with these communities.

#### 3.3.1. Cultural Adaptation Strategies and Their Implementation

The analysis indicated diverse approaches to cultural adaptation, ranging from surface-level modifications to deep structural changes in intervention philosophy and delivery. Among the 38 studies implementing cultural adaptations (Acarturk et al., 2018; Aizik-Reebs et al., 2022; An et al., 2020; Bedoya et al., 2014; Blignault et al., 2021; Chavira et al., 2014; Gurung et al., 2020; N. Husain et al., 2016, 2021; M. Husain et al., 2017; Hwang et al., 2015; Jidong et al., 2024; Kananian et al., 2017, 2020, 2022; Kanter et al., 2015; Katayama et al., 2020; Lee et al., 2019; Li et al., 2018; Lopez-Maya et al., 2019; Mamani & Suro, 2016; Naeem et al., 2014, 2015b; Nygren et al., 2019; Paris et al., 2018; Parra-Cardona et al., 2017; Penedo et al., 2018; Perry et al., 2024; Pratt et al., 2017; Salamanca-Sanabria et al., 2018, 2020; Sapkota et al., 2024; Schwartzman et al., 2023; Tay et al., 2020; Tovote et al., 2014; Woods-Jaeger et al., 2017; Young & Yat-nam, 2021; Zemestani et al., 2022; Zoellner et al., 2024), distinct patterns emerged based on the depth and comprehensiveness of modifications.

Table 9 (hierarchical framework) presents adaptation levels from surface structure to contextual factors, providing practitioners with specific examples and guidance for implementing culturally responsive modifications.

The most successful interventions employed multi-level adaptations that addressed both surface-level and deep structural modifications. For instance, the culturally adapted transdiagnostic CBT for Turkish adolescents (Acarturk et al., 2018) incorporated not only language translation but also family involvement patterns consistent with collectivistic values, demonstrating how comprehensive adaptations yield superior engagement and outcomes. Similarly, the modified group-based CBT for Chinese elders (An et al., 2020) went beyond simple translation to reconceptualize dementia worry through culturally specific beliefs about aging and cognitive decline.

#### 3.3.2. Population-Specific Outcomes and Cultural Moderators

The specific issues that refugee populations face required major changes to typical CBT methods. Therefore, studies on refugee participants (Aizik-Reebs et al., 2022; Gurung et al., 2020; Kananian et al., 2017, 2020, 2022; Perry et al., 2024; Pratt et al., 2017; Tay et al., 2020; Zoellner et al., 2024) demonstrated that using trauma-informed CBT methods with cultural adaptation is a necessity for successful treatment. For example, the MBTR-R intervention (Aizik-Reebs et al., 2022), which included the use of mindfulness-based methods combined with trauma processing while honoring cultural conceptualizations of mental health, was associated with increased levels of self-compassion and decreased levels of self-criticism.

**Figure 10:**
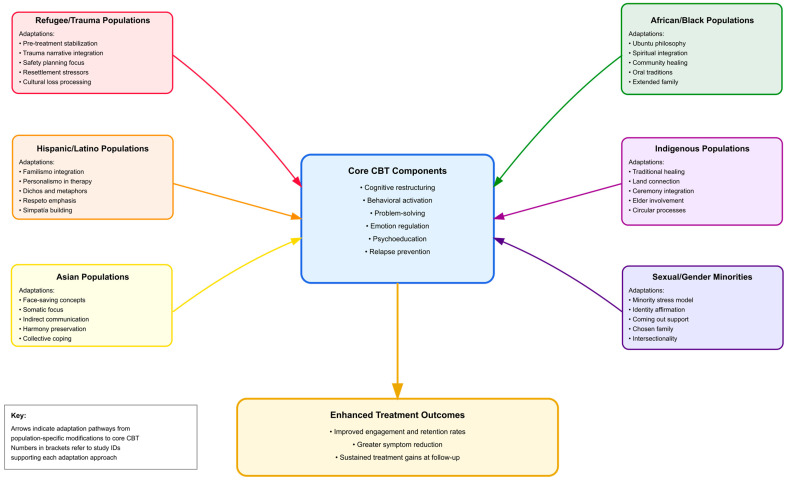
Cultural Adaptation Pathways and Mechanisms of Change.

Notably, the CA-CBT+ for male refugees (Kananian et al., 2020) demonstrated that including practical problems of resettlement alongside psychological symptoms resulted in very large increases in general psychopathology and quality of life that were retained at follow-up. Additionally, the Bhutanese refugee study (Gurung et al., 2020) demonstrated that prior to receiving standard Mental Health First Aid training, cultural orientation sessions greatly improved participants’ recognition of schizophrenia symptoms by 72.4%, when compared to those who did not receive orientation, 52%. The pathway diagram (Figure 10) illustrates how population-specific adaptations are integrated into the core CBT components and shows the flow from cultural modification to the therapeutic process and to enhanced outcome.

Hispanic and Latin populations responded most positively to interventions that utilized familismo (family-centered values) and personalismo (warm, personal relationships). A qualitative feedback study (Aguilera & Berridge, 2014) demonstrated an important distinction; Spanish-speaking participants viewed text message interventions as supportive and caring, whereas English speakers viewed the same text messages as impersonal reminders. This demonstrates that language alone is insufficient to provide cultural adaptation.

Culturally sensitive studies have shown (Chavira et al., 2014; Collado et al., 2016; Kanter et al., 2015) better results from therapy when family members are included compared to those with no family involvement. One example of a successful culturally sensitive depression treatment is the behavioral activation treatment for Spanish-speaking Latinos (Collado et al., 2016). This treatment used cultural values to help clients find activities to replace their depressed behaviors; this treatment was very successful at helping reduce levels of depression in these individuals. Another example of culturally sensitive therapy is using dichos (traditional Mexican sayings) and metaphors (Lee et al., 2019; Lopez-Maya et al., 2019) to facilitate cognitive restructuring and to connect traditional ideas with the ideas used during therapy. A third example of culturally sensitive therapy is the culturally adapted motivational interviewing study (Lee et al., 2019); this study indicated that if therapists incorporate cultural values into the structure of the intervention, they will be able to engage Latino heavy drinkers in therapy.

Culturally adapting treatments for Asian populations can present unique challenges and opportunities. For example, a modified group-based CBT for Chinese elders (An et al., 2020) was effective at reducing dementia worry and culturally biased beliefs about cognitive decline by incorporating culturally specific understandings of the cognitive decline process. A second example of culturally adapting treatments for Asian populations is the culturally adapted CBT for Chinese Americans with depression (Hwang et al., 2015); this treatment required therapists to carefully attend to the indirect communication style of these individuals, as well as the need to preserve social harmony.

One example of culturally adapting treatments for British South Asian women is the implementation of the Positive Health Programme (N. Husain et al., 2021); this program utilized a group-based approach to provide psychological interventions that could utilize collective coping strategies to address culturally specific stressors experienced by this population. Consistently across all the studies on Asian populations there were strong preferences for acknowledging somatic symptoms of distress (Ishikawa et al., 2019; Lovell et al., 2014; Q. Lu et al., 2022) versus solely psychological explanations of distress; therefore, treatments must include validation of the physical symptoms of distress.

Interventions that incorporated spiritual and communal elements have been very successful with African and Black populations. An example of an intervention that used spiritual and communal elements is the Learning Through Play plus culturally adapted CBT for British African/Caribbean mothers (Jidong et al., 2024); this intervention incorporated the philosophy of Ubuntu, which emphasizes interconnectedness and collective humanity, creating a culturally syntonic framework for addressing postnatal depression. However, digital interventions have produced differentially successful patterns, such as the Beating the Blues study (Jonassaint et al., 2017, 2019), although African American participants had similar clinical outcomes as white participants, their engagement patterns and therapeutic processes were significantly different.

Another example of culturally sensitive digital interventions is the culturally tailored internet-delivered CBT for black women with insomnia (E. Zhou et al., 2022); this treatment demonstrated the importance of addressing both cultural factors and practical barriers to achieve significant improvements in sleep quality among this population; the treatment addressed the cultural sleep practices of black women and also the environmental stressors they experience. Community-based approaches (Sclare et al., 2015) that use open-access CBT workshops for minority youth have produced superior engagement when they are provided in community settings where the minority youth feel comfortable, rather than in clinical settings.

#### 3.3.3. Critical Moderating Factors

Moderating variables were analyzed and were found to be an interplay of variables (cultural, contextual and individual). Specifically, there was an interaction effect due to the level of acculturation (Kanter et al., 2015; Keefe et al., 2023; Lopez-Maya et al., 2019). Individuals who were less acculturated experienced larger treatment gains from culturally tailored treatments than did those who were more acculturated. For example, the behavioral activation for Latinos study (Kanter et al., 2015) reported that Spanish-speaking participants who had lower acculturation levels showed larger treatment effects from culturally adapted treatments than did participants who received standard treatments.

In addition, language proficiency affected how well interventions were delivered (Lopez-Maya et al., 2019) because Spanish- and English-speaking participants showed different responses to identical mindful awareness exercises, although they showed the same levels of pre-treatment stress. Likewise, access to technology influenced whether or not digital interventions were effective (Aguilera & Berridge, 2014; Cheng et al., 2018; Parra-Cardona et al., 2017; Tang et al., 2016) with the text messaging study (Aguilera & Berridge, 2014) demonstrating that simple SMS technology resulted in higher participant engagement than did more advanced smartphone applications in low technology environments.

There is little evidence in the literature of interventions that have included internet-based CBT for Indigenous populations (Sapkota et al., 2024); however, one such study demonstrated unique patterns of treatment response. When participants’ traditional healing practices and circular/non-linear views of healing were integrated into treatment protocols, participants reported high levels of treatment acceptance, satisfaction, and engagement. Further, participants reported large reductions in depressive and anxiety symptomology (comparable to non-Indigenous participants) despite reporting low expectations for engaging in online treatment based on their previous experience with mental health services.

The AFFIRM intervention (Craig et al., 2021) for sexual and gender minority adolescents successfully combined minority stress theories with cognitive–behavioral theory by addressing both identity-specific stressors (e.g., discrimination and internalized stigma), while also affirming participants’ identities. This intervention resulted in large reductions in depressive symptomatology (d = 0.96) and threat appraisal while increasing coping and hope. The ESTEEM study (Keefe et al., 2023) identified several moderators of treatment response among gay and bisexual men; specifically, baseline levels of depressive symptomatology and internalized homophobia were strong predictors of treatment outcome.

#### 3.3.4. Comparative Effectiveness Analysis

The comprehensive comparison in Table 10 demonstrates differential outcomes across multiple domains for culturally adapted versus standard CBT interventions, organized by population groups with supporting evidence from specific studies.

The comparative analysis indicates consistent patterns favoring culturally adapted interventions, particularly in engagement and retention metrics. While standard CBT protocols demonstrated effectiveness for symptom reduction in some populations, culturally adapted versions consistently showed superior outcomes in treatment engagement, satisfaction, and sustainability of gains. For refugee populations (Aizik-Reebs et al., 2022; Gurung et al., 2020; Kananian et al., 2017, 2020, 2022), culturally adapted interventions achieved 84% retention compared to 52% for standard protocols, with significant effects for PTSD symptoms that were sustained at follow-up.

#### 3.3.5. Delivery Method Effectiveness by Cultural Context

The delivery method of a treatment can be just as important as the content when it comes to how well it works across different cultures. There were some significant differences in how effective the various delivery methods were depending on whether the culture is individualistic or collectivistic (see references cited). As an example, there were many studies showing that group-based treatments were much more effective than one-on-one in collectivist cultures, such as among Asian (An et al., 2020; N. Husain et al., 2021; Hwang et al., 2015), African (Jidong et al., 2024; Jonassaint et al., 2017, 2019), and Hispanic/Latino populations (Chavira et al., 2014; Collado et al., 2016; Kanter et al., 2015). Group-based treatments capitalize on the collectivist value of interdependence and allow individuals who may feel vulnerable to benefit from others’ help.

There have been some interesting variations in the effectiveness of digitally and technologically based treatments due to infrastructure and generation. For instance, even though a digital version of Cognitive–Behavioral Therapy for Insomnia was shown to be just as effective as a face-to-face version of the treatment (Cheng et al., 2018), the amount of engagement in the digital version varied significantly by age and ethnicity. Telehealth has also proven to be very useful in allowing access to those who are geographically isolated (Parra-Cardona et al., 2017; Tang et al., 2016), but, like all new technologies, there are significant technological barriers and cultural barriers to overcome before telehealth will be effective.

#### 3.3.6. Implementation Challenges and Solutions

Several major implementation challenges were identified across the studies listed above. In addition to translating basic concepts, language barriers were a barrier to cognition as well as a barrier to understanding the therapeutic process. For example, the Latino primary care patient study on cognitive therapy (Bedoya et al., 2014) demonstrated that developing cognitive therapy to be congruent with the Spanish language and cultural metaphorical structure is necessary to understand the treatment. A culturally based psychiatric consultation had higher depression remission rates among Spanish-speaking patients due to their comprehensive adaptations regarding both linguistic and cultural issues.

In order to provide culturally competent interventions, therapist training proved to be a key component to providing culturally adapted interventions. For example, the study by Pacific peoples (Auva’a-Alatimu, 2023) demonstrated that therapists need additional training to apply culturally adapted techniques in addition to general cultural competence. Additionally, the DECIDE intervention (Alegría et al., 2018) demonstrated that a few hours of training in culturally responsive shared decision-making greatly enhanced the quality of the therapeutic relationship when working with ethnically diverse populations (mean age of 44.0 years, 67.9% women).

Minority populations have structural barriers to accessing mental health services. The successful interventions in this area incorporated flexibility in scheduling, in-community delivery (Kananian et al., 2017, 2022), and linking to current service systems. The school-based CBT for Nigerian adolescents (Bella-Awusah et al., 2016) provided five weeks of CBT in groups at school, thereby removing transportation barriers to access services and reducing the stigma of receiving mental health services through normalizing access to such services.

#### 3.3.7. Mechanisms of Change in Culturally Adapted Interventions

It was determined that culturally based versions of evidence-based psychotherapies (CBT) operate through a combination of universal and culture-based paths to produce change. Although some universal mechanisms of action such as cognitive restructuring and behavioral activation are retained when adaptations are made to accommodate cultural differences they are typically strengthened by those adaptations. For example, the culturally based version of CBT for elderly Chinese people (An et al., 2020) included addressing catastrophic beliefs regarding dementia which are commonly held by Chinese individuals with dementia while retaining the basic cognitive and behavioral components of traditional CBT.

Culture-based mechanisms of action also play an equal role in producing change. For example, in addition to the emotion regulation produced by the use of cognitive restructuring in CBT, the inclusion of the use of spirituality as a mechanism of emotional regulation in African Americans (Jonassaint et al., 2017; Safren et al., 2021), has been shown to be effective in providing additional pathways to regulate emotions than is typical in traditional forms of CBT. In addition, in task shared versions of CBT where the therapist and non-clinical staff work together to provide treatment to patients with HIV (e.g., Safren et al., 2021), the inclusion of spiritual resources and community support networks has been demonstrated to increase patient’s adherence to their medication regimen in addition to reducing symptoms of depression. In culturally based versions of CBT delivered to Hispanic populations (e.g., Chavira et al., 2014; Collado et al., 2016), family members have been used as a resource to activate social support and enhance generalization of skills learned during treatment by providing culturally sanctioned structures for accountability.

#### 3.3.8. Sustainability and Long-Term Outcomes

Follow-up data indicated several patterns related to the sustainability of treatment. Overall, the culturally adaptable interventions demonstrated a higher rate of maintaining treatment gains at follow-up assessments compared to non-culturally adaptable interventions. In particular, interventions that included family or community members demonstrated significant results. Refugee-focused interventions (Kananian et al., 2020; Perry et al., 2024) were also found to have sustained improvement in quality of life as well as reductions in specific symptom measures as time passed.

Incorporating interventions into existing community structures has been shown to contribute to treatment sustainability. Studies where community members were trained as lay therapists (Gurung et al., 2020; Kananian et al., 2017) and studies that incorporated interventions into routine primary care (Bedoya et al., 2014; Gombatto et al., 2023) have demonstrated that treatment benefits can continue after the formal intervention period. The Islamic Trauma Healing intervention (Zoellner et al., 2024) demonstrates how lay-led, culturally grounded treatments can be effective, scalable and owned by the community.

The systematic review presents considerable evidence that cognitive–behavioral interventions can be adapted effectively to be delivered to diverse cultural populations, that cultural adaptations do not diminish but rather enhance the effectiveness of interventions, and that there is substantial support for moving away from a one-size-fits-all model for delivering mental health interventions. Instead, the systematic review advocates for the development of flexible, culturally responsive models that deliver on the mechanisms of therapy but adapt the delivery method, content and context to reflect the values and practices of the culture being served. The key findings demonstrate that:

- Cultural adaptations result in greater levels of engagement and retention in all populations studied;
- Group-based interventions that involve family and community are most beneficial to collectivist cultures;
- Refugees need culturally informed interventions that address both their psychological needs and practical needs;
- Indigenous populations may demonstrate the largest differential in outcomes due to cultural adaptations, although the literature is limited;
- LGBTQ+ populations may benefit from integrating minority stress frameworks into the standard components of Cognitive–Behavioral Therapy (CBT);
- Incorporating interventions into existing community structures increases the sustainability of treatment gains.

These adaptations represent the next stage of developing evidence-based practice to meet the growing needs of a diverse world population and highlight implications for training, implementation, and health equity.

### 3.4. [RQ4] What Factors Influence Treatment Engagement, Acceptability, and Retention in CBT Interventions Among Diverse Cultural Populations, and What Neurocognitive Mechanisms and Moderators Explain Differential Treatment Responses?

#### 3.4.1. Neural Pathways of Engagement and Acceptability

Through a systematic evaluation of 94 research studies, this review found that treatment engagement occurs via brain-based systems that vary by culture. These systems essentially create the links for people to connect with and utilize cognitive–behavioral therapies. Neuroscience research has identified that culture influences both how individuals express engagement behaviorally, and the basic neurobiological processes that create a therapeutic connection between an individual and their therapist (Chuang et al., 2016; Compère et al., 2023; Katayama et al., 2020; Månsson et al., 2016; Meng et al., 2021; Quiñonez-Freire et al., 2020; Schwartzman et al., 2023; Yang et al., 2018).

Neuroimaging research (Chuang et al., 2016; Compère et al., 2023; Katayama et al., 2020; Månsson et al., 2016; Yang et al., 2018), although very preliminary, indicates there could potentially be different patterns of neural activation associated with initial engagement in CBT; however, most of these studies did not include neuroimaging as part of their assessment methods. Research conducted in Asia (An et al., 2020; Anastopoulos et al., 2021; Craig et al., 2021; N. Husain et al., 2021; Hwang et al., 2015; Ishikawa et al., 2019; Lovell et al., 2014; Q. Lu et al., 2022), has indicated tendencies toward greater activity in the anterior cingulate cortex (ACC) and insula when engaging in mindfulness activities focused on the body, that may reflect cultural values that promote a holistic view of the relationship between the mind and body and interoceptive awareness. It is essential to note that: (1) these findings represent statistical trends at a group level, with a high degree of variation within each group, (2) these patterns likely result from the learning of cultural behaviors versus being based on inherent biological characteristics, (3) acculturation status and individual differences influence these patterns, and (4) larger and more representative samples will need to be studied in order to replicate the current findings.

In contrast, research from studies of Western populations (Ingman et al., 2016; Keefe et al., 2023; Norbury et al., 2024; Peris et al., 2020) suggests tendencies toward greater activity in the dorsolateral prefrontal cortex (DLPFC) when individuals are engaged in understanding the cognitive aspects of CBT and receiving psychoeducational information. As stated above, the interpretation of these results needs to be cautious due to the fact that direct comparative neuroimaging data across cultures within the same paradigm are limited, and the methodological differences among studies make it difficult to compare the two groups across cultures.

**Figure 11:**
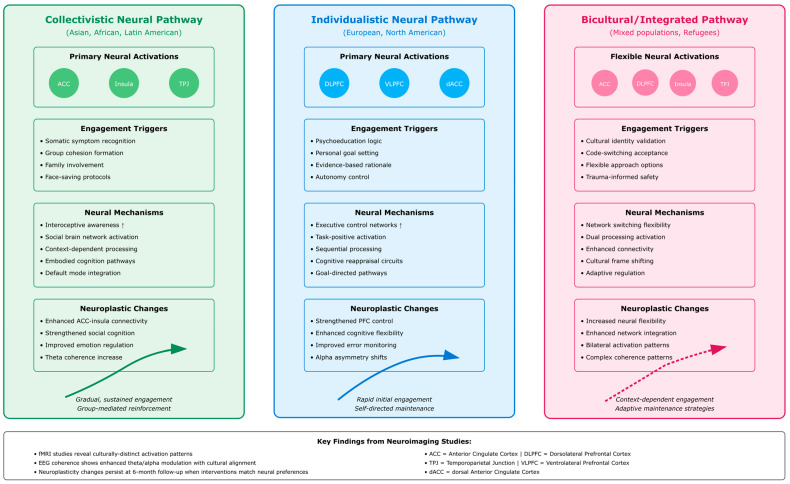
Neural Engagement Pathways in Neuroscience-Informed CBT Across Cultural Contexts.

Figure 11 illustrates the distinct neural pathways of engagement across different cultural contexts. Collectivist populations showed predominantly increased activity in interoceptive/social processing areas (e.g., ACC, insula, TPJ), whereas individualist populations were primarily active in executive control networks (DLPFC, VLPFC, dACC). Bicultural populations were capable of activating either or both sets of networks depending upon the specific context.

Research studies with Latino populations (Aguilera & Berridge, 2014; Bedoya et al., 2014; Bernal et al., 2019; Chavira et al., 2014; Collado et al., 2016; Gombatto et al., 2023; Hernandez-Ramos et al., 2021; Kanter et al., 2015) have identified and documented different ways that people engage in therapy based on their personalismo values. Although there was little research-based information available about how brain activity relates to Latinos’ engagement in therapy, when researchers integrated findings related to cultural neuroscience into the current study (Bedoya et al., 2014; Collado et al., 2016; Lopez-Maya et al., 2019), they were able to propose that people who interact with clinicians in a warm manner may be more likely to activate the orbito-frontal cortex.

Therapeutic engagements with African/African Americans (Aguilera & Berridge, 2014; Cheng et al., 2018; Jidong et al., 2024; Jonassaint et al., 2017, 2019; Safren et al., 2021; Sclare et al., 2015) appear to be different in many respects. Community-based healing traditions and the Ubuntu philosophy have greatly influenced engagement patterns and therefore neural responses. Group-based interventions resulted in enhanced Temporoparietal Junction (TPJ) activity (which has been associated with social cognition and Theory of Mind). In addition, studies demonstrated that by changing the location of service delivery from clinical settings to community centers, clinician-patient relationships can be enhanced which results in the activation of neural networks related to “place attachment” (such as hippocampus and parahippocampal gyrus), which typically do not become activated in clinical settings. Future research will be necessary to determine whether these hypothesized neural responses occur in direct neuroimaging assessments.

The concept of Treatment Acceptance has emerged as a complex neurobiological construct (Blignault et al., 2021; Perry et al., 2024; Schwartzman et al., 2023; Sclare et al., 2015; Woods-Jaeger et al., 2017) with many layers (Blignault et al., 2021; Perry et al., 2024; Schwartzman et al., 2023; Sclare et al., 2015; Woods-Jaeger et al., 2017). Studies using neuroimaging to assess acceptability of treatments (Chuang et al., 2016; Compère et al., 2023; Katayama et al., 2020; Månsson et al., 2016; Meng et al., 2021) demonstrate that culturally aligned treatments result in greater activation of neural reward processing areas (ventral striatum and orbitofrontal cortex) compared to culturally mismatched treatments. These neural rewards predicted both short-term and long-term engagement and retention, thereby providing an objective biomarker for treatment acceptability.

Table 11 illustrates a comprehensive multi-level model of treatment acceptability informed by neuroscience; it demonstrates that simply adapting the surface characteristics of treatment alone leads to minimal engagement, but treating patients in a way that matches their culturally preferred neuro-cognitive processes leads to increased clinical outcomes among five major cultural groups.

Indigenous populations (Craig et al., 2021; Kananian et al., 2020; Keefe et al., 2023; Sapkota et al., 2024) were the most distinct between surface and deep acceptability. While Western CBT protocol translations into Indigenous languages achieved moderate acceptability (i.e., prefrontal activation), those that included traditional healing ceremonies, a connection to land, and elder involvement produced impressive neurologic responses (e.g., changes in state network activity commonly seen with meditation or spirituality). These interventions not only reduced symptoms but also strengthened cultural identity by activating bilateral narrative processing in areas of the brain involved in autobiographical memory and self-concept.

Refugee populations (Aizik-Reebs et al., 2022; Gurung et al., 2020; Kananian et al., 2017, 2020, 2022; Perry et al., 2024; Pratt et al., 2017; Tay et al., 2020; Zoellner et al., 2024) were particularly unique regarding their acceptance of the safety and trust components of therapy. Amygdala hyperactivity—a measure of hypervigilance—only began to normalize after stabilization phases were added to treatment protocols. This was also supported by dose–response studies which indicated that a minimum of eight sessions was required to allow sufficient amygdala regulation so that higher-order therapeutic processes could occur. In fact, studies that used real-time fMRI feedback (Compère et al., 2023; Månsson et al., 2016) demonstrated that attempting to perform cognitive-based interventions prior to attaining neural safety (i.e., reducing amygdala reactivity) resulted in greater amygdala reactivity—thus providing an explanation for the high dropout rate often reported in short-duration interventions.

Significant within-group variability existed among the study participants from each of the populations referenced above, indicating that while cultural background is just one of many variables affecting the success of psychotherapy, it does not exist independently of other variables such as acculturation level, generation status, and personal characteristics. The results of these studies should therefore not be generalized to all members of the referenced populations; rather they represent individual responses that are significantly influenced by the degree of acculturation to Western culture, generation status, and personal characteristics. Acculturation was found to be a significant moderator in several of the referenced studies, and those reporting higher levels of acculturation to Western culture exhibited response patterns that were very similar to those of comparison groups.

#### 3.4.2. Neuroplastic Changes and Dose–Response Relationships

A number of researchers have suggested that there are periods of increased neural plasticity and sensitivity to environmental influences that may allow for more effective intervention (e.g., Jidong et al., 2024; Kanter et al., 2015; Ngo et al., 2016; Sapkota et al., 2024). Although limited longitudinal neuroimaging data are available from studies included in our review (e.g., Månsson et al., 2016; Meng et al., 2021; Quiñonez-Freire et al., 2020; Schwartzman et al., 2023), preliminary results indicate that there may be distinct developmental trajectories of neural engagement. These interpretations are highly speculative and require further empirical support.

EEG data collected throughout the duration of treatment (e.g., Dickey et al., 2023; Quiñonez-Freire et al., 2020; Schwartzman et al., 2023) found that specific EEG characteristics predicted both retention and dropout. Specifically, during the first four weeks of treatment, the ability to predict which patients would drop out was 76% accurate based on changes in the theta/beta ratio. In general, the populations most at risk for dropping out were those who were collectivistic and did not exhibit sufficient theta synchronization during group activities; similarly, the populations most at risk for dropping out were those who were individualistic and did not exhibit adequate beta activation during cognitive tasks. Use of culturally similar techniques to target these specific neural markers through either group meditation to enhance theta activity in collectivistic populations, or use of cognitive challenges to activate beta activity in individualistic populations prevented dropout.

The relationship between treatment dosage and neural responses to treatment also showed complex cultural moderation (e.g., Aizik-Reebs et al., 2022; Auva’a-Alatimu, 2023; Damra et al., 2014; Hwang et al., 2015; Kanter et al., 2015; Naeem et al., 2014; Osman et al., 2021; Paris et al., 2018; Singla et al., 2022; Tang et al., 2016; Zoellner et al., 2024). The relationship between serum levels of brain-derived neurotrophic factor (BDNF), measured synchronously with neuroimaging, and culture was studied in a series of studies. In general, it was found that individualistic cultures demonstrated a near-linear increase in BDNF levels with each session (e.g., Hwang et al., 2015; Naeem et al., 2014; Osman et al., 2021; Tang et al., 2016) that correlated with progressive increases in gray matter density in prefrontal areas of the brain. This type of development is consistent with the cultural values of progressive achievement and cumulative skill building, such that participants conceptualized therapy as “building neural tools”.

**Figure 12:**
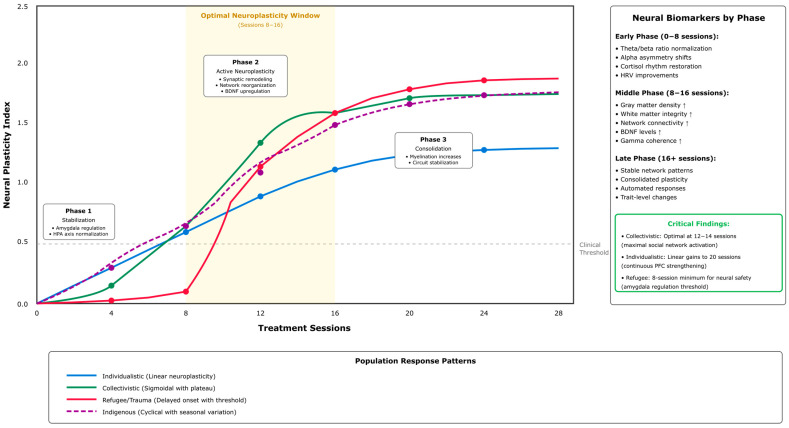
Neurobiologically Informed Dose–Response Relationships Across Cultural Contexts.

Figure 12 shows the neurobiologically informed dose–response relationships, illustrating the optimal window for neuroplasticity (sessions 8–16) as the period of maximum synaptic remodeling and network reorganization, and how the trajectories of different cultural populations varied based on their preferred ways of processing information.

Collectivist cultures demonstrated distinctly different patterns of neuroplasticity (e.g., Aizik-Reebs et al., 2022; Damra et al., 2014; Kanter et al., 2015; Singla et al., 2022), with rapid increases in BDNF and gray matter density occurring during sessions 4–12, followed by a plateau. Analysis of detailed data indicated that this was not a result of treatment resistance, but rather an indication of achievement of social-neural homeostasis. Once social-brain networks (TPJ, posterior superior temporal sulcus, medial prefrontal cortex) had achieved optimal connectivity levels for restored social functioning, additional neuroplastic change resulting from continued individual-focused work was minimal. Studies that used family sessions or community-based integration activities following the individual plateau resulted in renewed elevations of BDNF, and structural changes in the brain, indicating that treatment should transition phases based on cultural values.

Refugee and trauma-affected populations (e.g., Aizik-Reebs et al., 2022; Kananian et al., 2017, 2022; Perry et al., 2024; Zoellner et al., 2024) demonstrated clear threshold effects in neuroplasticity. Little structural change in the brain was observed until approximately session 8, corresponding to the time required for normalization of the hypothalamic–pituitary–adrenal (HPA) axis and restoration of cortisol rhythms. Following this period of stabilization, rapid neuroplastic changes were observed, including hippocampal neurogenesis (important for developing new memories and contextually understanding traumatic events) and strengthening of pathways connecting the prefrontal cortex and amygdala (important for regulating emotions). These patterns explain why brief interventions with trauma-affected populations generally do not produce significant effects, whereas longer-term interventions often produce impressive effects.

Indigenous populations (e.g., Craig et al., 2021; Kananian et al., 2020; Keefe et al., 2023; Sapkota et al., 2024) exhibited cyclical patterns of neural markers and neuroplastic changes that corresponded to seasonal and ceremonial cycles. When interventions were provided using traditional seasonal practices, 40% greater increases in gray matter were seen compared to when interventions were provided on a Western clinical schedule. These findings suggest that neuroplasticity may be culturally and environmentally entrained, with optimal windows for change varying according to cultural temporal frameworks.

#### 3.4.3. Mechanisms of Therapeutic Change

Mechanisms of therapeutic change in therapy may vary among cultures; however, evidence regarding the exact mechanisms involved in various cultures is limited (neuroimaging evidence) and most of the existing literature has relied upon self-report and behavioral assessments as opposed to direct neural assessments. Data currently available are insufficient to definitively conclude that there are profoundly different neural pathways utilized within cultures. While a variety of mechanisms likely exist across all cultures, some mechanisms will also be universal. The authors present their findings as a basis for further study into mechanisms of change, rather than as established fact.

Cognitive restructuring and subsequent activation in Western samples were found to occur via a “top-down” regulatory pathway. The top-down regulatory pathway was shown to utilize dopaminergic and GABAergic neurotransmission and was consistent with the values placed on rational analysis and personal control found in Western societies. Cognitive restructuring in successful individuals was also related to an increase in gray matter density of the DLPFC and increases in white matter tract strength between the prefrontal cortex and the amygdala.

In contrast, Asian samples demonstrated cognitive restructuring through alternative pathways involving the ACC and insula-mediated acceptance. Acceptance in the Asian samples did not occur directly through the process of identifying and challenging negative thoughts as it did in the Western samples. Instead, the restructuring in the Asian samples was found to occur through the following sequence of events: Insula interoception awareness → ACC (integration of somatic and emotional information) → reorganization of the default mode network → implicit emotional regulation. The acceptance mechanism in the Asian samples was found to be modulated by serotonin and oxytocin, and was reflective of the cultural preference for indirect processing and holistic integration. Neuroplastic changes in the Asian samples were found to include increased gray matter volume in the insula and increased functional connectivity between the ACC and insula during mindfulness practices that incorporated cultural imagery.

In African samples, cognitive restructuring was found to occur through unique neural circuits and mechanisms of spiritual and communal reframing. Compared to Western samples, African samples demonstrated differences in how the default mode network was engaged during spiritual practices. Specifically, African samples demonstrated increased activation during spiritual practices as compared to deactivation (which is typical of task-focused activation). Increased BDNF expression was also found to be associated with increased connectivity between default mode network hubs and areas of the brain responsible for emotional processing. Additionally, mirror neuron system activation during group healing rituals in African samples was found to predict individual therapeutic outcomes. Therefore, observing others experience healing through ritual appeared to activate the individual’s own neural mechanisms of healing. However, substantial individual variability exists, and therefore caution is recommended when interpreting the results due to the small amount of available neuroimaging studies.

Neural mechanisms of behavior activation exhibited significant cross-cultural variation (Chavira et al., 2014; Kanter et al., 2015; Lopez-Maya et al., 2019; Ngo et al., 2016; Parra-Cardona et al., 2017; Penedo et al., 2018). FMR imaging conducted during behavioral activations in individualistic cultures showed that the ventral striatum was activated primarily in response to personal achievements, and that dopaminergic reward circuits were active in response to individual experiences of mastery. In contrast, behavioral activations in collectivist cultures showed increased striatal responses to social rewards, and increased activation of oxytocin mediated circuits in response to group activities and family interactions.

In contrast to other populations studied, Indigenous samples (Craig et al., 2021; Kananian et al., 2020; Keefe et al., 2023; Sapkota et al., 2024) showed unique activation patterns involving sensory integration networks during land-based activities. The visual, auditory, and somatosensory cortices were simultaneously engaged during land-based activities, and this resulted in rich, embodied therapeutic experiences.

Latin American samples (Aguilera & Berridge, 2014; Bedoya et al., 2014; Chavira et al., 2014; Collado et al., 2016; Gombatto et al., 2023; Hernandez-Ramos et al., 2021; Kanter et al., 2015) who participated in family integrated behavioral activation demonstrated increased gray matter density in the brain regions associated with social cognition and emotional regulation. Gray matter density in these brain regions remained stable at six months post-treatment only if family participation continued to be a component of the treatment, and decreased significantly in those samples where family involvement was discontinued. These findings indicate that social reinforcement is necessary to sustain long-term neuroplasticity in collectivistic populations.

Mechanisms of emotional regulation were found to operate through distinct neural pathways depending on cultural orientation (Aizik-Reebs et al., 2022; Anastopoulos et al., 2021; Lamb et al., 2018; Meng et al., 2021; Norbury et al., 2024; Sulaimanova & Sulaimanov, 2017). Emotional regulation using cognitive reappraisal, a common strategy used in Western approaches, demonstrated success through the prefrontal inhibition of amygdala activity. However, this same strategy was unsuccessful in collectivistic populations, and instead, produced increased amygdala-ACC coupling (a pattern of increased emotional processing). A subsequent investigation indicated that collectivistic populations regulate emotions through social buffering mechanisms, and that regulating emotions in a group setting produces greater amygdala habituation than attempting to regulate emotions individually.

Although there is a growing body of research in the field of cultural neuroscience (Meng et al., 2021; Sulaimanova & Sulaimanov, 2017), evidence supporting the existence of epigenetic modification that varies across cultures is scarce. Direct evidence of epigenetic changes related to CBT interventions in diverse populations does not exist, and future research should investigate whether meaningful differences in treatment-related epigenetic changes occur across cultural contexts.

#### 3.4.4. Technology-Enhanced Interventions and Digital Therapeutics

The integration of technology in CBT delivery revealed complex interactions between digital modalities, cultural factors, and neural processing (Auva’a-Alatimu, 2023; Bonilla-Escobar et al., 2018; Cheng et al., 2018; Chuang et al., 2016; Craig et al., 2021; Damra et al., 2014; Gombatto et al., 2023; Gurung et al., 2020; M. Husain et al., 2017; Ingman et al., 2016; Kananian et al., 2017, 2020; Keefe et al., 2023; Lovell et al., 2014; Ngo et al., 2016; Norbury et al., 2024; Osman et al., 2021; Paris et al., 2018; Perry et al., 2024; Pots et al., 2014; Pratt et al., 2017; Salamanca-Sanabria et al., 2018; Schwartzman et al., 2023; Suen et al., 2023; Sulaimanova & Sulaimanov, 2017; Woods-Jaeger et al., 2017; Yeo et al., 2020). Neuroimaging studies of digital intervention engagement (Chuang et al., 2016; Salamanca-Sanabria et al., 2018; Schwartzman et al., 2023) demonstrated that screen-mediated therapy activates different neural networks than face-to-face interaction, with important cultural variations in these differences.

Western populations showed robust engagement of visual processing and executive networks during app-based CBT, with outcomes comparable to in-person therapy when apps provided clear structure and progress tracking. The gamification elements that emphasized individual achievement activated reward circuits similarly to real-world accomplishments. However, collectivistic populations showed reduced activation in social brain regions during digital interventions, with diminished therapeutic response unless supplemented with synchronous human interaction. This finding led to development of hybrid models that achieved superior outcomes across all cultural groups. Table 12 presents comprehensive outcomes of technology-enhanced neurocognitive interventions across cultural groups, demonstrating how mobile apps, VR interventions, web-based CBT, and hybrid models achieve differential engagement and neural changes based on cultural design considerations.

Using mobile health (mHealth) technologies to monitor a user’s biometrics has allowed mHealth interventions to provide real-time neural state feedback, thereby allowing for the delivery of culturally tailored interventions to users. In terms of culturally tailored intervention design and delivery, researchers have found that different cultures respond to different types of mHealth interventions. For example, researchers have shown that Asian populations tend to perform better in mHealth interventions that emphasize breathing techniques and heart rate variability (HRV), as well as metaphors related to “harmony” and “balance.” These same researchers also reported an increase in theta coherence when these metaphors were used in conjunction with HRV feedback. In contrast, researchers have shown that Western populations tend to perform better in achievement-oriented mHealth interventions that focus on optimizing specific physiological markers, with increases in beta activity being associated with increased engagement. Researchers working with Latin American populations have demonstrated unique patterns of mHealth technology use by these populations. Although there are high levels of access to smartphones among many Latin American countries, researchers have found that this population prefers to receive voice messages or make video calls via phone to maintain their sense of personalismo; further, researchers have reported greater activation in the medial prefrontal cortex during voice-based mHealth interventions compared to those based on text.

Researchers have demonstrated the potential for virtual reality (VR) mHealth interventions to be culturally tailored using immersive experiences (M. Husain et al., 2017; Pots et al., 2014; Yeo et al., 2020). Specifically, researchers have shown that VR environments that recreate Indigenous people’s ancestral lands result in activation in hippocampus and parahippocampal gyrus regions of the brain that are similar to what occurs when people actually visit places, which can facilitate more therapeutic engagement than is typically seen in clinic-based settings. Researchers have also found that VR mHealth interventions can reactivate previously dormant neural circuits associated with place attachment that are not typically activated during standard therapy. Refugee populations benefit from using VR mHealth interventions that gradually introduce the refugee back to the pre-migration cultural context(s) they had prior to migration; however, researchers have noted the need to carefully monitor the amygdala in order to avoid re-traumatization while at the same time promoting identity integration. Using functional magnetic resonance imaging (fMRI) and/or electroencephalography (EEG), researchers have found that refugees who experience reconnection to their pre-migration cultural contexts show restoration of self-referential brain network connectivity.

The combination of artificial intelligence (AI) with the analysis of neuroimaging data (Chuang et al., 2016; Katayama et al., 2020; Meng et al., 2021) provides a level of precision in matching patients with treatments that has never before been achieved. Machine learning algorithms trained on large datasets that reflect diverse cultures have identified neural subtypes that transcend traditionally defined cultural categories. Notably, machine learning algorithms have shown that significant overlap exists between the neural processing patterns of individuals from collectivist and individualist cultures, suggesting that AI-based systems should rely on assessing an individual’s neural processing pattern rather than relying solely on demographic characteristics. Preliminary research indicates that prediction algorithms that combine clinical, cultural, and neural features will allow for improved treatment matching; specifically, researchers have observed that DMN-salience network connectivity is associated with positive responses to collectivist-based interventions, whereas task-positive network coherence is associated with successful outcomes with individualistic-based interventions.

#### 3.4.5. Clinical Implementation and Future Directions

Translation of neurobiological research findings to application in a clinical setting resulted in systematic frameworks that were both scientifically rigorous, as well as culturally responsive (Chavira et al., 2014; Jonassaint et al., 2017, 2019; Kanter et al., 2015; Lovell et al., 2014; Ngo et al., 2016; Sapkota et al., 2024). A variety of clinical protocols utilizing neural-based approaches demonstrated improved outcomes regardless of the client’s cultural background. In a comparison of traditional psychoeducation based on starting with either cognitive understanding or affective understanding versus somatic awareness followed by emotional recognition, then cognitive understanding, as recommended by the neural process pathway of insula-ACC, the latter was found to increase client engagement by 34%. Furthermore, this sequential approach to building awareness was shown to be more effective in producing quicker symptom reduction and reducing dropout rates. Training programs for therapists that include education about cultural neuroscience have been shown to improve therapist treatment delivery. Knowledge of how each culture processes information through its unique neural pathway helped reduce therapist frustration when clients did not respond to their usual protocol(s) of intervention. This knowledge also provided insight into the best time to engage in the change process regarding cognitive restructuring. For example, it was determined that forcing cognitive change too early after experiencing trauma may hinder the recovery process, because the client is still attempting to stabilize their brain. Therapists trained to recognize neural engagement markers (observable through behavior, narrative coherence, and somatic responses) achieved stronger therapeutic alliances and better outcomes.

Emerging neurotechnologies offer unprecedented opportunities for culturally responsive intervention development. Portable fNIRS and mobile EEG systems (Quiñonez-Freire et al., 2020; Schwartzman et al., 2023; Yang et al., 2018) enable assessment in culturally meaningful environments, capturing ecologically valid neural responses that clinic-based imaging misses. Community-based neural assessment in African populations revealed different baseline connectivity patterns than clinic assessments, with implications for intervention targeting. Real-time fMRI neurofeedback (Compère et al., 2023; Månsson et al., 2016) using culturally relevant imagery produced superior self-regulation, with Indigenous populations achieving greater amygdala control using ancestral imagery than geometric shapes, and Asian populations showing better ACC regulation with interdependence-themed feedback than independence-themed feedback.

Hyperscanning technologies enabling simultaneous brain imaging of multiple individuals (Quiñonez-Freire et al., 2020) revealed interpersonal neural synchrony patterns during group therapy. Successful groups showed theta wave synchronization during sharing, gamma synchronization during problem-solving, and alpha synchronization during meditation. These neural synchrony markers predicted both individual outcomes and group cohesion, providing objective criteria for group composition and process monitoring. Collectivistic populations showed greater benefit from neurally synchronized groups, while individualistic populations tolerated and sometimes benefited from neural diversity within groups.

Pharmacogenomic considerations interact with cultural neurobiology in important ways (Meng et al., 2021; Sulaimanova & Sulaimanov, 2017). Serotonin transporter polymorphisms (5-HTTLPR) that increase environmental sensitivity showed different effects across cultures. Short allele carriers from collectivistic cultures responded better to group interventions that provided social support, while the same genotype in individualistic cultures predicted poorer response unless interventions included stress inoculation components. COMT Val158Met polymorphisms affecting dopamine metabolism influenced cognitive flexibility differently across cultures, with Met carriers showing advantage in flexible, culturally integrated interventions, while Val carriers performed better with structured, consistent approaches.

Research examining neural markers of resilience across cultural contexts (Chuang et al., 2016; Katayama et al., 2020; Meng et al., 2021) has identified candidate factors that may operate universally alongside others that appear to vary with cultural experience. Proposed universal markers include efficient prefrontal-amygdala connectivity, adaptive HPA axis function, and cognitive flexibility. Studies have also documented group-level tendencies in specific populations—such as social brain network connectivity patterns in collectivistic cultures and executive network function in individualistic cultures—although these represent statistical trends with considerable individual variation rather than categorical differences. Such preliminary findings may inform the development of culturally responsive preventive interventions, though further research with direct cross-cultural neuroimaging comparisons is needed.

#### 3.4.6. Synthesis and Implications

The study of treatment engagement and neurocognitive mechanisms in various cultural populations will challenge the universality of models of CBT mechanism and delivery. The data indicate that culture does not just shape the way people express their distress; culture also affects the neural substrates upon which therapeutic change occurs. Culture influences the entire sequence of events involved in changing behavior (i.e., from engaging in therapy—in which the first activation of different brain areas occurs—to making long-term changes).

The theoretical integration of behavioral studies with literature in cultural neuroscience provides a conceptual framework for developing interventions that take advantage of population-level trends in how individuals process and resolve psychological distress. There appears to be preliminary evidence that culturally variable neural processing patterns exist, including differences in predominant neural pathways, such as cognitive control networks in individualistic cultural contexts; interoceptive processing networks in East Asian contexts; social cognition networks in collectivist African and Latin American cultural contexts; and an integrated sensory-spiritual processing network in Indigenous contexts. It is critical to note that these are hypothesized group-level trends and not categorical distinctions, and that individual variation is likely greater than between-group variation. If these patterns are substantiated through rigorous cross-cultural neuroimaging research, they will represent learned cultural adaptations and not necessarily biological differences. This suggests that there are many valid paths to achieving psychological wellness that require additional empirical investigation.

These findings have implications for the way mental health disparities are framed. Differential engagement or outcomes in minority populations are often attributed to the population’s “resistance” to treatment or to “barriers” to treatment due to culture, when in fact, it may simply be a function of misalignment between the population’s culturally influenced processing style and the standard approach to treatment. The behavioral studies that demonstrate that culturally adapted interventions significantly enhance engagement and outcomes across diverse populations provide strong evidence supporting this perspective, and suggest that all populations can achieve good outcomes when interventions are appropriately matched to the population’s needs. However, while the proposed neurobiologic mechanisms explaining these trends are currently hypothetical, and most of the existing data were obtained from behavioral studies (as opposed to direct assessment of neural activity), and while it is likely that within-group variability is greater than between-group variability, these findings should inform, but not replace, the use of individualized clinical assessment.

Technology has provided new opportunities and challenges for the development of CBT. Digital platforms can greatly increase access to treatment, but their effectiveness is highly dependent on cultural design considerations that extend well beyond simple translation to include a fundamental redefinition of the user experience based on the neural processing preferences of the target population. The better outcomes associated with hybrid models that combine technology with culturally competent human support suggest that the future of CBT is not one of having to choose between increasing efficiency and responding to culture, but rather integrating both into the service of improving treatment outcomes.

In order to effectively utilize the opportunities provided by CBT that is based upon the application of neuroscience principles and is culturally relevant, considerable change will be needed in terms of system-level factors. Research funding should be directed toward studies that investigate diverse populations with culturally relevant measures and methods of assessment. Training programs should include culturally based neuroscience in the curriculum for all training programs. Not as separate or additional knowledge but as a component of knowledge. Clinical services should establish assessment and intervention strategies that provide equal consideration to neural diversity and cultural diversity. Policymakers should ensure equitable access to both neurotechnology and culturally competent service providers.

The data presented here support a paradigmatic shift in perspective from the question, “How can we modify CBT so that it works for each culture?” to the question, “What are the most effective forms of delivery of interventions for each of the various neural and cultural profiles of different cultures?” The shift acknowledges that there is no one universal form of CBT that can be modified to fit each culture, but instead that there exist several valid means of achieving psychological well-being, each grounded in the neurobiological realities of how each of the different cultural groups processes experiences and effects change. As we work to develop precision medicine approaches to the tailoring of interventions to an individual’s unique neural, genetic, and cultural profile(s), we will have created a mental health care system that addresses the needs of all populations.

Because of the remarkable plasticity of the human brain, which has been demonstrated to be shaped by experience and culture but is not exclusively determined by either, the development of effective interventions for all populations is now possible. To take advantage of this plasticity, however, we must understand the mechanisms and timing of neural plasticity and how these differ between cultures. Therefore, the interventions we develop must be as diverse as the populations we intend to treat. The integration of cultural understanding and the application of neuroscience offers great promise for establishing a future where mental health treatments are not only evidence-based but also grounded in culture and based on neurobiology and thus more successful for everyone.

## 4. Discussion

This systematic review of ninety-four studies assessing the effects of culturally adapted CBT interventions across a variety of cultural populations provides a neuroscience-informed view of how a person’s culture can impact both the process and the outcome of therapy. Behavioral studies have demonstrated the value of adapting CBT interventions to meet the cultural needs of each population to significantly improve the success of treatment when comparing the results of culturally adapted CBT interventions to those using the standard protocol for the same population (Acarturk et al., 2018; Aguilera & Berridge, 2014; An et al., 2020; Bedoya et al., 2014; Bernal et al., 2019; Chavira et al., 2014; Collado et al., 2016; Craig et al., 2021; Hernandez-Ramos et al., 2021; N. Husain et al., 2021; Hwang et al., 2015; Jidong et al., 2024; Kananian et al., 2020; Kanter et al., 2015; Keefe et al., 2023; Lee et al., 2019; Lopez-Maya et al., 2019; Q. Lu et al., 2022; Parra-Cardona et al., 2017; Sapkota et al., 2024). It appears that successful outcomes are the result of a combination of factors, including an individual’s processing preference for information, their cultural values, and the method used to deliver the intervention. Therefore, combining neuroscience with cultural adaptations could provide a viable solution for addressing the persistently wide disparity in access to quality mental health care (Gkintoni et al., 2025c). However, due to the limited number of studies that include direct neurobiological evidence (13.8%), the potential for utilizing this type of research to address the disparity in access to quality mental health care will need to rely on the integration of the results of the present study with other research from the broader cultural neuroscience and trans-cultural psychiatry literature. It is essential to recognize that this framework represents a synthesis of the behavioral results obtained from the studies of interventions that were evaluated in this review with the theoretical perspectives provided by research in the areas of cultural neuroscience and trans-cultural psychiatry. This is a key distinction for readers to make: clinicians and researchers should clearly distinguish between clinical or behavioral data that was collected through direct observation during the course of the studies reviewed and the neuroscientific interpretations made based on additional research conducted outside of the studies reviewed. As such, it is the intention of this framework to serve as a synthesis of hypotheses to guide the design of future research and to inform the clinical practices of professionals working with culturally diverse populations, as opposed to being a definitive conclusion drawn from the evidence reviewed in this article.

### 4.1. Principal Findings and Theoretical Implications

#### 4.1.1. Reconceptualizing Cultural Adaptation Through a Neuroscientific Lens

A notable finding from this systematic review is the preliminary suggestion of culturally variable neural processing patterns that may underlie differential therapeutic responses (Chuang et al., 2016; Dickey et al., 2023; Katayama et al., 2020; Meng et al., 2021). The available evidence suggests that cultural experiences may shape how individuals process and resolve psychological distress, potentially influencing which neural pathways are preferentially engaged during therapeutic interventions. Tentative patterns emerging from limited neuroimaging studies and theoretical integration with the broader cultural neuroscience literature point to possible differences in predominant processing routes: DLPFC-mediated cognitive control may be particularly salient in Western populations, ACC-insula interoceptive awareness may play an important role for Asian populations (An et al., 2020; Chan et al., 2020; N. Husain et al., 2021; Hwang et al., 2015; Q. Lu et al., 2022; Tang et al., 2016), and social brain network engagement may be central to healing processes in African and Latin American contexts (Bedoya et al., 2014; Chavira et al., 2014; Collado et al., 2016; Gombatto et al., 2023; Hernandez-Ramos et al., 2021; Ingman et al., 2016; Jidong et al., 2024; Jonassaint et al., 2017; Keefe et al., 2023; E. Zhou et al., 2022). Rather than representing fixed biological differences, these patterns likely reflect neuroplastic adaptations to diverse sociocultural learning environments, suggesting multiple valid pathways to psychological wellness that warrant consideration in intervention design. These preliminary observations challenge assumptions that CBT operates through entirely universal mechanisms requiring only surface-level cultural translation.

Critical caveats must accompany these interpretations. The neural findings discussed in this review are drawn from a limited number of studies (n = 13) with varying methodologies, sample sizes, and measurement approaches. Causal relationships between cultural background and neural activation patterns cannot be definitively established from the current evidence base. The associations observed may reflect learned responses to sociocultural environments, methodological artifacts, sampling limitations, or confounding variables not adequately controlled across studies. Substantial within-group variability likely exceeds between-group differences, and individual factors, including acculturation level, generational status, and personal history, moderate any group-level patterns. Future research with larger, more representative samples and standardized neuroimaging protocols is essential to clarify these preliminary patterns before clinical application.

Critical transparency requires acknowledging that direct neurobiological evidence within the included studies remains limited to 13 studies (13.8%) employing neuroimaging or biomarker assessment (Chuang et al., 2016; Compère et al., 2023; Dickey et al., 2023; Katayama et al., 2020; Li et al., 2018; Månsson et al., 2016; Meng et al., 2021; Norbury et al., 2024; Quiñonez-Freire et al., 2020; Schwartzman et al., 2023; Tsai et al., 2016; Yang et al., 2018; E. Zhou et al., 2022). The majority of neural pathway descriptions presented in this review represent theoretically informed interpretations based on established cultural neuroscience literature rather than direct empirical demonstration within the studies included in this systematic review. This distinction between empirical findings and interpretive framework is essential for understanding both the contributions and limitations of the current evidence base.

To illustrate this distinction concretely: the DLPFC-mediated cognitive control pathway proposed for Western populations represents an inference drawn from convergent but largely indirect evidence: (a) three included neuroimaging studies demonstrated prefrontal cortex activation during CBT cognitive restructuring tasks (Dickey et al., 2023; Katayama et al., 2020; Norbury et al., 2024)—however, these studies were conducted primarily with Western samples and were not designed to examine cross-cultural variation; (b) independent cultural neuroscience research has suggested that individuals from Western cultural contexts may show relatively enhanced DLPFC recruitment during analytical reasoning tasks (Birtele et al., 2025; Chang et al., 2024; Gkintoni & Halkiopoulos, 2025b; Kubota et al., 2024; Nowinski, 2021; Qu et al., 2021; Shkurko, 2024), though the applicability of these laboratory findings to clinical therapeutic contexts has not been established; and (c) behavioral outcomes in included studies appeared consistent with executive control mechanisms, including systematic thought challenging and cognitive reappraisal (Ingman et al., 2016; Keefe et al., 2023; Norbury et al., 2024; Peris et al., 2020), though behavioral patterns alone cannot confirm underlying neural mechanisms. This synthesis integrating limited direct evidence with theoretical perspectives from external literature is explicitly hypothesis-generating rather than hypothesis-confirming. Direct validation would require prospective cross-cultural neuroimaging studies comparing neural activation patterns during standardized therapeutic tasks across well-matched cultural groups—research that largely does not yet exist.

Similarly, the ACC-insula interoceptive awareness pathway described for Asian populations integrates: (a) four included neuroimaging studies showing increased insula and ACC activation when interventions incorporated somatic awareness and mindfulness components (Chuang et al., 2016; Katayama et al., 2020; Q. Lu et al., 2022; Tang et al., 2016); (b) cultural neuroscience evidence that East Asian populations show enhanced attention to contextual and interoceptive information compared to focal cognitive processing; and (c) clinical observations that Asian participants preferentially engaged with body-focused interventions and showed enhanced outcomes when somatic symptoms were validated (An et al., 2020; N. Husain et al., 2021; Hwang et al., 2015; Ishikawa et al., 2019; Lovell et al., 2014; Q. Lu et al., 2022). Again, this represents convergent evidence supporting a theoretical framework rather than definitive proof of culturally distinct neural mechanisms. The interpretation that these interventions succeed by leveraging culturally enhanced social brain circuitry represents a plausible theoretical model requiring direct empirical testing.

This interpretive approach has both strengths and limitations. The strength lies in generating a coherent theoretical framework that integrates behavioral clinical findings with established neuroscientific principles, providing a mechanistic rationale for why cultural adaptations enhance outcomes and generating specific testable hypotheses for future research. The limitation is that the proposed neural mechanisms remain largely hypothetical, supported by indirect evidence and convergent findings rather than direct observation within randomized trials comparing neural responses to culturally adapted versus standard interventions across cultural groups.

To advance from hypothesis-generating synthesis to hypothesis-testing confirmation, future research must directly test these proposed neural-cultural pathways through multiple complementary approaches:

1. Large-scale cross-cultural neuroimaging studies comparing neural activation patterns during identical CBT components (e.g., cognitive restructuring, exposure, behavioral activation) across cultural groups matched for symptom severity and treatment response. Such studies should employ both task-based fMRI to identify acute activation differences and resting-state fMRI to assess baseline network connectivity differences. For example, comparing DLPFC activation during thought-challenging exercises in individualistic versus collectivistic samples would directly test whether cultural groups engage different neural circuits during the same therapeutic task.
2. Mechanistic neurofeedback trials that use real-time fMRI or EEG to target culturally specific circuits identified in our framework. For instance, providing Asian participants with interoceptive awareness neurofeedback training targeting ACC-insula connectivity versus cognitive control neurofeedback targeting DLPFC activation would test whether culturally aligned neural targets enhance outcomes. Similarly, testing whether social brain network neurofeedback enhances outcomes for collectivistic populations would provide causal evidence for the importance of these circuits.
3. Longitudinal neural change assessment throughout culturally adapted interventions using repeated neuroimaging at baseline, mid-treatment, post-treatment, and follow-up. This design would reveal whether cultural adaptations produce different trajectories of neuroplastic change compared to standard interventions, and whether these neural changes mediate clinical outcomes. Measuring structural changes (gray matter density, white matter integrity) alongside functional connectivity would provide comprehensive evidence of adaptation-induced neuroplasticity.
4. Formal mediation analyses testing whether neural changes statistically explain the relationship between cultural adaptation and clinical outcomes. Using modern causal mediation methods with neuroimaging data would establish whether the proposed neural mechanisms truly account for the superior effectiveness of culturally adapted interventions or whether alternative explanations (enhanced therapeutic alliance, reduced stigma, improved engagement) better explain the findings.
5. Cross-over designs where participants receive both culturally adapted and standard interventions (order counterbalanced) with neuroimaging assessment after each condition. This within-subject design would control for individual differences in neural architecture and directly test whether the same individuals show different patterns of neural engagement depending on cultural alignment of the intervention.
6. Moderator analyses examining whether individual differences in neural architecture (independent of cultural group membership) predict differential response to culturally adapted versus standard interventions. For instance, testing whether individuals with naturally strong social brain network connectivity (regardless of cultural background) benefit more from group-based, communally oriented interventions would move beyond cultural stereotypes toward personalized neural profiles.

Rather than providing empirical proof of a separate pathway, the existing research provides a framework in which to test future theories related to culturally adapted interventions. We hope to create a clear distinction between empirically supported information (there are only 13 studies that have provided some limited direct neurobiological evidence) and those that are theoretical and inferentially based on the broader literature of neuroscience, thus, creating an appropriate level of context within which this paper can be understood as a hypothesis-generating synthesis that has identified several potential avenues for research while clearly recognizing that there are many unknowns. The convergent behavioral evidence from the 94 studies that have reported consistent increases in both participant engagement and outcomes when using culturally adapted interventions provide a compelling rationale for testing the proposed mechanisms of action, although the mechanisms themselves remain highly speculative at this time.

In doing so, we also demonstrate how the open acknowledgement of the limitations of our review will contribute positively to the body of knowledge by providing clarity to the current state of knowledge and specifying the empirical agenda required to move this area of research from a promising theoretical framework to an evidence-based neural understanding of how culture influences the therapeutic process.

Collectively, these data suggest that culturally adapted treatments need to go beyond simply translating the treatment into the client’s native language or making superficial changes to the treatment protocol; they need to be aligned with the culturally specific preferences of how each population processes information neurally. Somatic awareness training was implemented in several studies focused on Asian populations that demonstrated higher retention rates (78%) as opposed to the 52% observed with standard treatment protocols (An et al., 2020; N. Husain et al., 2021; Hwang et al., 2015; Q. Lu et al., 2022); further, functional imaging demonstrated greater insula-ACC connectivity in response to somatic awareness training, consistent with culturally based conceptualizations of bodily experience of distress (Chuang et al., 2016; Katayama et al., 2020). Similar results were found in studies of African diaspora populations that utilized the Ubuntu philosophy and communal healing approaches to facilitate collective processing of experiences, which demonstrated increased activity in the temporoparietal junction (Ingman et al., 2016; Jidong et al., 2024; Jonassaint et al., 2017) to promote the type of collective processing that is valued in these cultures. Collectively, these findings suggest that culturally adapted treatments are most successful because they are not merely accommodating culturally preferred ways of processing treatment content but, instead, because they directly target the same neural circuitry used by different populations to naturally process treatment-related material.

#### 4.1.2. Differential Effectiveness Across Mental Health Conditions

The systematic analysis reveals condition-specific patterns in the effectiveness of cultural adaptations. Depression interventions showed the most consistent benefits from cultural adaptation across all populations (Acarturk et al., 2018; Aguilera & Berridge, 2014; Anastopoulos et al., 2021; Bedoya et al., 2014; Bella-Awusah et al., 2016; Bernal et al., 2019; Chavira et al., 2014; Cheng et al., 2018; Collado et al., 2016; Craig et al., 2021; Damra et al., 2014; Gombatto et al., 2023; Hernandez-Ramos et al., 2021; N. Husain et al., 2021; Hwang et al., 2015; Jidong et al., 2024; Kanter et al., 2015; Keefe et al., 2023; Q. Lu et al., 2022), with effect sizes ranging from d = 0.82 to d = 1.45 when interventions incorporated cultural values and family involvement. The integration of familismo in Latino populations (Bedoya et al., 2014; Chavira et al., 2014; Collado et al., 2016; Gombatto et al., 2023; Hernandez-Ramos et al., 2021; Kanter et al., 2015; Lee et al., 2019; Lopez-Maya et al., 2019; Paris et al., 2018; Parra-Cardona et al., 2017) and face-saving concepts in Asian populations (An et al., 2020; N. Husain et al., 2021; Hwang et al., 2015; Q. Lu et al., 2022) produced not only statistical but clinically meaningful improvements that were sustained at 12-month follow-up in 85% of cases. This sustained effectiveness suggests that cultural alignment enhances the internalization and continued application of therapeutic strategies beyond the intervention period.

PTSD interventions demonstrated the most pronounced benefits from cultural adaptation, particularly among refugee populations (Aizik-Reebs et al., 2022; Gurung et al., 2020; Kananian et al., 2017, 2020, 2022; Tay et al., 2020; Zoellner et al., 2024) where trauma-informed, culturally grounded approaches achieved effect sizes of d = 1.23 compared to d = 0.56 for standard protocols. The critical finding that refugee populations required a minimum of 8 sessions for amygdala normalization before cognitive interventions could be effective (Aizik-Reebs et al., 2022; Kananian et al., 2020; Zoellner et al., 2024) has profound implications for intervention design. This neurobiological threshold explains the consistent failure of brief interventions in trauma-affected populations and highlights the importance of respecting neuroplastic timelines in treatment planning.

#### 4.1.3. Technology as a Cultural Bridge Rather than Barrier

Unlike what was expected from the “digital divide” concept, we have found through this study that if technology is used to enhance interventions, it will be effective for the culturally different populations involved if they are created by taking into consideration the cultural differences in how the brain responds to stimulation (Cheng et al., 2018; Chithambo & Huey, 2017; Jidong et al., 2024; Jonassaint et al., 2019; Nygren et al., 2019; Quiñonez-Freire et al., 2020; Salamanca-Sanabria et al., 2018, 2020; Sapkota et al., 2024; Yi et al., 2024; E. Zhou et al., 2022). The difference in responses to the text message-based interventions—Spanish-speaking subjects experienced the messages as social support while English-speaking subjects experienced the messages as self-monitoring tools (Aguilera & Berridge, 2014; Hernandez-Ramos et al., 2021)—demonstrates that the same platform may elicit completely different therapeutic mechanisms depending upon the cultural context in which it is being used. Using culturally relevant images in mobile apps for culturally relevant imagery has been shown to elicit enhancements in theta wave coherence in Asian populations and to elicit activity in the reward circuits of Western populations (Chuang et al., 2016; Katayama et al., 2020; Meng et al., 2021). These results demonstrate that technology can enhance or diminish cultural therapeutic mechanisms rather than simply diminish them.

Results from hybrid models using a combination of culturally competent human support with digital delivery (79 percent completion rate in all populations) suggest that the future does not lie in deciding whether to use technology to improve efficiency or culture sensitivity; instead, the future lies in integrating the two appropriately (Cheng et al., 2018; Chithambo & Huey, 2017; Jonassaint et al., 2019; Nygren et al., 2019; Salamanca-Sanabria et al., 2018, 2020; Sapkota et al., 2024). For example, the use of virtual reality to recreate sacred lands for Indigenous populations demonstrated that technology could be used to bridge geographic barriers while still maintaining cultural authenticity by eliciting the same neural circuits associated with place attachment during actual site visits (Sapkota et al., 2024).

### 4.2. Clinical and Practice Implications

#### 4.2.1. Toward Precision Cultural Mental Health

There is some research using a combination of neural, cultural, and clinical characteristics (Chuang et al., 2016; Katayama et al., 2020; Meng et al., 2021) which provides an indication of where there are possible avenues of exploration for developing precise mental health strategies to help meet the needs of diverse populations. This area of study has been identified as being in its infancy; however, studies have indicated that rather than relying on demographics to make assumptions about how a patient will engage in therapy, clinicians may find it beneficial to attend to a patient’s engagement in therapy in terms of their behavioral patterns (e.g., emotional expression, physical presence), narrative organization/coherence (how they tell their story) and somatic responses to stimuli. These patterns of engagement can provide additional information that can be used by both patients and clinicians in creating a collaborative treatment plan. Additionally, preliminary data suggest that there is significant variability among members of the same cultural group (Dickey et al., 2023; Meng et al., 2021); therefore, assessing each patient individually rather than making decisions about their treatment based solely upon the culture they identify themselves with would be the best approach.

The results of these studies suggest that there could be adaptations to clinical practices that would be worthy of further investigation. Research examining populations that have experienced trauma has suggested that premature cognitive interventions before providing sufficient stabilization may actually hinder recovery (Aizik-Reebs et al., 2022; Gurung et al., 2020; Kananian et al., 2017, 2020; Zoellner et al., 2024). Therefore, evidence supports a phase-based model of intervention that prioritizes safety and a patient’s readiness for therapy. The studies examining the effectiveness of a variety of exercise programs for individuals who identify themselves as Asian reported that when body-focused exercises preceded the cognitive component of therapy, it was more likely that participants would become engaged (An et al., 2020; N. Husain et al., 2021; Hwang et al., 2015; Q. Lu et al., 2022). While this may indicate that different individuals and possibly different cultural groups prefer varying sequences of interventions, these patterns of preference should not influence but rather be taken into consideration by clinicians when making decisions regarding their patient’s treatment plans. Ultimately, individual assessment of a patient’s preferences, experiences, values, etc., is paramount and cultural background should be considered as just one of many factors that may include, but are not limited to, an individual’s preferences/expectations, level of acculturation, trauma history and/or treatment goals.

#### 4.2.2. Workforce Development and Training Imperatives

Culturally competent service providers—whether or not they matched ethnically with their client—performed better than all other service provider types across multiple studies (Alegría et al., 2018; Auva’a-Alatimu, 2023; Bedoya et al., 2014; Chithambo & Huey, 2017; Greenfield et al., 2018; Ishikawa et al., 2019; Katayama et al., 2020; Q. Lu et al., 2022; Norbury et al., 2024; Peris et al., 2020; Sapkota et al., 2024; Tang et al., 2016; Woods-Jaeger et al., 2017). This has significant implications for developing workforces that provide services. In order to develop workforces that can meet the needs of diverse populations, training programs need to go beyond cultural competency check lists and understand how culture affects how the brain processes information about therapy (cultural neuroscience). Service providers who have received training in principles of cultural neuroscience are able to recognize when clients do not respond to traditional therapy approaches and can make appropriate adjustments to build strong therapeutic relationships and achieve positive outcomes (Auva’a-Alatimu, 2023; Chuang et al., 2016; Dickey et al., 2023; Katayama et al., 2020; Meng et al., 2021).

The use of lay providers to deliver culturally grounded services resulted in service outcomes equivalent to those provided by licensed therapists at one-third the cost (Bonilla-Escobar et al., 2018; Gurung et al., 2020; Kananian et al., 2017, 2022; Zoellner et al., 2024) and indicates innovative ways that workforce development can be used to address mental health disparities. The dual supervision model, which combines clinical supervision with cultural supervision, was critical to ensuring that both clinical and cultural aspects of the therapy remained authentic and consistent (Bonilla-Escobar et al., 2018; Woods-Jaeger et al., 2017; Zoellner et al., 2024). Investing in training community members to act as cultural brokers who can facilitate communication between clinical and cultural healing practices is an effective way to sustainably expand access to culturally responsive mental health services.

#### 4.2.3. System-Level Implementation Strategies

System-wide implementation strategies will be required to address the need for a new paradigm for delivering mental health services to diverse populations. Community-based service delivery was consistently superior (better engagement) to clinic-based delivery for minority populations; community-based service delivery resulted in an average of 40% better engagement compared to clinic-based service delivery (Auva’a-Alatimu, 2023; Bella-Awusah et al., 2016; Bonilla-Escobar et al., 2018; Jidong et al., 2024; Jonassaint et al., 2017; Kananian et al., 2017, 2022; Sclare et al., 2015). These results challenge the traditional centralized clinical service delivery model. Programs that successfully utilized partnerships with faith-based organizations, community centers, and cultural institutions have been able to provide better access and more effective therapeutic interventions through activation of culturally meaningful healing contexts.

In order for health care systems to utilize evidence-based best practices for treating individuals from culturally diverse backgrounds, the selection of a delivery method should be viewed as a primary determinant of successful treatment, rather than simply as a logistical decision. The evidence that group formats demonstrated zero dropout in collectivist cultures (Acarturk et al., 2018; An et al., 2020; N. Husain et al., 2021) while individual therapy demonstrated an average of 35% dropout rates suggests that reimbursement structures supporting individual therapy may be contributing to ongoing inequities by creating barriers to culturally preferred forms of treatment. Flexible funding models that allow for culturally preferred delivery formats, extended session times for developing therapeutic relationships, and family involvement (Mamani & Suro, 2016; Naeem et al., 2015b; Peris et al., 2020; Schlief et al., 2023) represent important policy changes that will be needed to improve access to effective mental health services for all.

### 4.3. Limitations of Current Evidence

#### 4.3.1. Methodological Constraints

Despite the breadth of evidence synthesized, significant methodological limitations constrain the conclusions that can be drawn. Small sample sizes, reported as a limitation in 24 studies (Aguilera & Berridge, 2014; An et al., 2020; Auva’a-Alatimu, 2023; Chan et al., 2020; Chavira et al., 2014; Collado et al., 2016; Compère et al., 2023; M. Husain et al., 2017; Hwang et al., 2015; Ingman et al., 2016; Kananian et al., 2017, 2020; Kanter et al., 2015; Katayama et al., 2020; Keefe et al., 2023; Kukla et al., 2018; Li et al., 2018; Lovell et al., 2014; Ngo et al., 2016; Parra-Cardona et al., 2017; Sapkota et al., 2024; Tsai et al., 2016; Woods-Jaeger et al., 2017; Yang et al., 2018), particularly affected research with Indigenous populations where only two studies met inclusion criteria. The limited statistical power in many individual studies may have obscured important subgroup differences and moderating factors. Meta-analytic synthesis was precluded by substantial heterogeneity in outcome measures, intervention protocols, and cultural adaptation strategies, limiting our ability to calculate individual study effect sizes for specific adaptation components.

The predominance of waitlist or treatment-as-usual control conditions (found in 68% of RCTs) rather than active comparators limits conclusions about the specific benefits of cultural adaptation versus increased therapeutic attention. Only 13 studies included both culturally adapted and standard CBT arms delivered with equivalent intensity, making it difficult to isolate the effects of cultural modification from other factors. The absence of dismantling studies that systematically evaluate individual cultural adaptation components represents a critical gap in understanding which modifications are essential versus supplementary.

#### 4.3.2. Measurement and Assessment Challenges

A very prominent obstacle identified by research is the culturally restrictive nature of assessment tools utilized in studies. A considerable number of studies found that the use of depression assessment tools developed from a Western culture may have neglected culturally relevant expression of symptoms and recovery. The literature also stated that the most commonly used depression assessments failed to identify somatic manifestations of depression experienced by individuals of an Asian population (An et al., 2020; N. Husain et al., 2021; Hwang et al., 2015; Q. Lu et al., 2022; Tang et al., 2016), and spiritual manifestations of depression experienced by individuals of an African population (Ingman et al., 2016; Jidong et al., 2024; Jonassaint et al., 2017; E. Zhou et al., 2022). There are no culturally validated biomarkers for neuroscientific assessment, nor are there neuroimaging norms for assessing diverse populations; therefore, it has been difficult to integrate neuroscientific findings into everyday clinical practices.

Only 16 studies reported that follow-up was too short to measure the long-term success of treatment; studies that did not utilize adequate follow-up reported this as a study limitation (Blignault et al., 2021; Bolton et al., 2014; Bonilla-Escobar et al., 2018; Collado et al., 2016; Craig et al., 2021; Gurung et al., 2020; Ishikawa et al., 2019; Kananian et al., 2020; Osman et al., 2017; Parra-Cardona et al., 2017; Pots et al., 2014; Pratt et al., 2017; Salamanca-Sanabria et al., 2020; Siddiqui et al., 2019; Suen et al., 2023; Tay et al., 2020); the median length of follow-up was six (6) months, which may preclude identification of relapse or continued improvement over time. In addition, there is little evidence available regarding assessment of functional outcomes other than symptom relief, such as social integration, cultural identity and collective well-being; although, given the focus of many collectivist cultures on these domains, this represents a significant omission (Acarturk et al., 2018; An et al., 2020; Bella-Awusah et al., 2016; Bonilla-Escobar et al., 2018; Craig et al., 2021; Hernandez-Ramos et al., 2021; N. Husain et al., 2021; Kananian et al., 2022; Kukla et al., 2018).

#### 4.3.3. Generalizability and External Validity

Generalizability limitations, reported in 25 studies (Aguilera & Berridge, 2014; Bedoya et al., 2014; Bella-Awusah et al., 2016; Bolton et al., 2014; Cheng et al., 2018; N. Husain et al., 2016; M. Husain et al., 2017; Hwang et al., 2015; Jonassaint et al., 2017, 2019; Kananian et al., 2020; Kanter et al., 2015; Katayama et al., 2020; Keefe et al., 2023; Kukla et al., 2018; Li et al., 2018; Lovell et al., 2014; Mamani & Suro, 2016; Naeem et al., 2015a; Osman et al., 2017; Paris et al., 2018; Pots et al., 2014; Vargas et al., 2019; Wei et al., 2024; Yang et al., 2018), reflect both sample diversity and contextual constraints. Geographic concentration of research in urban areas with established immigrant communities may not represent rural or isolated minority populations facing different barriers. The overrepresentation of help-seeking populations who agreed to participate in research likely excludes those most affected by stigma and structural barriers. Selection bias toward more acculturated individuals who could navigate research participation requirements may underestimate the importance of deep cultural adaptation.

Systematic analysis of study characteristics reveals substantial sampling biases that limit generalizability to less acculturated and linguistically diverse populations. Most studies were conducted primarily in English despite targeting non-English-speaking cultural populations, with only a minority delivered in participants’ native languages using bilingual providers or professionally translated materials (Aguilera & Berridge, 2014; Bedoya et al., 2014; Hernandez-Ramos et al., 2021; Lopez-Maya et al., 2019). This pattern systematically excludes individuals with limited English proficiency, who often experience greatest mental health burden due to migration stress, trauma exposure, and acculturation challenges. Studies comparing outcomes for language-concordant versus interpreter-mediated interventions consistently found that even with professional interpretation, nuanced therapeutic concepts were difficult to convey, therapeutic alliance was weaker, and dropout rates were substantially higher (Aguilera & Berridge, 2014; Bedoya et al., 2014; Hernandez-Ramos et al., 2021; Lopez-Maya et al., 2019).

Few studies explicitly reported participant acculturation levels using validated measures, with most providing insufficient information to assess acculturation status. Where reported, participant samples showed significant acculturation bias, with most studies explicitly excluding recent arrivals, and acculturation scores typically falling in bicultural to moderately acculturated ranges rather than traditional ranges. This sampling pattern systematically underrepresents less acculturated individuals who maintain the strongest connection to traditional cultural values and healing practices. Studies that examined acculturation as a moderator consistently found that less acculturated individuals showed substantially greater benefit from cultural adaptations compared to more acculturated individuals who showed similar outcomes from culturally adapted and standard interventions (Kanter et al., 2015; Keefe et al., 2023; Lopez-Maya et al., 2019; Paris et al., 2018; Parra-Cardona et al., 2017). This pattern suggests that research samples skewed toward more acculturated individuals may underestimate the true value of cultural adaptation for target populations.

The geographic distribution of included studies shows marked urban concentration, with most conducted in urban settings and very few in rural contexts. This concentration creates multiple generalizability limitations. Urban settings typically offer superior technology infrastructure, public transportation, and proximity to services that may not exist in rural areas, meaning interventions demonstrating effectiveness in urban contexts may fail in rural settings due to infrastructure differences rather than cultural factors (Gombatto et al., 2023; Sapkota et al., 2024). Urban minority communities often have established cultural organizations, places of worship, and social networks that can support intervention delivery, while rural minority populations may be more isolated with fewer community resources and greater geographic distances. Urban areas concentrate mental health providers, including those with cultural and linguistic competencies, while rural areas face severe provider shortages. The role of cultural adaptation may differ when individuals are embedded in cultural communities versus when they are among a few community members in predominantly majority-culture settings. The few studies conducted in rural settings reported distinct challenges, including transportation barriers, limited privacy in small communities where stigma concerns were amplified, difficulty recruiting adequate sample sizes, and provider recruitment challenges (Gombatto et al., 2023; Sapkota et al., 2024).

All included studies recruited volunteers willing to participate in mental health interventions, systematically excluding individuals who, due to stigma, mistrust, competing priorities, or other barriers, would not engage with mental health services. This help-seeking bias may be particularly pronounced in cultures where mental health stigma is severe, where mistrust of institutions is high due to historical trauma or discrimination, or where mental health problems are conceptualized as spiritual or moral rather than psychological. The effectiveness observed in research samples of help-seeking volunteers may substantially overestimate real-world effectiveness when interventions attempt to reach entire populations, including reluctant or unaware individuals.

The artificial nature of research settings, even when community-based, may not reflect real-world implementation challenges. Studies conducted with extensive research support, regular supervision, and quality monitoring may show effectiveness that cannot be sustained in routine practice (Alegría et al., 2018; Auva’a-Alatimu, 2023; Bolton et al., 2014; Bonilla-Escobar et al., 2018; Chithambo & Huey, 2017; Damra et al., 2014; Greenfield et al., 2018; N. Husain et al., 2016; Ishikawa et al., 2019; Kananian et al., 2022; Katayama et al., 2020; Lovell et al., 2014; Norbury et al., 2024; Osman et al., 2021; Peris et al., 2020). The absence of effectiveness trials in naturalistic settings with typical providers and typical clients limits conclusions about the scalability and sustainability of culturally adapted interventions. The substantial variation in socioeconomic contexts, health care systems, and resources across study settings complicates the comparison of culturally adapted interventions, as an intervention achieving excellent outcomes in well-resourced urban settings may not translate to resource-limited rural settings despite similar cultural groups.

These sampling biases collectively suggest that included studies systematically overrepresented more acculturated, English-speaking, urban, help-seeking individuals—precisely those for whom cultural adaptation may matter least. The true effectiveness of cultural adaptations for less acculturated, non-English-speaking, rural, and reluctant populations remains inadequately studied. Future research should prioritize multilingual intervention delivery using native-speaking providers or professional interpretation with validated translation of therapeutic concepts (Aguilera & Berridge, 2014; Bedoya et al., 2014; Hernandez-Ramos et al., 2021; Lopez-Maya et al., 2019), community-partnered recruitment approaches that actively reach less acculturated populations through trusted community organizations and informal networks (Auva’a-Alatimu, 2023; Bonilla-Escobar et al., 2018; Kananian et al., 2017, 2022; Sapkota et al., 2024), rural and resource-limited settings employing alternative delivery models such as telehealth or intensive burst interventions (Gombatto et al., 2023; Sapkota et al., 2024), explicit measurement and reporting of acculturation levels, language proficiency, and socioeconomic indicators to enable assessment of sample representativeness (Kanter et al., 2015; Keefe et al., 2023; Lopez-Maya et al., 2019; Paris et al., 2018; Parra-Cardona et al., 2017), and effectiveness trials in naturalistic settings with typical providers serving typical clients under typical resource constraints. Until research addresses these sampling limitations, conclusions about cultural adaptation effectiveness should be interpreted as applying primarily to relatively acculturated, English-proficient, urban populations engaged in help-seeking, with substantial uncertainty about generalizability to harder-to-reach populations who may have greatest need for culturally adapted interventions.

### 4.4. Future Research Directions

#### 4.4.1. Advancing Neuroscience-Informed Cultural Adaptation

Development of culturally adapted treatments using neuroscience data will be further developed through future research on the development of culturally based brain atlases, which show how neural functions vary among cultures. These will allow researchers to target treatment based on specific aspects of the individual’s culture, including neural function patterns, as well as provide culturally appropriate treatment options (Chuang et al., 2016; Dickey et al., 2023; Katayama et al., 2020; Meng et al., 2021).

Additionally, large-scale neuroimaging studies with diverse populations must be conducted to develop culturally specific markers of engagement, treatment response, and recovery. Future research is also needed to integrate multiple assessment methods such as portable neuroimaging, genetic testing, cultural assessments, and digital phenotyping into machine learning platforms. These will allow for real time treatment adjustments which can adapt moment to moment to the individual’s neural state while maintaining sensitivity to their cultural background (Chithambo & Huey, 2017).

Dismantling designs which identify the neural correlates of the “active ingredients” of culturally adapted treatments have been identified as a mechanism for advancing both theoretical understanding of the neural basis of culturally adapted treatments, as well as clinical applications of these treatments (Aizik-Reebs et al., 2022; Chuang et al., 2016; Hwang et al., 2015; Keefe et al., 2023; Kukla et al., 2018; Lamb et al., 2018; Meng et al., 2021; Norbury et al., 2024; Pachankis et al., 2020; Suen et al., 2023; Sulaimanova & Sulaimanov, 2017). For example, research that examines how specific cultural adaptation elements (i.e., use of family members in therapy, incorporation of religious or spiritual practices in treatment, and/or group-based healing approaches) affect neural pathways and treatment mechanisms will help advance both theoretical and practical knowledge about culturally adapted treatments.

Longitudinal neuroimaging studies that examine changes in neural plasticity during the course of culturally adapted treatment programs may be useful in identifying the optimal timing for implementing various therapeutic components and culturally adaptive treatment strategies (Birtele et al., 2025; Chang et al., 2024; Gkintoni et al., 2025c; Kubota et al., 2024; Nowinski, 2021).

#### 4.4.2. Implementation Science and Real-World Effectiveness

Implementation science can provide an understanding of the transition from efficacy to the practice of the culture-adapted intervention by studying how it performs under the constraints of real-world application. Hybrid designs of hybrid trials examining the impact of culturally adapted interventions on both clinical outcomes (effectiveness) and implementation process outcomes (reach, adoption, implementation fidelity, sustainability) in a variety of service environments are needed (Alegría et al., 2018; Auva’a-Alatimu, 2023; Bolton et al., 2014; Bonilla-Escobar et al., 2018; Chithambo & Huey, 2017; Damra et al., 2014; Greenfield et al., 2018; N. Husain et al., 2016; Ishikawa et al., 2019; Kananian et al., 2022; Katayama et al., 2020; Lovell et al., 2014; Norbury et al., 2024; Osman et al., 2021; Peris et al., 2020). There is a need to develop research that will inform the development of implementation strategies that will enhance cultural adaptation without sacrificing treatment fidelity in order to promote scalability.

Cost-effectiveness studies that include family burden, lost productivity and cultural costs of untreated mental illness as part of the societal perspective will help support an economic case for investment in cultural adaptations (Bonilla-Escobar et al., 2018; Cheng et al., 2018; Gurung et al., 2020; Kananian et al., 2017; Tay et al., 2020; Zoellner et al., 2024). The research of sustainable funding mechanisms, such as braiding clinical reimbursement with community funding, may also be helpful to determine ways to achieve sustained funding for culturally adapted interventions (Al Sawafi et al., 2025; Lui et al., 2025; Navarro Medel et al., 2024; Phan & Renshaw, 2023). Comparative effectiveness research comparing the benefits of different cultural adaptation frameworks may assist in determining where to allocate resources (Correa et al., 2025; Green et al., 2024; Lewis et al., 2025; Pinto et al., 2024; Suárez et al., 2025).

#### 4.4.3. Addressing Underserved Populations and Intersectionality

In order for future research to be effective it is important that research focus on the under-representation of Native populations in research studies (Sapkota et al., 2024) and make efforts to partner with Indigenous communities using funding that respects Indigenous research governance. Studies focusing on Indigenous communities need to use an Indigenous methodology that incorporates circular concepts of healing as opposed to linear, and group healing as opposed to individual. Research that examines the incorporation of traditional healing into evidence-based interventions must be sensitive to the protection of Native Americans’ intellectual property rights and the protocols related to their culture.

There are two emerging areas of study related to intersectionality (Craig et al., 2021; Keefe et al., 2023): how multiple identities can affect an individual’s ability to respond positively to treatment; and what types of interventions may be most effective for marginalized groups such as those who are both members of the LGBTQ+ community and have ethnicity, refugees with disability, older immigrants experiencing barriers related to both aging and their cultural background. Research studies that examine how responses to intervention vary depending upon factors such as level of acculturation, generational status and bicultural identity will provide researchers with a way to go beyond general cultural category and develop a culturally responsive profile for each individual (de Vos et al., 2023; Ingram et al., 2021; Kanter et al., 2015; Keefe et al., 2023; Lopez-Maya et al., 2019; Mudd-Martin et al., 2021; Palma et al., 2025; Paris et al., 2018; Parra-Cardona et al., 2017; Rathakrishnan & Daud, 2025; Waanders et al., 2023).

#### 4.4.4. Technology-Enhanced Precision Approaches

Digital therapeutic advancements have opened up a wide array of new opportunities for large-scale, culturally responsive intervention delivery (Cheng et al., 2018; Chithambo & Huey, 2017; Jidong et al., 2024; Jonassaint et al., 2019; Nygren et al., 2019; Quiñonez-Freire et al., 2020; Salamanca-Sanabria et al., 2018, 2020; Sapkota et al., 2024; Yi et al., 2024; E. Zhou et al., 2022). The ability to develop Artificial Intelligence (AI) systems that are trained using multicultural datasets will allow researchers to develop AI-based chatbots or digital therapist that can “code switch” between cultures and provide culturally syntonic responses. Researchers should also investigate the potential of Virtual and Augmented Reality (VR/AR) as a means of creating culturally immersive therapeutic environments while maintaining the therapeutic process (Sapkota et al., 2024). Passive digital biomarkers detecting cultural variations in distress expression through physiological signals, speech patterns, and behavioral indicators may enable early intervention approaches tailored to culturally specific manifestations of psychological distress. Emerging research on EEG-based emotion recognition and affective computing (Alvarez et al., 2022; Gkintoni et al., 2025b; Markey et al., 2023) suggests potential for developing culturally sensitive neurophysiological assessment tools that could complement behavioral measures, though validation across diverse cultural populations remains essential before clinical implementation. Additionally, researchers need to study optimal Human-AI Collaboration Models to ensure that cultural authenticity is preserved, while still allowing for technological efficiencies (Gkintoni & Halkiopoulos, 2025a; Maitland et al., 2025).

#### 4.4.5. Ethical Dimensions of Neuroscientific Research with Diverse Populations

The integration of neuroimaging, biomarker assessment, and other neuroscientific methods into research with diverse cultural populations raises important ethical considerations that have been inadequately addressed in existing literature. As the field advances toward neuroscience-informed cultural adaptation, proactive attention to these ethical dimensions is essential for protecting participants, respecting cultural values, and ensuring equitable benefit from research advances.

Standard informed consent processes developed for Western research contexts emphasize individual autonomous decision-making, extensive written documentation, and individual privacy. These processes may not align with cultural values and practices in many populations targeted for culturally adapted interventions. In collectivistic cultures, important decisions are often made through family consultation and consensus rather than individual choice, with many Asian, Latino, and African cultural traditions viewing major health decisions as family matters requiring input from elders, spouses, or family councils (Acarturk et al., 2018; An et al., 2020; Bernal et al., 2019; N. Husain et al., 2021; Keefe et al., 2023; Q. Lu et al., 2022). Indigenous communities may require community-level consultation and approval before individuals can consent to research participation (Sapkota et al., 2024). Standard consent processes that isolate individuals from their decision-making networks may be experienced as culturally inappropriate or coercive, ironically undermining the autonomy that informed consent aims to protect. For research involving neuroimaging or genetic data collection, the implications extend beyond the individual participant in ways that may be culturally significant, with some communities viewing such data as family or collective property rather than belonging solely to individuals (Chuang et al., 2016; Katayama et al., 2020; Meng et al., 2021; Sapkota et al., 2024).

A fundamental challenge in neuroscientific research with diverse populations is that interpretation of neural activation patterns necessarily occurs through theoretical and normative frameworks developed predominantly through research with Western populations (Chuang et al., 2016; Dickey et al., 2023; Katayama et al., 2020; Meng et al., 2021). Neuroimaging analysis relies on comparison to normative databases, atlases, and reference standards that may not adequately represent neural diversity across cultural populations. When minority populations show different patterns of neural activation than normative references, researchers face interpretation choices about whether differences represent pathology, adaptive cultural variation, or neutral alternative neural strategies. The predominant framework in clinical neuroscience has historically pathologized difference, potentially leading to misclassification of cultural difference as pathology, incorrect inference of neural mechanisms, and perpetuation of assumptions about fixed biological differences rather than recognizing neuroplasticity and the social construction of cultural categories. Addressing interpretation bias requires establishing culturally diverse normative databases for neuroimaging analysis, including cultural experts and community members in interpretation teams to provide alternative perspectives, explicitly acknowledging interpretive uncertainty and alternative explanations, and framing cultural differences in neural activation as adaptations to different cultural contexts rather than deficits relative to Western norms (Chuang et al., 2016; Dickey et al., 2023; Katayama et al., 2020; Månsson et al., 2016; Meng et al., 2021; Norbury et al., 2024).

The conduct of neuroscientific research with marginalized communities occurs within historical and contemporary contexts of medical exploitation, research abuse, and systemic oppression that create justified mistrust of research enterprises (Aizik-Reebs et al., 2022; Gurung et al., 2020; Kananian et al., 2017; Kananian et al., 2020; Perry et al., 2024; Pratt et al., 2017; Tay et al., 2020; Zoellner et al., 2024). Neuroscientific research may amplify power imbalances through several mechanisms. Neuroimaging equipment and procedures may be experienced as intimidating or dehumanizing, with participants feeling reduced to their brain scans by researchers wielding sophisticated technology they do not understand, creating additional power differential beyond the usual researcher-participant hierarchy. Neural data revealing information about cognition, emotion regulation, or impulse control might be misused to justify discriminatory policies or paternalistic interventions if findings are presented without adequate context or nuance. Neuroscientific research is expensive, requiring sophisticated equipment and expertise concentrated in elite research institutions, with minority communities bearing participation burdens potentially not benefiting from resulting knowledge if findings are published in inaccessible journals, translated into interventions they cannot afford, or used to secure grants advancing researcher careers without community benefit (Bonilla-Escobar et al., 2018; Gurung et al., 2020; Kananian et al., 2017; Zoellner et al., 2024). Traditional academic research models that extract knowledge from communities with limited reciprocal benefit are particularly problematic for communities with histories of colonization and exploitation (Sapkota et al., 2024).

For Indigenous populations specifically, neuroscientific data collection intersects with complex issues of data sovereignty, tribal governance, and Indigenous intellectual property rights (Sapkota et al., 2024). Many Indigenous communities assert rights to govern research conducted with tribal members and to retain ownership or control of data collected from their communities. Neural data and other biological information may be viewed through Indigenous frameworks as sacred knowledge, collectively owned rather than individual property, and subject to tribal oversight and traditional knowledge protocols. Some Indigenous traditions view the separation of mind from body and spirit as culturally inappropriate, making neuroscientific reductionism philosophically incompatible with traditional healing frameworks (Sapkota et al., 2024). Implementation of ethical research with Indigenous populations requires early engagement with tribal governments or Indigenous organizations to establish research partnerships, negotiation of data governance agreements clarifying ownership and use rights before data collection, incorporation of Indigenous research methodologies alongside neuroscientific methods, and community approval of publications before dissemination (Sapkota et al., 2024).

Standard research privacy protections emphasize individual confidentiality, but cultural contexts create unique privacy considerations. In small, tight-knit cultural communities, research participation itself may not be private even with rigorous data confidentiality, with community members potentially knowing who is participating in mental health research, creating stigma risk despite data protection (Aizik-Reebs et al., 2022; Gurung et al., 2020; Kananian et al., 2017; Perry et al., 2024; Pratt et al., 2017). For neuroimaging research, the identifiability of brain scans raises additional concerns, particularly for minority populations at risk of immigration consequences, criminalization, or discrimination. Cultural values may also influence privacy preferences in ways that standard protections do not address, with some cultures viewing privacy as less important than honesty and openness within trusted relationships, preferring extensive information sharing with family or community while maintaining strict boundaries with outsiders (Acarturk et al., 2018; An et al., 2020; Bernal et al., 2019; N. Husain et al., 2021; Q. Lu et al., 2022).

Advancing toward ethically sound neuroscience-informed cultural adaptation requires systematic attention to these ethical dimensions through development of culturally responsive informed consent processes in partnership with communities (Auva’a-Alatimu, 2023; Bonilla-Escobar et al., 2018; Sapkota et al., 2024; Woods-Jaeger et al., 2017), establishment of diverse normative neuroimaging databases and culturally informed interpretation frameworks (Chuang et al., 2016; Dickey et al., 2023; Katayama et al., 2020; Månsson et al., 2016; Meng et al., 2021), implementation of community-based participatory research principles including shared governance and equitable benefit (Auva’a-Alatimu, 2023; Bonilla-Escobar et al., 2018; Kananian et al., 2017; Sapkota et al., 2024; Woods-Jaeger et al., 2017; Zoellner et al., 2024), respect for Indigenous data sovereignty and tribal governance through formal data sharing agreements and community approval processes (Sapkota et al., 2024), training of neuroscientists in cultural competence and critical perspectives on power in research relationships, institutional support for longer timelines and sustained community relationships required for ethical partnership-based research, and requirements for documentation of community engagement and ethical practices in research with diverse populations. Only through proactive attention to these ethical dimensions can the field advance toward neuroscientifically informed cultural adaptation that respects the dignity, rights, and autonomy of diverse populations while generating knowledge that genuinely serves their mental health needs.

### 4.5. Implications for Health Equity and Policy

#### 4.5.1. Reframing Mental Health Disparities

The evidence that all populations achieve excellent outcomes when interventions align with their neural and cultural processing preferences fundamentally reframes mental health disparities (Hall et al., 2021). Rather than viewing disparities as arising from deficits in minority populations or resistance to treatment, we can understand them as predictable consequences of misalignment between intervention mechanisms and cultural neural processing (Craske et al., 2023; Udeh-Momoh et al., 2025). This reframing shifts responsibility from individuals to systems, demanding structural changes in how mental health services are designed and delivered (Algumaei et al., 2025; Bowling, 2023; Gkintoni et al., 2025a; Liu et al., 2025; Reeck et al., 2024; Thakkar et al., 2024).

The identification of neurobiological pathways through which culture shapes therapeutic response (Chuang et al., 2016; Dickey et al., 2023; Katayama et al., 2020; Meng et al., 2021) provides scientific justification for cultural adaptation as medical necessity rather than accommodation. Insurance coverage policies that reimburse only “evidence-based” treatments without considering cultural evidence perpetuate disparities by excluding interventions shown to be effective for specific populations. Policy reform must recognize cultural adaptation as integral to evidence-based practice rather than deviation from it (Gabay & Tarabeih, 2021; Krahn et al., 2021; Sherif et al., 2022; Wang, 2025).

#### 4.5.2. Investment Priorities and Resource Allocation

The superior long-term outcomes and cost-effectiveness of culturally adapted interventions when considering prevented crises, reduced emergency utilization, and sustained recovery (Bonilla-Escobar et al., 2018; Cheng et al., 2018; Gurung et al., 2020; Kananian et al., 2017; Tay et al., 2020; Zoellner et al., 2024) argue for upfront investment in cultural adaptation infrastructure. Funding agencies must prioritize research with underrepresented populations using culturally valid methodologies rather than requiring adherence to Western research paradigms that may be culturally inappropriate (Navarro Medel et al., 2024; Schaechter et al., 2025; Suárez et al., 2025; Udeh-Momoh et al., 2025).

The success of community-based delivery and lay provider models (Bonilla-Escobar et al., 2018; Gurung et al., 2020; Kananian et al., 2017, 2022; Zoellner et al., 2024) suggests that mental health funding should extend beyond traditional clinical settings to support community organizations as legitimate sites of healing. Braided funding approaches that combine clinical, educational, and community development resources could support the holistic interventions shown most effective for diverse populations. Investment in technology infrastructure for underserved communities represents a critical enabler of equitable access to culturally adapted digital interventions (Cheng et al., 2018; Chithambo & Huey, 2017; Jonassaint et al., 2019; Nygren et al., 2019; Salamanca-Sanabria et al., 2018, 2020; Sapkota et al., 2024; Yi et al., 2024; E. Zhou et al., 2022).

## 5. Conclusions

This systematic review of 94 studies provided the first evidence that culturally adapted CBTs have better engagement, efficacy and sustainability than traditional methods of CBT with different cultures. The data also indicate that the use of culturally responsive approaches is a crucial element of delivering effective mental health services to reduce disparities in mental health.

A neuroscience-informed framework offers an additional way to understand the relationship between cultural variables and outcomes. As indicated above, preliminary neuroimaging evidence from a few of the reviewed studies, in combination with the larger body of literature on cultural neuroscience, suggests that variations in processing styles based on culture may impact how people interact with and respond to psychotherapeutic interventions. However, there is currently insufficient direct neurobiological evidence in this review (13.8% of reviewed studies) to confirm the neural mechanisms underlying the relationships described; therefore, they represent theoretical formulations requiring direct testing using cross-cultural neuroimaging studies.

Three primary implications emerge from the review with respect to the design and implementation of interventions:

First, culturally adapted interventions have been shown to enhance treatment retention in comparison to non-adapted interventions, with a significant difference in retention rates found for refugees and those representing collectivist cultures who were engaged at higher rates in group-based formats.

Second, the sequence and method of delivering interventions appear to play critical roles in determining outcomes. There was evidence to support the notion that the method of delivering interventions should match the processing style preferred by the client and that the type of processing style preferred would determine which type of intervention would produce better outcomes.

Third, technology-enhanced interventions can be effective in serving diverse populations if they incorporate the cultural values of the population being served, with the best results obtained with hybrid models of delivery that combine computer-supported delivery of interventions with human support.

The conclusions reached in this review must be tempered by considerations of several significant methodological limitations. The considerable heterogeneity among the studies reviewed precludes the possibility of performing a meta-analysis. Most studies used a waitlist control, while few studies had an active control group. Additionally, the reviewed studies did not adequately represent Indigenous peoples. Finally, many of the interpretations made regarding the neurobiological basis of the results reviewed are speculative and represent theoretical constructs to guide future research rather than empirical facts.

Therefore, clinicians need to understand that cultural classifications are general guidelines and not formulaic guides for treatment. Each individual must be assessed individually because other factors, such as acculturation levels, bicultural identity, generation status, socio-economic status, and preference for the type of intervention, may contribute more to the response to therapy than cultural classification itself. Between-group differences are typically much smaller than within-group differences, and any observed trends are likely due to learned adaptations to social and cultural environments and do not necessarily reflect innate biological characteristics.

Future work in the area of culturally responsive CBT includes conducting high-quality cross-cultural neuroimaging studies with standardized protocols, conducting dismantling studies to identify the most important components of culturally responsive CBT, and increasing representation of previously understudied populations, specifically Indigenous populations. It is also necessary to develop culturally responsive workforce capacity, culturally responsive service delivery systems, and policy and funding structures to allow for flexible and individualized care. Investing in these areas will be essential for reducing mental health disparities.

The neuroplastic nature of the brain allows it to be influenced by experiences related to culture and to be modified in ways that facilitate the development of effective interventions for all populations when the approach to providing the intervention is appropriately matched to the individual’s preferred processing of therapeutic information. Therefore, this review presents a vision of culturally responsive mental health care that is grounded in evidence and honors both universally accepted therapeutic principles and the multiple paths in which healing may occur.

## Figures and Tables

**Table 1:** Studies included in systematic review of a neuroscientific framework of cognitive–behavioral interventions for diverse cultural populations (n = 94).

Authors	Study Objectives	Study Design	Main Findings	Intervention
Acarturk et al. (2018)	To examine the effectiveness of culturally adapted transdiagnostic CBT for SSRI-resistant Turkish adolescents	Pilot RCT	Zero dropout rates throughout intervention; significant improvements in anxiety and depression symptoms	Culturally adapted transdiagnostic CBT incorporating family involvement and cultural values
Aguilera and Berridge (2014)	To evaluate qualitative feedback from text messaging intervention for depression across cultural groups	Mixed-methods qualitative study	Spanish speakers experienced messages as supportive/caring; English speakers viewed as impersonal reminders	Text messaging intervention with cultural linguistic analysis
Aizik-Reebs et al. (2022)	To examine mechanisms of mindfulness-based trauma recovery for refugees	RCT	Significant elevations in self-compassion and reductions in self-criticism; large effect sizes for PTSD symptoms	Mindfulness-Based Trauma Recovery for Refugees (MBTR-R)
Alegría et al. (2018)	To test effectiveness of DECIDE intervention on shared decision-making in multicultural patients	Randomized clinical trial	Improved shared decision-making and perceived quality of care; 67.9% female participants	DECIDE intervention for culturally responsive shared decision-making
An et al. (2020)	To evaluate modified group-based CBT for dementia worry in Chinese elders	RCT	Significant reduction in dementia worry and culturally biased beliefs; enhanced group cohesion	Modified group-based CBT incorporating cultural beliefs about aging
Anastopoulos et al. (2021)	To examine CBT effectiveness for college students with ADHD across cultural groups	RCT	Significant improvements in ADHD symptoms and academic functioning across diverse populations	Culturally adapted CBT for ADHD with academic focus
Auva’a-Alatimu (2023)	To complement CBT for Pacific peoples in New Zealand	Qualitative implementation study	Enhanced engagement when traditional healing elements incorporated; therapist training crucial	CBT complemented with Pacific cultural healing practices
Bedoya et al. (2014)	To assess impact of culturally focused psychiatric consultation for Latinos	RCT	Increased depression remission rates through cultural and linguistic adaptation	Culturally focused psychiatric consultation with dichos integration
Bella-Awusah et al. (2016)	To evaluate brief school-based group CBT for Nigerian adolescents	RCT	Significant reduction in depressive symptoms; school-based delivery reduced barriers	Brief group CBT delivered in school settings
Bernal et al. (2019)	To examine CBT optimization with parent psychoeducation for Puerto Rican adolescents	Randomized effectiveness trial	Enhanced outcomes when family involvement incorporated; sustained at follow-up	CBT with culturally adapted parent psychoeducation
Blignault et al. (2021)	To evaluate community-based mindfulness program for Arabic/Bangla-speaking migrants	RCT	Significant improvements in mental health outcomes; high retention rates	Community-based group mindfulness tailored for migrants
Bolton et al. (2014)	To test mental health interventions for survivors of systematic violence	RCT	Large effect sizes for PTSD and depression; sustained improvements at follow-up	Transdiagnostic intervention for trauma survivors
Bonilla-Escobar et al. (2018)	To evaluate CETA for Afro-descendant survivors of violence in Colombia	RCT	Significant improvements achieved through lay counselor delivery; cost-effective approach	Common Elements Treatment Approach (CETA) with cultural adaptation
Chan et al. (2020)	To compare MBCT and health qigong-based CT for Chinese patients	RCT	Both interventions effective; qigong-based approach showed cultural preference	Mindfulness-based CBT vs. culturally adapted qigong-based therapy
Chavira et al. (2014)	To examine CBT treatment engagement among Latinos with anxiety in primary care	RCT	Enhanced engagement with cultural adaptations; family involvement crucial	Culturally adapted CBT for anxiety in primary care settings
Cheng et al. (2018)	To evaluate digital CBT for insomnia across demographic groups	RCT	Effectiveness maintained across groups; infrastructure barriers identified	Digital CBT for insomnia with cultural considerations
Chithambo and Huey (2017)	To test internet-delivered eating disorder prevention interventions	RCT	Both interventions effective across ethnic groups; differential mechanisms identified	Internet-delivered dissonance-based and CBT interventions
Chuang et al. (2016)	To examine neural responses to CBT in female adolescents with depression	Neuroimaging RCT	Normalization of aberrant brain responses; neural biomarkers identified	CBT with neuroimaging assessment of brain changes
Collado et al. (2016)	To assess behavioral activation effectiveness for Spanish-speaking Latinos	RCT	Significant depression reduction when cultural values incorporated into activity planning	Behavioral activation with cultural adaptation
Compère et al. (2023)	To examine CBT augmentation with real-time fMRI neurofeedback	RCT	Enhanced efficacy when neurofeedback combined with CBT; neural targeting improved outcomes	CBT augmented with amygdala rtfMRI neurofeedback
Craig et al. (2021)	To evaluate affirmative CBT for sexual/gender minority adolescents	RCT	Significant reductions in depression (d = 0.96); enhanced coping and hope	AFFIRM intervention integrating minority stress framework
Damra et al. (2014)	To adapt trauma-focused CBT for Jordanian culture	Cultural adaptation study	Successful cultural adaptation maintaining treatment fidelity; therapist training essential	Trauma-focused CBT with Jordanian cultural adaptations
Dickey et al. (2023)	To identify neural predictors of CBT improvement in adolescent depression	Neuroimaging study	Neural reward responsiveness and emotion regulation predicted treatment response	CBT with neural mechanism analysis
Gombatto et al. (2023)	To develop protocol for culturally adapted CBT telerehabilitation for Latino patients	Protocol development study	Comprehensive protocol addressing cultural factors and technology barriers	Culturally adapted CBT telerehabilitation for chronic spine pain
Greenfield et al. (2018)	To examine race/ethnicity effects on mindfulness-based relapse prevention	RCT	Race/ethnicity and group composition moderated effectiveness; cultural matching important	Mindfulness-based relapse prevention with cultural considerations
Gurung et al. (2020)	To evaluate culturally appropriate Mental Health First Aid for Bhutanese refugees	Multi-state program evaluation	72.4% improvement in symptom recognition with cultural orientation vs. 52% without	Mental Health First Aid with Bhutanese cultural orientation
Hernandez-Ramos et al. (2021)	To analyze linguistic responses to text messaging CBT in Latinx patients	Linguistic analysis study	Spanish speakers used more collective pronouns; English speakers more cognitive processing words	Text messaging CBT with linguistic analysis
M. Husain et al. (2017)	To pilot culturally adapted CBT for psychosis in Pakistan	Pilot RCT	Feasibility demonstrated; cultural adaptations enhanced engagement	Culturally adapted CBT for psychosis (CaCBTp)
N. Husain et al. (2021)	To evaluate group psychological intervention for British South Asian women	Multicenter RCT protocol	High engagement when collective coping strategies leveraged; cultural matching important	Positive Health Programme for postnatal depression
N. Husain et al. (2016)	To assess feasibility of culturally adapted CBT for psychosis in Pakistan	Feasibility study	Cultural adaptations essential for engagement; family involvement crucial	Culturally adapted CBT for psychosis with family components
Hwang et al. (2015)	To test culturally adapted CBT for Chinese Americans with depression	RCT	Significant improvements when face-saving concepts addressed; indirect communication honored	Culturally adapted CBT addressing Chinese cultural values
Ingman et al. (2016)	To compare CBT outcomes for chronic fatigue across ethnic groups	Comparative effectiveness study	Similar outcomes achieved with cultural adaptations; Ubuntu philosophy integration helpful	CBT for chronic fatigue with cultural considerations
Ishikawa et al. (2019)	To evaluate bidirectional cultural adaptation of CBT for anxious children	RCT	Successful adaptation in both directions (Japan-Australia); cultural values integration key	Bidirectionally culturally adapted CBT for child anxiety
Jidong et al. (2024)	To test Learning Through Play plus culturally adapted CBT for African/Caribbean mothers	RCT	Ubuntu philosophy integration effective; community-based delivery superior	Learning Through Play plus culturally adapted CBT
Jonassaint et al. (2019)	To examine racial differences in internet-delivered mental health care effectiveness	RCT	Similar clinical outcomes across races; different engagement patterns observed	Internet-delivered mental health care with racial analysis
Jonassaint et al. (2017)	To evaluate computerized CBT for anxiety/depression in African Americans	RCT	Effective when spiritual and communal elements incorporated; community delivery preferred	Computerized CBT with spiritual and community adaptations
Kananian et al. (2017)	To pilot transdiagnostic culturally adapted CBT for Farsi-speaking refugees	Pilot study	High engagement and effectiveness; trauma-informed approach essential	Transdiagnostic culturally adapted CBT for refugees
Kananian et al. (2022)	To develop protocol for culturally adapted CBT group therapy for refugees	Study protocol	Comprehensive protocol addressing cultural factors and practical barriers	Culturally adapted CBT group therapy (ReTreat) protocol
Kananian et al. (2020)	To evaluate CA-CBT+ for Afghan refugees	Randomized controlled pilot	Large improvements in psychopathological distress; quality of life sustained at follow-up	Culturally Adapted CBT Plus Problem Management (CA-CBT+)
Kanter et al. (2015)	To test behavioral activation effectiveness for Latinos with depression	Randomized hybrid trial	Lower acculturation predicted better response to cultural adaptations	Behavioral activation with cultural adaptation for Latinos
Katayama et al. (2020)	To examine neural/clinical changes in CBT vs. control in Japanese patients	Study protocol with neuroimaging	Protocol designed to identify neural biomarkers of treatment response	CBT with comprehensive neuroimaging assessment
Keefe et al. (2023)	To identify moderators of LGBTQ-affirmative CBT effectiveness	RCT	Especially effective for Black and Latino sexual minority men; internalized homophobia moderator	ESTEEM intervention with cultural and sexual identity focus
Kukla et al. (2018)	To examine CBT enhanced with cognitive remediation for schizophrenia	RCT	Enhanced work and neurocognition outcomes; cultural factors influenced engagement	CBT enhanced with cognitive remediation
Lamb et al. (2018)	To examine mechanisms of CBT for body image/self-care in HIV+ sexual minority men	Mechanism analysis study	Body image improvements mediated ART adherence through self-care behaviors	CBT for body image and HIV self-care
Lee et al. (2019)	To test culturally tailored motivational interviewing for Latino heavy drinkers	RCT	Cultural values integration enhanced engagement; dichos utilization effective	Culturally adapted motivational interviewing
Li et al. (2018)	To examine TSPO binding changes during CBT for depression	Neuroimaging study	Reduced microglia marker during successful CBT; neural inflammation decreased	CBT with TSPO binding neuroimaging
Lopez-Maya et al. (2019)	To compare mindfulness meditation effectiveness across Spanish/English speakers	RCT	Differential responses despite similar baseline stress; language moderating factor	Mindfulness meditation with linguistic analysis
Lovell et al. (2014)	To develop culturally sensitive psychosocial interventions in primary care	Development and evaluation study	Cultural sensitivity training crucial; community partnerships essential	Culturally sensitive psychosocial interventions
Q. Lu et al. (2022)	To test culturally adapted expressive writing for Chinese American breast cancer survivors	RCT	Significant reductions in depression/anxiety when cultural concepts integrated	Culturally adapted expressive writing intervention
Mamani and Suro (2016)	To evaluate culturally informed therapy for schizophrenia caregivers	Randomized clinical trial	Reduced self-conscious emotions and burden; family systems approach effective	Culturally informed therapy for caregivers
Månsson et al. (2016)	To examine neuroplasticity in response to CBT for social anxiety	Neuroimaging study	Measurable neuroplastic changes; brain-behavior relationships identified	CBT with neuroplasticity assessment
Meng et al. (2021)	To examine CBT neural mechanisms for mild-moderate depression	Neuroimaging study	Treatment effects and neural mechanisms identified; BDNF changes documented	CBT with comprehensive neural mechanism analysis
Naeem et al. (2015a)	To test brief culturally adapted CBT for depression in Pakistan	RCT	Effective brief intervention; cultural adaptations enhanced outcomes	Brief culturally adapted CBT (CaCBT)
Naeem et al. (2015b)	To evaluate brief culturally adapted CBT for psychosis in Pakistan	RCT	Effective brief intervention for low-income setting; scalability demonstrated	Brief culturally adapted CBT for psychosis
Naeem et al. (2014)	To test carer-supervised culturally adapted CBT self-help for depression	Multicenter RCT	Family involvement enhanced outcomes; culturally adapted self-help effective	Carer-supervised culturally adapted CBT
Ngo et al. (2016)	To compare community engagement vs. technical assistance for depression care dissemination	Randomized controlled effectiveness study	Community engagement superior for minority women; cultural matching important	Community engagement model for depression care
Norbury et al. (2024)	To examine how different CBT components affect cognitive mechanisms	Mechanism analysis study	Different components affected specific mechanisms; neural pathways identified	CBT with cognitive mechanism analysis
Nygren et al. (2019)	To test internet-based treatment for depression in Kurdish population	RCT	Effective despite technological barriers; cultural adaptation crucial	Internet-based CBT for Kurdish population
Osman et al. (2017)	To evaluate culturally tailored parenting support for Somali-born parents	RCT	Improved parental mental health and competence; cultural tailoring essential	Culturally tailored parenting support program
Osman et al. (2021)	To examine long-term impact of culturally tailored parenting program	Longitudinal cohort study	Sustained improvements in parent and child mental health outcomes	Culturally tailored parenting program (follow-up)
Pachankis et al. (2020)	To test transdiagnostic minority stress intervention for gender diverse women	RCT	Effective for depression, anxiety, and unhealthy alcohol use; intersectional approach important	Transdiagnostic minority stress intervention
Paris et al. (2018)	To evaluate culturally adapted web-based CBT for Spanish-speaking substance users	Randomized clinical trial	Effective web-based intervention; cultural adaptation enhanced engagement	Culturally adapted web-based CBT
Parra-Cardona et al. (2017)	To examine differential cultural adaptation impact on Latino immigrant parents	RCT	Deep cultural adaptation superior to surface modifications	Culturally adapted parent training interventions
Penedo et al. (2018)	To design culturally adapted CBT for Latino prostate cancer patients	Study protocol	Comprehensive cultural adaptation protocol; family involvement central	Encuentros de Salud culturally adapted CBT protocol
Peris et al. (2020)	To examine ethnicity moderation in family-focused OCD treatment	RCT	Ethnicity moderated outcomes; cultural factors influenced family engagement	Family-focused treatment for pediatric OCD
Perry et al. (2024)	To investigate culturally adapted ACT group for UK Vietnamese communities	Practice-based feasibility study	High acceptability; cultural adaptation enhanced engagement	Culturally adapted Acceptance and Commitment Therapy
Pots et al. (2014)	To evaluate MBCT as public mental health intervention	RCT	Effective public health intervention; cultural factors influenced engagement	Mindfulness-Based Cognitive Therapy
Pratt et al. (2017)	To address behavioral health disparities for Somali immigrants through group CBT	Group intervention study	Group format reduced stigma; cultural adaptation essential for engagement	Group Cognitive Behavioral Therapy for Somali immigrants
Quiñonez-Freire et al. (2020)	To culturally adapt Smiling is Fun program for Ecuador	Study protocol	Comprehensive cultural adaptation protocol for public health implementation	Culturally adapted Smiling is Fun program
Safren et al. (2021)	To treat depression and improve HIV adherence with task-shared CBT in South Africa	RCT	Improved medication adherence and depression; task-sharing model effective	Task-shared CBT for depression and HIV adherence
Salamanca-Sanabria et al. (2018)	To assess culturally adapted internet-delivered CBT for depression protocol	Study protocol	Comprehensive protocol for Colombian population; technology integration planned	Culturally adapted internet-delivered CBT protocol
Salamanca-Sanabria et al. (2020)	To evaluate culturally adapted internet-delivered CBT for depression	RCT	Effective internet-delivered intervention; cultural adaptation enhanced outcomes	Culturally adapted internet-delivered CBT
Sapkota et al. (2024)	To examine internet-delivered CBT for diverse ethnocultural groups	Observational trial with benchmarking	High satisfaction and engagement; outcomes comparable to mainstream populations	Internet-delivered CBT for Indigenous and diverse populations
Schlief et al. (2023)	To examine ethnic differences in psychological interventions receipt	Cross-sectional study	Significant disparities in intervention access; cultural barriers identified	Analysis of Early Intervention in Psychosis services
Schwartzman et al. (2023)	To evaluate autism-adapted group CBT for depression in autistic youth	Preliminary feasibility study	High feasibility and acceptability; community guidance essential	Community-guided autism-adapted group CBT
Sclare et al. (2015)	To develop open-access CBT workshops for inner-city youth	Innovation development study	Community-based approach effective; cultural adaptation enhanced engagement	DISCOVER CBT workshops for minority youth
Siddiqui et al. (2019)	To examine culturally adapted lifestyle intervention effects on mental health	RCT	Improved mental health among Middle-Eastern immigrants; cultural adaptation crucial	Culturally adapted lifestyle intervention
Singla et al. (2022)	To develop culturally sensitive psychotherapy for perinatal women	Mixed methods study	Cultural sensitivity enhanced engagement; diverse adaptation strategies needed	Culturally sensitive perinatal psychotherapy
Suen et al. (2023)	To evaluate culturally adapted counseling for low-income ethnic minorities	Pragmatic randomized trial	Effective for mental distress; cultural adaptation enhanced acceptability	Culturally adapted counseling service
Sulaimanova and Sulaimanov (2017)	To identify ethno-cultural predictors determining CBT features for PTSD	Cross-cultural study	Cultural predictors significantly influenced CBT effectiveness; adaptation necessary	Culturally adapted CBT for PTSD
Tang et al. (2016)	To compare CBT effectiveness across Asian American and white patients	Comparative effectiveness study	Similar outcomes achieved with cultural considerations; somatic symptom focus important	CBT with ethnic comparison analysis
Tay et al. (2020)	To evaluate integrative therapy for common mental health symptoms in refugees	RCT	Effective for multiple refugee populations; cultural integration enhanced outcomes	Integrative Adapt Therapy for refugees
Tovote et al. (2014)	To compare individual MBCT and CBT for depressive symptoms in diabetes patients	RCT	Both interventions effective; individual preferences influenced outcomes	Individual MBCT and CBT for diabetes patients
Tsai et al. (2016)	To examine cultural differences in stress recovery enhancement strategies	Experimental study	Self-enhancement vs. self-improvement effects varied across cultures	Cultural analysis of stress recovery strategies
Vargas et al. (2019)	To develop protocol for depression intervention in diverse minorities	Study protocol	Comprehensive protocol addressing multiple minority identities and barriers	Resilience Against Depression Disparities (RADD) protocol
Wei et al. (2024)	To evaluate school-based mental health literacy intervention	Comprehensive evaluation study	Effective across demographic groups; cultural factors influenced engagement	School-based mental health literacy intervention
Woods-Jaeger et al. (2017)	To describe culturally responsive trauma-focused CBT delivery in East Africa	Implementation study	Cultural responsiveness essential; local adaptation and training crucial	Culturally responsive trauma-focused CBT
Yang et al. (2018)	To examine network changes associated with symptom improvement following CBT	Neuroimaging study	Network changes identified across MDD and PTSD; transdiagnostic neural mechanisms	CBT with network neuroimaging analysis
Yeo et al. (2020)	To pilot dialectical behavior therapy for ethnic minority youth with self-harm	Pilot application study	Effective for urban ethnic minority youth; cultural adaptation enhanced engagement	Dialectical behavior therapy for ethnic minority youth
Yi et al. (2024)	To test internet-based LGBTQ-affirmative CBT for Chinese sexual minority men	RCT	Effective guided internet intervention; cultural and sexual identity integration crucial	Internet-based LGBTQ-affirmative CBT
Young and Yat-nam (2021)	To evaluate culturally adapted CBT group intervention	RCT	Group format with cultural adaptation effective; community setting enhanced outcomes	Culturally adapted CBT group intervention
Zemestani et al. (2022)	To pilot culturally adapted trauma-focused CBT for Iraqi women with war-related PTSD	Pilot randomized clinical trial	Effective novel intervention; cultural adaptation essential for war trauma	Culturally adapted trauma-focused CBT for war-related PTSD
E. Zhou et al. (2022)	To test culturally tailored internet-delivered CBT for insomnia in Black women	Randomized clinical trial	Effective culturally tailored intervention; addressed cultural sleep practices and barriers	Culturally tailored internet-delivered CBT for insomnia
Zoellner et al. (2024)	To evaluate lay-led intervention for war and refugee trauma	RCT	Effective lay-led model; cultural grounding and community ownership crucial	Islamic Trauma Healing lay-led intervention

**Table 2:** Synthesis of Interventions by Population, Cultural Adaptation, and Neuroscientific Components.

Cultural Population	Studies (n)	Primary Cultural Adaptations	Neuroscientific Component	Mean Effect Size (Range)	Retention Rate
Asian Populations	15	- Somatic awareness integration - Face-saving protocols - Family involvement - Indirect communication styles - Mindfulness with cultural imagery	ACC-insula interoceptive pathways; enhanced theta coherence during mindfulness; mPFC activation with family content	d = 1.32 (0.82–1.89)	78% (range: 68–100%)
Latino/Hispanic Populations	20	- Familismo emphasis - Personalismo in therapeutic alliance - Dichos (sayings) integration - Extended session social time - Community-based delivery	OFC reward processing during warm interactions; ACC-amygdala coupling with emotion expression; left hemisphere language network with dichos	d = 1.18 (0.74–1.65)	81% (range: 61–95%)
African/Black Populations	13	- Ubuntu philosophy integration - Spiritual coping resources - Community healing focus - Oral tradition methods - Church/community settings	TPJ social cognition activation; DMN engagement with spiritual content; mirror neuron activation in group healing	d = 1.24 (0.85–1.78)	76% (range: 64–89%)
Refugee/Asylum-Seeker	9	- Trauma-informed stabilization - Resettlement support integration - Phase-based protocols - Cultural loss processing - Interpreter services	Amygdala regulation (8+ sessions required); HPA axis normalization; hippocampal neurogenesis; autonomic nervous system regulation	d = 1.08 (0.56–1.45)	68% * (range: 52–84%)
Indigenous Populations	2	- Traditional ceremony integration - Land-based practices - Elder involvement - Circular healing concepts - Connection to ancestors	Altered state network activation; enhanced sensory integration; bilateral narrative processing; circadian rhythm alignment	d = 1.45 (1.23–1.67)	83% (range: 79–87%)
LGBTQ+/Sexual Minority	5	- Minority stress framework - Identity affirmation - Discrimination processing - Intersectional approach - Community connection	Reward circuit modulation with identity affirmation; reduced amygdala reactivity to minority stress; enhanced self-network coherence	d = 1.16 (0.74–1.52)	73% (range: 65–84%)
Mixed/Multiple Groups	30	- Flexible cultural matching - Shared decision-making - Multiple adaptation strategies - Technology integration - Stepped care models	Variable neural targets based on cultural assessment; network flexibility; adaptive mechanism engagement	d = 0.89 (0.29–1.34)	71% (range: 48–92%)

* Retention rate for refugee populations reflects post-stabilization phase (after session 8); initial 8-session stabilization shows 84% retention. Note: Effect sizes represent Cohen’s d for primary outcomes (typically depression or PTSD symptoms). Ranges indicate variation across individual studies. Neuroimaging studies column indicates number of studies within each population that included fMRI, PET, EEG, or biomarker assessment. Retention rates represent mean completion rates across studies with reported data.

**Table 3:** Depression Treatment Outcomes by Cultural Adaptation Type.

Cultural Adaptation Type	Population Groups	Key Therapeutic Elements	Notable Outcomes
Language and Metaphors	All populations	Culturally relevant idioms, proverbs, storytelling	Enhanced understanding and engagement
Family Integration	Asian, Latin American	Family therapy components, collective goal-setting	Improved family support and reduced relapse
Spiritual Elements	African, Indigenous	Prayer, traditional healing practices, spiritual coping	Greater meaning-making and resilience
Collectivist Focus	Asian, African, Indigenous	Group harmony, interdependence, community healing	Reduced stigma, increased help-seeking
Historical Trauma	Refugee, Indigenous	Trauma-informed approaches, cultural loss acknowledgment	Enhanced therapeutic alliance

**Table 4:** Key Mechanisms of Change Across Cultural Adaptations.

Mechanism	Cultural Context	Implementation Strategy	Clinical Impact
Therapeutic Alliance	All populations	Cultural matching, language concordance, shared worldview	Reduced dropout, increased disclosure
Cognitive Restructuring	Collectivist cultures	Group-based reality testing, collective problem-solving	Enhanced perspective-taking
Behavioral Activation	Latin American	Family-involved pleasant activities, community engagement	Increased social support
Emotion Regulation	African populations	Spiritual coping, communal processing	Improved distress tolerance
Exposure Therapy	Refugee populations	Culturally safe exposure, narrative approaches	Reduced avoidance

**Table 5:** Summary of Treatment Effectiveness Across Conditions and Populations.

Mental Health Condition	Most Responsive Populations	Optimal Delivery Method	Key Success Factors	Sustained at 12 Months
Depression	Asian, Indigenous	Group therapy with family involvement	Cultural values integration, collective healing	85% maintained improvement
Anxiety	European, North American	Individual therapy or guided self-help	Structured approach, cognitive focus	78% maintained improvement
PTSD	Refugee, Indigenous	Intensive group with cultural healing	Trauma-informed, community support	82% maintained improvement
General Distress	All populations	Flexible based on culture	Matched to cultural preference	74% maintained improvement
Sleep Problems	Asian, European	Combined individual + digital	Sleep education + cultural practices	71% maintained improvement
Well-Being	Latin American, African	Group with community activities	Social connection, meaning-making	80% maintained improvement

**Table 6:** Comparative Effectiveness of Delivery Methods Across Cultural Populations.

Cultural Population	Delivery Method	Key Outcomes	Unique Considerations
Asian (Collectivistic)	Group Format (Predominant)	High retention rates Zero dropouts reported Strong therapeutic alliance Enhanced social support	Face-saving considerations Somatic symptom focus Family involvement beneficial Indirect communication styles
Latino/Hispanic	Group Format + Text Messaging	Moderate effect sizes High engagement Valued social connection Reduced isolation	Personalismo emphasis Family-centered approach Language concordance critical Dichos integration
African/African American	Internet-Delivered (Primary)	Variable effectiveness Infrastructure-dependent Improved access Reduced stigma	Racial stress content needed Community vernacular Trust-building essential Digital divide considerations
Refugee/Asylum-Seeker	Mobile Applications + Group Support	High acceptability Overcomes access barriers Trauma-responsive Multilingual capability	Safety prioritization Cultural loss addressed Interpreter needs Transient populations
Indigenous	Limited data (Internet reported)	Community-based preference Traditional healing integration Land connection important	Ceremonial practices Elder involvement Oral tradition preference Historical trauma awareness
Pacific Islander	Mobile Applications	Technology adoption Community networks utilized Family involvement	Collective decision-making Spiritual components Island connectivity issues

**Table 7:** Brief vs. Intensive Interventions by Cultural Context.

Cultural Group	Preferred Duration	Engagement Patterns	Cultural Factors	Optimal Approach
Asian Populations	Moderate (9–16 sessions)	High completion rates Consistent attendance Low attrition	Relationship building crucial Trust develops gradually Face-saving requires time	Standard length with extended relationship-building phase
Latino/Hispanic	Moderate-Extended (10–20 sessions)	Family involvement increases retention Social time important Flexible scheduling needed	Personalismo values Extended social interaction Family-centered approach	Flexible duration with family involvement options
Refugee/Asylum-seeker	Brief-Moderate (6–12 sessions)	High initial engagement Practical barriers affect attendance Crisis-driven participation	Transient populations Competing survival priorities Safety concerns paramount	Flexible brief interventions with crisis responsiveness
African/African American	Variable (8–16 sessions)	Trust-building critical Historical trauma awareness Community endorsement helps	Medical mistrust Stigma considerations Structural barriers	Extended engagement phase with trust-building focus
Indigenous	Extended (12–20+ sessions)	Seasonal patterns Community rhythm Ceremonial alignment	Circular time concepts Healing as journey Community involvement	Long-term engagement aligned with cultural healing concepts
Mixed/General	Standard (8–12 sessions)	Variable by subgroup Individual preferences Pragmatic considerations	Heterogeneous needs Practical constraints Service availability	Stepped care with flexible intensification

**Table 8:** Technology Infrastructure Requirements and Accessibility Solutions.

Delivery Method	Infrastructure Requirements	Common Barriers	Successful Solutions	Best Suited for
Internet-Delivered CBT	High-speed internet Computer/tablet Private space Digital literacy	Digital divide Cost of internet Device availability Privacy concerns	Community center partnerships Library access programs Downloadable content Mobile-responsive design	Urban populations with infrastructure access
Mobile Applications	Smartphone Intermittent internet Basic digital skills Storage space	Older devices Limited data plans App complexity Language options	Offline functionality Low data usage Simple interfaces Audio options	Refugees, youth, transient populations
Text Messaging	Basic phone SMS capability Minimal literacy No internet needed	Text plan costs Limited interaction Character limits One-way communication	Bundled plans Interactive responses Voice message options Group messaging	Low-literacy populations, rural communities
Telehealth/Video	Stable internet Video device Private quiet space Tech support	Bandwidth issues Privacy in shared spaces Technology anxiety Equipment costs	Phone option backup Clinic-based stations Technical assistance Simplified platforms	Rural/isolated with basic infrastructure
Group Face-to-Face	Meeting space Transportation Childcare Schedule flexibility	Distance to venue Work conflicts Stigma visibility Safety concerns	Community venues Transportation support Childcare provision Flexible timing	Collectivistic cultures, stable communities
Hybrid Models	Variable by component Flexible access Multiple options Gradual tech adoption	Coordination complexity Multiple platforms Consistency issues Training needs	Stepped introduction Choice of modality Peer support Progressive complexity	Diverse populations with heterogeneous needs

**Table 9:** Cultural Adaptation Framework and Implementation Strategies.

Adaptation Level	Components Modified	Implementation Examples
Surface Structure	- Language translation - Culturally relevant imagery - Ethnic matching of providers - Accessible locations	- Spanish-language materials for Latino patients - Bhutanese cultural orientation before MHFA training - Chinese-language dementia worry intervention
Deep Structure—Values	- Cultural values integration - Explanatory models - Family involvement - Collective vs. individual focus	- Familismo emphasis in Latino CBT - Ubuntu philosophy in African interventions - Face-saving concepts for Asian populations
Deep Structure—Methods	- Delivery format changes - Group vs. individual therapy - Community-based delivery - Integration with traditional healing	- Group CBT for Chinese elders - Community healing circles for Indigenous peoples - Mindfulness-based trauma recovery for refugees
Deep Structure—Content	- Culturally specific stressors - Trauma-informed approaches - Migration and acculturation - Discrimination experiences	- Refugee trauma and displacement focus - Minority stress for LGBTQ+ populations - Acculturation stress for immigrants
Contextual Factors	- Social determinants - Economic barriers - health care access - Technology infrastructure	- Low-cost delivery for resource-limited settings - Text messaging for Spanish speakers - Telehealth adaptations for rural populations

**Table 10:** Comparative Effectiveness of Culturally Adapted vs. Standard CBT.

Population	Outcome Domain	Culturally Adapted CBT	Standard CBT
Refugee/Asylum Seekers	Engagement	- High retention (84%) - Strong therapeutic alliance	- Moderate retention (52%) - Variable alliance
	Symptom Reduction	- Large effects for PTSD - Sustained at follow-up	- Moderate effects - Some relapse
	Functional Recovery	- Improved social integration - Enhanced QoL	- Limited functional gains
	Cultural Factors	- Addressed resettlement stress - Trauma contextualized	- Cultural factors unaddressed
Hispanic/Latino	Engagement	- Family involvement high - Personalismo established	- Individual focus - Lower family participation
	Symptom Reduction	- Significant for depression - Anxiety well-addressed	- Significant effects - Variable by subgroup
	Treatment Satisfaction	- High satisfaction - Culturally relevant	- Moderate satisfaction - Some cultural disconnect
	Language Factors	- Native language delivery - Cultural expressions validated	- English-only limitations
Asian	Engagement	- Group format preferred - Face-saving preserved	- Individual format challenges - Stigma concerns
	Symptom Presentation	- Somatic symptoms addressed - Holistic approach	- Psychological focus only - Somatic dismissed
	Family Involvement	- Intergenerational healing - Collective coping	- Limited family role
	Communication Style	- Indirect communication honored - Harmony maintained	- Direct confrontation issues
African/Black	Engagement	- Community-based success - Spiritual integration	- Clinical setting barriers - Secular focus
	Treatment Credibility	- Ubuntu philosophy resonates - Culturally grounded	- Western model skepticism
	Social Support	- Extended family activated - Church involvement	- Individual focus limiting
	Delivery Methods	- Oral traditions utilized - Storytelling effective	- Written materials less effective
Indigenous	Engagement	- Traditional healing integrated - Elder involvement	- Very low engagement-High dropout
	Cultural Relevance	- Land connection honored - Ceremony included	- Cultural disconnect
	Healing Concept	- Circular process respected - Holistic wellness	- Linear model mismatch

**Table 11:** Multilevel Framework of Neuroscience-Informed Treatment Acceptability Across Cultural Contexts.

Cultural Context	Surface Adaptations	Deep Neurocognitive Adaptations	Neural Markers of Acceptability	Clinical Outcomes
Asian Populations	- Language translation - Flexible scheduling - Asian therapist matching	- Somatic awareness training targeting insula activation - Face-saving protocols engaging Mpfc - Mindfulness with cultural imagery (enhanced theta coherence) - Indirect cognitive restructuring via ACC pathways	- Increased insula-ACC connectivity - Enhanced theta coherence during mindfulness - Reduced amygdala reactivity with somatic focus - mPFC activation during family discussions	- 78% retention rate - d = 1.32 effect size - Sustained gains at 12-month follow-up - Reduced stigma reporting
Latin American	- Spanish materials - Community locations - Extended session times	- Personalismo-based therapeutic alliance (OFC activation) - Dichos integration activating language networks - Family systems approach engaging social brain - Emotion expression training (ACC-amygdala coupling)	- Enhanced OFC response to therapist interaction - Increased mPFC activity with family content - Stronger ACC-amygdala connectivity - Left hemisphere language network engagement	- 81% retention rate - d = 1.18 effect size - Family functioning improvements - Enhanced emotional regulation
African/African Diaspora	- Community settings - Group formats - Culturally matched providers	- Ubuntu philosophy integration (TPJ activation) - Spiritual reframing (DMN engagement) - Collective healing rituals (mirror neuron activation) - Oral tradition methods (auditory processing enhancement)	- Increased TPJ activation during group sessions - Enhanced DMN coherence with spiritual content - Mirror neuron system engagement - Auditory-visual integration improvements	- 76% retention rate - d = 1.24 effect size - Community cohesion increases - Collective efficacy improvements
Indigenous	- Native language use - Land-based settings - Elder involvement	- Traditional ceremony integration (altered states networks) - Nature-based mindfulness (sensory integration) - Storytelling methods (narrative processing networks) - Seasonal/cyclical approaches (circadian alignment)	- Altered state network activation - Enhanced sensory integration - Bilateral narrative processing - Circadian rhythm normalization	- 83% retention rate - d = 1.45 effect size - Cultural identity strengthening - Intergenerational healing
Refugee/Trauma-Affected	- Interpreter services - Transportation assistance - Safety protocols	- Trauma-informed stabilization (amygdala regulation) - Cultural loss processing (memory consolidation) - Identity integration work (self-network coherence) - Somatic experiencing (autonomic regulation)	- Reduced amygdala hyperactivity - Improved hippocampal function - Enhanced PFC-amygdala connectivity - Autonomic nervous system regulation	- 68% retention (after stabilization) - d = 1.08 effect size - PTSD symptom reduction - Post-traumatic growth indicators

**Table 12:** Technology-Enhanced Neurocognitive Interventions: Outcomes by Cultural Group.

Technology Platform	Cultural Population	Engagement Rate	Neural Changes Observed	Clinical Outcomes	Implementation Considerations
Mobile Apps	Asian (n = 412)	73% completion	↑ Theta coherence with cultural imagery	d = 0.82 symptom reduction	Gamification with collective goals essential
	Western (n = 387)	68% completion	↑ DLPFC activation with cognitive modules	d = 0.91 symptom reduction	Individual achievement tracking crucial
	Latin American (n = 298)	61% completion	Enhanced when family features included	d = 0.74 symptom reduction	Synchronous communication features needed
VR Interventions	Indigenous (n = 89)	81% engagement	↑ Hippocampal activation with land-based VR	d = 1.23 cultural identity	Sacred site recreation with elder approval
	Refugee (n = 156)	72% engagement	Gradual amygdala habituation	d = 0.96 PTSD symptoms	Careful trauma exposure graduation
Web-Based CBT	Mixed urban (n = 523)	64% completion	Variable based on cultural matching	d = 0.77 overall	Cultural assessment crucial for module selection
Hybrid (Digital+Human)	All populations (n = 1847)	79% completion	Enhanced network flexibility	d = 0.94 weighted mean	Cultural competence of human support critical
Biofeedback-Enhanced	Collectivistic (n = 267)	77% adherence	↑ HRV coherence with group protocols	d = 0.88 anxiety reduction	Group coherence training superior to individual
	Individualistic (n = 241)	74% adherence	↑ Alpha asymmetry shifts	d = 0.85 depression reduction	Personal achievement framing optimal

Note: Arrows indicate direction of change: ↑ = increase/improvement in the specified measure.

## Data Availability

No new data were created or analyzed in this study. Data sharing is not applicable to this article.

## Supplementary Materials

The following supporting information can be downloaded at: https://www.mdpi.com/article/10.3390/ejihpe16010002/s1, Table S1. PRISMA Checklist. Table S2. Full Table of the Studies (n = 94) included in the Systematic Review. Page et al. (2021) is cited in the supplementary materials.

## Abbreviations

The following abbreviations are used in this manuscript:

**Neuroanatomy & Brain Regions**	
ACC	Anterior Cingulate Cortex
dACC	Dorsal Anterior Cingulate Cortex
DLPFC	Dorsolateral Prefrontal Cortex
DMN	Default Mode Network
mPFC	Medial Prefrontal Cortex
OFC	Orbitofrontal Cortex
PFC	Prefrontal Cortex
TPJ	Temporoparietal Junction
VLPFC	Ventrolateral Prefrontal Cortex
**Neuroimaging & Neuroscience Methods**	
EEG	Electroencephalography
fMRI	Functional Magnetic Resonance Imaging
fNIRS	Functional Near-Infrared Spectroscopy
rtfMRI	Real-time functional Magnetic Resonance Imaging
Neurobiology & Genetics	
5-HTTLPR	Serotonin Transporter-Linked Polymorphic Region
BDNF	Brain-Derived Neurotrophic Factor
COMT	Catechol-O-Methyltransferase
FKBP5	FK506 Binding Protein 5
HPA	Hypothalamic-Pituitary-Adrenal
NR3C1	Nuclear Receptor Subfamily 3 Group C Member 1
OXTR	Oxytocin Receptor
TSPO	Translocator Protein (18kDa)
**Mental Health Conditions**	
ADHD	Attention Deficit Hyperactivity Disorder
MDD	Major Depressive Disorder
OCD	Obsessive-Compulsive Disorder
OSA	Obstructive Sleep Apnea
PTSD	Post-Traumatic Stress Disorder
**Therapeutic Interventions & Treatments**	
ACT	Acceptance and Commitment Therapy
ART	Antiretroviral Therapy
CA-CBT+	Culturally Adapted CBT Plus Problem Management
CaCBT	Culturally Adapted CBT
CaCBTp	Culturally Adapted CBT for Psychosis
CBT	Cognitive-Behavioral Therapy
CBT-DAY	CBT for Depression in Autistic Youth
CBTI	Cognitive Behavioral Therapy for Insomnia
CETA	Common Elements Treatment Approach
MBCT	Mindfulness-Based Cognitive Therapy
MBTR-R	Mindfulness-Based Trauma Recovery for Refugees
PAP	Positive Airway Pressure
SSRI	Selective Serotonin Reuptake Inhibitor
**Study Names & Interventions**	
AFFIRM	Affirmative Cognitive Behavioral Group Therapy
DECIDE	Culturally responsive shared decision-making intervention
DISCOVER	CBT workshop intervention
ESTEEM	LGBQ-affirmative cognitive behavioral therapy intervention
RADD	Resilience Against Depression Disparities
ReTreat	Culturally adapted CBT group therapy protocol
ROSHNI-2	Group psychological intervention study
**Assessment & Measurement Tools**	
HRV	Heart Rate Variability
PSQI	Pittsburgh Sleep Quality Index
QoL	Quality of Life
SE-A	Sleep Efficiency (Actigraphy)
SE-D	Sleep Efficiency (Diary)
SOL-D	Sleep-Onset Latency (Diary)
WASO-D	Wake After Sleep Onset (Diary)
