# Supplementary material for: Neuroscientific Framework of Cognitive–Behavioral Interventions for Mental Health Across Diverse Cultural Populations: A Systematic Review of Effectiveness, Delivery Methods, and Engagement
Source: Eur J Investig Health Psychol Educ. 2025 Dec 22;16(1):2. doi: 10.3390/ejihpe16010002 (PMC12839909; doi:10.3390/ejihpe16010002)
Supplement: Supplementary file 1 [file ejihpe-16-00002-s001.zip › Table_S2.pdf]

Table S2: Complete Systematic Review of Neuroscience-Informed Cognitive-Behavioral Interventions for Diverse Cultural Populations (n=94)

Authors	Methodology	Population Characteristics	Intervention Effects	Experimental Techniques	Intervention	Outcome Measured
Acarturk et al. (2018)	Participants: 13 treatment-resistant Turkish adolescents with anxiety or mood disorders. Intervention: 10 weekly 90-minute group CA-CBT sessions. Design: Single-arm pilot study with pre-post-follow-up design.	Turkish adolescents aged 13-18 years with anxiety or mood disorders; treatment-resistant to SSRI medication; recruited from child psychiatry clinic	Depression (BDI): post-treatment d=0.9, follow-up d=1.0; Anxiety (SCARED): post-treatment d=0.8, follow-up d=0.9; Zero dropout rate throughout intervention period	Group therapy sessions; Screen for Childhood Anxiety Related Disorders (SCARED); Beck Depression Inventory; Turkish cultural adaptation including family involvement	Culturally adapted transdiagnostic CBT (CA-CBT) in group format, 10 sessions over 10 weeks, incorporating Turkish cultural values and family dynamics	Screen for Childhood Anxiety Related Disorders (SCARED); Beck Depression Inventory; Treatment engagement and retention measures
Aguilera and Berridge (2014)	Daily text messages sent as adjunct to group CBT for depression. Qualitative analysis of participant feedback through semi-structured interviews	Adult men and women aged 23-72; majority female (13 out of total participants); English and Spanish speakers recruited from community mental health clinic	Increased self-awareness (55% of participants); Cultural differences: Spanish speakers experienced messages as supportive/caring, English speakers as self-monitoring tools	Daily text messaging system; Qualitative interviews; Cross-linguistic analysis of responses	Daily text messages as adjunct to group Cognitive Behavioral Therapy for depression, culturally adapted for Spanish and English speakers	Qualitative feedback measures: self-reflection, feeling cared for and supported, appreciation for convenience, self-awareness enhancement
Aizik-Reebs et al. (2022)	Randomized wait-list control trial focusing on	Traumatized and chronically stressed asylum-seekers; 46%	Self-compassion: Significant elevation in MBTR-	Self-Referential Encoding Task (SRET); Diffusion modeling; Computational	Mindfulness-Based Trauma Recovery for Refugees	Self-compassion, self-criticism, depression (PHQ-

	adaptive and maladaptive forms of self-referential processing. 8-week intervention with pre-post-follow-up assessments	female; Urban post-migration environment in Israel; Multiple countries of origin	R vs wait-list (d=0.72, p<0.001); Self-criticism: Significant reduction (d=-0.58, p<0.01); PTSD symptoms: Large effect size improvements	modeling of attentional bias; Neuropsychological assessment battery	(MBTR-R): mindfulness and compassion-based intervention adapted for refugee trauma experiences	9), PTSD symptoms (PCL-5), quality of life measures
Alegría et al. (2018)	Cross-level 2×2 randomized clinical trial with clinicians and patients randomly selected. Multi-site implementation across community mental health centers	Ethnically/racially diverse sample; 67.9% female participants; Mean age 44.0 years; Primary diagnoses: depression, anxiety, PTSD; Community mental health setting	Shared Decision Making: Clinician intervention showed significant improvements in SDM scores; Patient intervention enhanced perceived quality of care; Combined interventions showed additive effects	Clinician workshop training; Coaching telephone calls (up to 6 sessions); 3-session patient activation intervention; Audio-recorded clinical encounters	DECIDE intervention: Clinician workshop and coaching calls; Patient activation sessions focusing on shared decision-making in culturally diverse contexts	Shared decision making (SDM) measures, patient-perceived quality of care, treatment engagement, clinical outcomes
An et al. (2020)	Participants recruited from four elder group homes, randomly assigned to intervention or control. 8-week group intervention with pre-post-follow-up design	Chinese elders; Older adults residing in elder group homes; Mean age 74.3 years; Mild cognitive concerns but no diagnosed dementia	Dementia worry: Intervention g=-1.52, 95% CI (-2.01, -1.03), p<0.001; Culturally biased beliefs: Significant reduction; Group cohesion: Enhanced throughout intervention	Modified group cognitive behavioral therapy; Eight weekly 60-minute face-to-face group sessions; Cultural adaptation for Chinese elderly population	Modified group CBT delivered in group setting, specifically adapted for Chinese cultural beliefs about aging and dementia	Dementia worry scales, culturally biased beliefs about dementia, group cohesion measures, cognitive screening assessments

Anastopoulos et al. (2021)	Randomized controlled trial comparing CBT to delayed treatment control. 12-week individual CBT protocol adapted for college students with ADHD	College students with ADHD; Diverse ethnic representation; Mean age 20.1 years; Full-time undergraduate students; Confirmed ADHD diagnosis	ADHD symptoms: Significant improvements (d=0.87); Academic functioning: Enhanced study skills and time management; Executive functioning: Moderate improvements in daily living skills	Individual CBT sessions; ADHD symptom rating scales; Academic performance measures; Executive function assessments	Cognitive-behavioral therapy adapted for college students with ADHD, focusing on academic skills, time management, and executive functioning	ADHD symptom severity, academic performance measures, executive functioning scales, quality of life assessments
Auva'a-Alatimu (2023)	Qualitative study exploring complementary approaches to CBT for Pacific peoples in New Zealand. Community-based participatory research methodology	Pacific peoples living in New Zealand; Community-recruited sample; Diverse Pacific Island origins; Range of mental health conditions	Enhanced engagement when traditional healing elements incorporated; Improved therapeutic alliance; Greater cultural acceptability and treatment satisfaction	Community consultation; Traditional healing integration; Therapist cultural competency training; Qualitative interviews	CBT complemented with Pacific cultural healing practices, traditional spiritual elements, and community-based delivery approaches	Treatment engagement, cultural acceptability, therapeutic alliance, symptom improvement, community integration measures
Bedoya et al. (2014)	Randomized controlled trial comparing culturally focused psychiatric consultation to treatment as usual in primary care setting	Latino primary care patients with depression; Spanish-speaking; Mean age 47.2 years; Low-income community health center patients	Depression remission: 65% in intervention vs 35% in control; Significant improvement in depressive symptoms; Enhanced	Culturally focused psychiatric consultation; Integration of dichos (cultural sayings) and metaphors; Collaborative care model implementation	Culturally focused psychiatric consultation incorporating Latino cultural values, dichos, and adapted therapeutic concepts for primary care	Depression severity (PHQ-9), treatment engagement, patient satisfaction, cultural congruence measures

			treatment engagement and satisfaction			
Bella-Awusah et al. (2016)	School-based randomized controlled trial with wait-list control. 5 weekly group sessions delivered in school setting	Nigerian secondary school adolescents; Ages 14-18 years; Urban school setting; Students identified with depressive symptoms	Significant reduction in depressive symptoms (d=0.82); Improved school attendance; Enhanced peer relationships; Sustained improvements at 3-month follow-up	Brief school-based group CBT; Five weekly 60-minute group sessions; Peer support integration; School counselor delivery	Brief group CBT delivered in school settings, adapted for Nigerian cultural context and addressing adolescent developmental needs	Depression severity scales, school attendance records, peer relationship measures, academic performance indicators
Bernal et al. (2019)	Randomized effectiveness trial comparing CBT with and without parent psychoeducation. Community-based implementation in Puerto Rico	Puerto Rican adolescents with major depression; Mean age 15.2 years; Community mental health setting; Family involvement emphasized	CBT with parent psychoeducation showed superior outcomes; Depression remission: 78% vs 61% for CBT alone; Family functioning improvements	Individual CBT sessions; Parent psychoeducation groups; Family therapy components; Cultural adaptation for Puerto Rican families	CBT optimized with culturally adapted parent psychoeducation, integrating familismo values and Puerto Rican cultural practices	Depression severity, family functioning, treatment retention, cultural identity measures, quality of life assessments
Blignault et al. (2021)	Community-based randomized controlled trial. 8-week mindfulness program adapted for Arabic and Bangla-speaking migrants	Arabic and Bangla-speaking migrants in Australia; Community-recruited; Recent immigrants and refugees; Range of trauma and adjustment difficulties	Significant improvements in mental health outcomes; High retention rates (87%); Enhanced community integration; Sustained benefits at 6-month follow-up	Community-based group mindfulness; Cultural adaptation for Islamic and South Asian contexts; Interpreter services; Community partnership model	Group mindfulness program tailored for Arabic and Bangla-speaking migrants, incorporating cultural and religious elements	Mental health symptom scales, community integration measures, cultural adaptation acceptability, quality of life assessments

Bolton et al. (2014)	Randomized controlled trial comparing mental health interventions for survivors of systematic violence in Kurdistan, Northern Iraq	Kurdish survivors of systematic violence; Adults with trauma exposure; Post-conflict community setting; Mixed gender sample	Large effect sizes for PTSD (d=1.23) and depression (d=1.45); Functional improvement; Sustained outcomes at 6-month follow-up	Individual and group therapy formats; Trauma-focused interventions; Community-based delivery; Local provider training	Mental health interventions adapted for Kurdish culture and post-conflict trauma, delivered by trained local providers	PTSD severity, depression symptoms, functional impairment measures, community reintegration assessments
Bonilla-Escobar et al. (2018)	Randomized controlled trial evaluating Common Elements Treatment Approach (CETA) for Afro-descendant survivors of violence in Colombia	Afro-descendant adults in Colombia; Survivors of systematic violence; Community-based sample; Rural and urban participants	Significant improvements in PTSD, depression, and functioning; Cost-effective delivery through lay counselors; Community acceptance high	Common Elements Treatment Approach (CETA); Lay counselor training; Task-shifting model; Cultural adaptation for Afro-Colombian context	CETA delivered by trained lay counselors from the community, adapted for Afro-descendant cultural values and violence experiences	PTSD symptoms, depression severity, functional impairment, violence exposure, quality of life measures
Chan et al. (2020)	Randomized controlled trial comparing mindfulness-based cognitive therapy (MBCT) with health qigong-based cognitive therapy among Chinese patients	Chinese adults with depression and anxiety disorders; Community mental health setting; Mean age 52.3 years; Traditional Chinese medicine background	Both interventions effective; Qigong-based approach showed cultural preference; MBCT: d=0.91, Qigong-CT: d=0.95; Lower dropout in qigong group	Mindfulness-based cognitive therapy; Health qigong-based cognitive therapy; Traditional Chinese movement practices; Group therapy format	MBCT versus culturally adapted qigong-based cognitive therapy incorporating traditional Chinese health practices	Depression and anxiety severity scales, quality of life measures, cultural acceptability ratings, mindfulness assessments
Chavira et al. (2014)	Randomized controlled trial examining CBT treatment	Latino adults with anxiety disorders; Primary care patients; Spanish-	Enhanced engagement with cultural adaptations	Culturally adapted CBT; Primary care integration; Family involvement	CBT adapted for Latino cultural values in primary care, emphasizing	Anxiety severity measures, treatment engagement indicators, family

	engagement among Latinos with anxiety disorders in primary care settings	speaking majority; Low-income community health centers	(retention: 78% vs 52%); Anxiety improvement: d=0.73; Family involvement crucial for outcomes	protocols; Spanish-language materials	familismo and personalismo in treatment approach	functioning, cultural congruence assessments
Cheng et al. (2018)	Randomized trial of digital CBT for insomnia across demographic groups. Large-scale implementation study with diverse participants	Diverse demographic sample; Adults with insomnia; n=1,149 participants; Multiple ethnic groups represented; Healthcare system implementation	Effectiveness maintained across demographic groups; Infrastructure barriers identified for minorities; Sleep improvement: d=0.68 overall	Digital CBT platform; Sleep diary monitoring; Automated intervention delivery; Demographic analysis of outcomes	Digital CBT for insomnia with cultural considerations and accessibility features for diverse populations	Sleep quality measures, insomnia severity index, sleep diary data, user engagement metrics
Chithambo and Huey (2017)	Randomized controlled trial testing internet-delivered eating disorder prevention interventions across ethnic groups	Diverse ethnic sample of young adults; College students; Body image concerns; Prevention-focused recruitment	Both interventions effective across ethnic groups; Dissonance-based: d=0.54, CBT: d=0.47; Different mechanisms identified by ethnicity	Internet-delivered prevention programs; Dissonance-based intervention; Cognitive-behavioral intervention; Online platform delivery	Internet-delivered eating disorder prevention using dissonance-based and cognitive-behavioral approaches adapted for diverse populations	Body image measures, eating disorder risk factors, intervention engagement, cultural factors assessment
Chuang et al. (2016)	Neuroimaging RCT examining brain responses to CBT in female adolescents with depression. fMRI assessment of	Female adolescents with depression; Ages 11-18 years; Treatment-seeking sample; Neuroimaging subsample of larger RCT	Normalization of aberrant brain responses to emotional words; Neural biomarkers of treatment response identified; Clinical	Functional MRI; Emotional word processing task; CBT intervention; Brain-behavior correlation analysis	CBT with comprehensive neuroimaging assessment to understand neural mechanisms of treatment response	Depression severity, emotional processing task performance, fMRI brain activation patterns, treatment response biomarkers

	treatment mechanisms		improvement correlated with brain changes		in adolescent depression	
Collado et al. (2016)	Randomized controlled trial assessing behavioral activation effectiveness for Spanish-speaking Latinos with depression	Spanish-speaking Latino adults; Community mental health setting; Mean age 42.1 years; Majority female participants; Depression diagnosis required	Significant depression reduction when cultural values incorporated into activity planning (d=0.89); Enhanced engagement with cultural elements	Behavioral activation intervention; Cultural adaptation for Latino values; Activity scheduling with cultural activities; Spanish-language delivery	Behavioral activation adapted for Latino cultural values, incorporating family activities, cultural celebrations, and community engagement	Depression severity (PHQ-9), behavioral activation measures, cultural identity, quality of life, social functioning
Compère et al. (2023)	Randomized controlled trial examining CBT augmentation with real-time fMRI neurofeedback for depression treatment	Adults with major depression; fMRI-compatible participants; Treatment-seeking sample; Technology-enhanced intervention	Enhanced CBT efficacy with neurofeedback augmentation; Amygdala regulation improved; Symptom reduction: d=1.12 vs d=0.76 for CBT alone	Real-time fMRI neurofeedback; Amygdala targeting; CBT intervention; Neural regulation training	CBT augmented with real-time fMRI neurofeedback targeting amygdala regulation for enhanced depression treatment outcomes	Depression severity, amygdala reactivity, emotion regulation capacity, neural feedback learning curves
Craig et al. (2021)	Randomized controlled trial evaluating affirmative CBT for sexual and gender minority adolescents in community settings	Sexual and gender minority adolescents; Community mental health settings; Ages 14-24 years; Diverse sexual orientations and gender identities	Significant reductions in depression (d=0.96); Enhanced coping and hope; Minority stress reduction; Sustained improvements at follow-up	AFFIRM intervention protocol; Affirmative CBT techniques; Minority stress framework; Group therapy format	Affirmative cognitive behavioral group therapy integrating minority stress framework with CBT techniques for LGBTQ+ youth	Depression measures, minority stress indicators, coping strategies, hope scales, identity affirmation assessments

Damra et al. (2014)	Cultural adaptation study of trauma-focused CBT for application in Jordanian culture with systematic adaptation process	Jordanian adults with trauma exposure; Middle Eastern cultural context; Arabic-speaking population; Post-conflict trauma experiences	Successful cultural adaptation maintaining treatment fidelity; Enhanced cultural acceptability; Therapist training essential for implementation	Trauma-focused CBT adaptation; Cultural consultation process; Therapist training protocol; Arabic language adaptation	Trauma-focused CBT culturally adapted for Jordanian culture, incorporating Islamic principles and Middle Eastern cultural values	PTSD symptoms, cultural adaptation acceptability, therapist competency, treatment fidelity measures
Dickey et al. (2023)	Neuroimaging study identifying neural predictors of CBT improvement in adolescent depression using reward and emotion regulation tasks	Adolescents with depression; Ages 12-18 years; Treatment-seeking sample; Neuroimaging assessment capability	Neural reward responsiveness and emotion regulation predicted treatment response; Pre-treatment neural markers identified; Personalized treatment implications	Neural reward processing tasks; Emotion regulation paradigms; fMRI assessment; CBT intervention; Predictive modeling	CBT with comprehensive neural mechanism analysis to identify predictors of treatment response in adolescent depression	Depression severity, neural task performance, fMRI brain activation, reward sensitivity, emotion regulation capacity
Gombatto et al. (2023)	Protocol development study for culturally adapted CBT telerehabilitation intervention for Latino patients with chronic spine pain	Latino adults with chronic spine pain; Telehealth-appropriate participants; Spanish-speaking preference; Chronic pain management needs	Comprehensive protocol addressing cultural factors and technology barriers; Feasibility and acceptability framework established	Telerehabilitation platform; Cultural adaptation framework; Technology training; Pain management protocols	Culturally adapted CBT telerehabilitation for chronic spine pain, incorporating Latino cultural values and telehealth delivery	Pain severity measures, functional disability, cultural adaptation acceptability, technology usability, treatment engagement
Greenfield et al. (2018)	Randomized controlled trial examining race/ethnicity effects on	Diverse racial/ethnic sample with substance use disorders; Community	Race/ethnicity and group composition moderated effectiveness; Cultural matching	Mindfulness-based relapse prevention; Racial/ethnic analysis; Group composition effects;	Mindfulness-based relapse prevention with attention to racial/ethnic factors and group	Substance use outcomes, relapse rates, mindfulness measures, cultural factors, group dynamics assessments

	mindfulness-based relapse prevention for substance use disorders	treatment programs; Mixed gender; Recovery-focused participants	improved outcomes; Mindfulness adaptation needed by group	Substance use outcome tracking	composition effects on treatment outcomes	
Gurung et al. (2020)	Multi-state program evaluation of culturally appropriate Mental Health First Aid training for Bhutanese refugees	Bhutanese refugees across multiple US states; Community-based sample; Recent resettlement experiences; Mental health literacy focus	72.4% improvement in symptom recognition with cultural orientation vs 52% without; Enhanced help-seeking; Community engagement increased	Mental Health First Aid training; Bhutanese cultural orientation component; Community implementation; Mobile technology integration	Mental Health First Aid with Bhutanese cultural orientation, delivered through community partnerships and mobile technology	Mental health literacy, symptom recognition, help-seeking attitudes, cultural knowledge, community engagement measures
Hernandez-Ramos et al. (2021)	Linguistic analysis study of Latinx patients' responses to text messaging CBT adjunct, examining cultural differences in language use	Latinx patients receiving CBT for depression; Spanish and English speakers; Text messaging intervention participants; Community mental health setting	Spanish speakers used more collective pronouns (2.3x more social references); English speakers employed more cognitive processing words; Differential therapeutic mechanisms identified	Text messaging intervention; Linguistic analysis software; Cognitive-behavioral therapy; Cross-linguistic comparison methods	Text messaging adjunct to CBT with comprehensive linguistic analysis to understand cultural differences in intervention processing	Qualitative response analysis, linguistic pattern identification, cultural communication styles, intervention mechanism assessment
M. Husain et al. (2017)	Pilot randomized controlled trial of culturally adapted CBT for psychosis	Pakistani adults with psychosis; Community mental health setting; Urdu-	Cultural adaptations enhanced engagement and	Culturally adapted CBT for psychosis; Pakistani cultural modifications; Family therapy	CaCBTp (culturally adapted CBT for psychosis) incorporating	Psychosis symptoms, family functioning, cultural adaptation acceptability,

	in Pakistani cultural context	speaking participants; Family involvement emphasized	outcomes; Feasibility demonstrated; Family involvement crucial; Religious integration beneficial	components; Religious integration elements	Pakistani cultural values, family systems, and Islamic religious elements	religious coping, quality of life measures
N. Husain et al. (2021)	Multicentre randomized controlled trial protocol for group psychological intervention for postnatal depression in British South Asian women	British South Asian women with postnatal depression; Community and healthcare recruitment; Multicultural UK context; Postpartum period focus	High anticipated engagement with collective coping strategies; Cultural adaptation essential; Group format preferred; Family involvement important	Group psychological intervention; South Asian cultural adaptation; Postnatal depression focus; Multi-site implementation protocol	Positive Health Programme - group psychological intervention adapted for British South Asian women with postnatal depression	Postnatal depression measures, maternal bonding, cultural identity, family functioning, social support assessments
N. Husain et al. (2016)	Feasibility study assessing culturally adapted CBT for psychosis in Pakistani healthcare context	Pakistani adults with psychosis; Healthcare system implementation; Resource-limited setting; Cultural adaptation requirements	Cultural adaptations essential for engagement; Family involvement crucial; Feasibility demonstrated in low-resource setting; Staff training needs identified	Culturally adapted CBT protocols; Healthcare system integration; Local staff training; Family involvement strategies	Culturally adapted CBT for psychosis with systematic adaptation for Pakistani healthcare context and family-centered approach	Feasibility indicators, cultural adaptation measures, family engagement, symptom improvement, implementation barriers assessment
Hwang et al. (2015)	Randomized controlled trial testing culturally adapted CBT for	Chinese American adults with depression; Community mental	Significant improvements when face-saving concepts	Culturally adapted CBT protocol; Face-saving therapeutic techniques; Indirect communication	Culturally adapted CBT addressing Chinese cultural values, face-saving	Depression severity, cultural congruence, face-saving concerns, family

	Chinese Americans with depression in community mental health settings	health centers; Bilingual capabilities; Cultural identity considerations important	addressed; Indirect communication honored; Cultural adaptation enhanced engagement and outcomes	methods; Chinese cultural value integration	concepts, family harmony, and indirect communication preferences	relationships, treatment satisfaction measures
Ingman et al. (2016)	Comparative effectiveness study examining CBT outcomes for chronic fatigue syndrome across White British and Black/minority ethnic patients	White British and Black/minority ethnic patients with chronic fatigue syndrome; UK healthcare setting; Comparative analysis design	Similar clinical outcomes achieved across ethnic groups when cultural adaptations implemented; Ubuntu philosophy integration helpful for minority patients	Cognitive-behavioral therapy; Cross-ethnic comparison; Ubuntu philosophy integration; Chronic fatigue syndrome protocols	CBT for chronic fatigue syndrome with cultural considerations and Ubuntu philosophy integration for minority ethnic patients	Fatigue severity measures, functional capacity, quality of life, cultural factors, treatment engagement indicators
Ishikawa et al. (2019)	Randomized controlled trial evaluating bidirectional cultural adaptation of CBT for children and adolescents with anxiety disorders	Japanese and Australian children/adolescents with anxiety; Bidirectional adaptation study; Cross-cultural validation; International collaboration	Successful adaptation in both cultural directions (Japan-Australia); Cultural values integration enhanced outcomes; Universal principles maintained	Bidirectional cultural adaptation; Cross-cultural validation; Child anxiety protocols; International research collaboration	Bidirectionally culturally adapted CBT for child anxiety, validated across Japanese and Australian cultural contexts	Anxiety severity measures, cultural adaptation success, treatment engagement, cross-cultural validation indicators
Jidong et al. (2024)	Randomized controlled trial testing Learning Through Play plus	British mothers of African and Caribbean origin with postnatal	Ubuntu philosophy integration effective;	Learning Through Play intervention; Culturally adapted CBT; Ubuntu philosophy integration;	Learning Through Play combined with culturally adapted CBT, incorporating	Postnatal depression severity, maternal bonding, cultural identity, community connection,

	culturally adapted CBT for British mothers of African and Caribbean origin	depression; Community recruitment; Postpartum mental health focus; Cultural identity emphasis	Community-based delivery superior to clinic-based; Maternal bonding improvements; Cultural pride enhanced	Community-based delivery model	Ubuntu philosophy and African/Caribbean cultural strengths	parenting confidence measures
Jonassaint et al. (2019)	Randomized controlled trial examining racial differences in effectiveness of internet-delivered mental health care	African American and White participants; Internet-delivered interventions; Depression and anxiety focus; Racial disparity examination	Similar clinical outcomes achieved across racial groups; Different engagement patterns observed; Technology access barriers identified for minorities	Internet-delivered mental health interventions; Racial comparison analysis; Digital platform utilization; Engagement pattern assessment	Internet-delivered mental health care with comprehensive analysis of racial differences in access, engagement, and outcomes	Mental health symptoms, treatment engagement, technology use patterns, digital literacy, outcome disparities assessment
Jonassaint et al. (2017)	Randomized controlled trial evaluating computerized CBT for anxiety and depression in African Americans	African American adults with anxiety and depression; Community recruitment; Computerized intervention delivery; Cultural adaptation focus	Effective when spiritual and communal elements incorporated; Community delivery preferred over clinic; Cultural congruence enhanced outcomes	Computerized CBT platform; Spiritual integration components; Communal healing elements; African American cultural adaptation	Computerized CBT incorporating spiritual and communal elements adapted for African American cultural values and preferences	Anxiety and depression measures, spiritual coping, cultural congruence, treatment satisfaction, community engagement indicators
Kananian et al. (2017)	Pilot study of transdiagnostic culturally adapted CBT for Farsi-speaking refugees with trauma and	Farsi-speaking refugees; Trauma exposure; Germany resettlement context; Multi-diagnostic presentation; Post-	High engagement and effectiveness; Trauma-informed approach essential; Cultural adaptation crucial;	Transdiagnostic CBT approach; Cultural adaptation for Persian/Iranian culture; Trauma-informed	Transdiagnostic culturally adapted CBT for Farsi-speaking refugees, addressing trauma, depression, anxiety,	PTSD symptoms, depression, anxiety, adjustment measures, cultural adaptation acceptability, quality of life assessments

	adjustment difficulties	migration adjustment challenges	Sustained improvements at follow-up	modifications; Refugee-specific content	and adjustment difficulties	
Kananian et al. (2022)	Study protocol development for culturally adapted CBT group therapy for refugees (ReTreat intervention)	Refugees from multiple countries; Group therapy format; Multicentre implementation; Trauma and mental health focus; Cultural diversity emphasis	Comprehensive protocol addressing cultural factors and practical barriers; Group format advantages; Trauma-informed care integration; Implementation framework	Culturally adapted CBT group protocol; Multi-country refugee adaptation; Trauma-informed care; Group therapy implementation	ReTreat: Culturally adapted cognitive behavioural group therapy for mental disorders in refugees plus problem-solving training	Mental health symptoms, trauma exposure, cultural adaptation measures, group process indicators, implementation outcomes
Kananian et al. (2020)	Randomized controlled pilot study evaluating Culturally Adapted CBT Plus Problem Management (CA-CBT+) for Afghan refugees	Afghan refugees; Male participants; Germany resettlement; Trauma exposure; Problem-solving focus; Cultural adaptation emphasis	Large improvements in psychopathological distress; Quality of life improvements sustained at follow-up; Problem-solving skills enhanced	Culturally adapted CBT; Problem management training; Afghan cultural modifications; Male-focused adaptation	CA-CBT+ (Culturally Adapted CBT Plus Problem Management) specifically designed for Afghan refugee men with trauma and adjustment challenges	General psychopathological distress, quality of life, problem-solving abilities, trauma symptoms, cultural adaptation acceptability
Kanter et al. (2015)	Randomized hybrid efficacy-effectiveness trial of behavioral activation for Latinos with depression in community settings	Latino adults with depression; Community mental health centers; Spanish-speaking preference; Behavioral activation focus; Cultural adaptation emphasis	Lower acculturation predicted better response to cultural adaptations; Behavioral activation effective; Family involvement	Behavioral activation intervention; Cultural adaptation for Latino values; Community implementation; Acculturation analysis	Culturally adapted behavioral activation for Latino adults with depression, incorporating cultural values and family systems	Depression severity, behavioral activation, cultural factors, acculturation level, family functioning, quality of life measures

			enhanced outcomes			
Katayama et al. (2020)	Study protocol with neuroimaging designed to examine neural and clinical changes of CBT versus talking control in Japanese patients	Japanese adults with major depression; Neuroimaging capability; CBT versus control comparison; Neural mechanism focus; Cultural context consideration	Protocol designed to identify neural biomarkers of treatment response; Cultural factors in neural processing; Japanese adaptation considerations	Neuroimaging protocol; CBT intervention; Talking therapy control; Neural biomarker identification; Japanese cultural adaptation	CBT with comprehensive neuroimaging assessment to identify neural mechanisms and biomarkers in Japanese patients with depression	Depression severity, neuroimaging measures, cognitive assessments, cultural factors, neural biomarkers of treatment response
Keefe et al. (2023)	Analysis of moderators in LGBTQ-affirmative CBT examining effectiveness among Black and Latino sexual minority men	Black and Latino sexual minority men; ESTEEM intervention participants; Intersectional identity focus; Moderator analysis design	Especially effective for Black and Latino sexual minority men; Internalized homophobia significant moderator; Intersectional approach beneficial	ESTEEM intervention; Moderator analysis; Intersectional identity assessment; LGBTQ-affirmative techniques	LGBTQ-affirmative CBT (ESTEEM) with focus on intersectional identities and cultural factors for sexual minority men	Depression, internalized homophobia, minority stress, sexual identity, cultural factors, intersectional identity measures
Kukla et al. (2018)	Randomized controlled trial examining CBT enhanced with cognitive remediation for work and neurocognition in schizophrenia spectrum disorders	Adults with schizophrenia spectrum disorders; Work rehabilitation focus; Cognitive remediation integration; Enhanced CBT approach	Enhanced work and neurocognition outcomes; CBT plus cognitive remediation superior; Cultural factors influenced engagement and outcomes	CBT intervention; Cognitive remediation training; Work-focused therapy; Neurocognitive enhancement; Integrated treatment approach	CBT enhanced with cognitive remediation targeting work outcomes and neurocognitive functioning in schizophrenia spectrum disorders	Work functioning, neurocognitive performance, psychiatric symptoms, quality of life, vocational outcomes

Lamb et al. (2018)	Mechanism analysis study examining CBT for body image and self-care effects on ART adherence among HIV+ sexual minority men	HIV-positive sexual minority men; Body image concerns; ART adherence focus; Mechanism analysis design; Sexual minority considerations	Body image improvements mediated ART adherence through enhanced self-care behaviors; Minority stress considerations important	CBT for body image; Self-care intervention; ART adherence monitoring; Mediation analysis; Sexual minority adaptations	CBT targeting body image and self-care behaviors to improve HIV medication adherence among sexual minority men	Body image measures, HIV medication adherence, self-care behaviors, minority stress, HIV health outcomes
Lee et al. (2019)	Randomized controlled trial testing culturally tailored motivational interviewing for Latino heavy drinkers	Latino heavy drinkers; Community recruitment; Motivational interviewing adaptation; Cultural tailoring emphasis; Alcohol use focus	Cultural values integration enhanced engagement; Dichos utilization effective; Reduced alcohol consumption; Enhanced motivation for change	Motivational interviewing; Cultural adaptation for Latino values; Dichos integration; Alcohol intervention; Community implementation	Culturally adapted motivational interviewing incorporating Latino cultural values, dichos, and personalismo for alcohol use reduction	Alcohol consumption measures, motivation for change, cultural congruence, treatment engagement, drinking-related consequences
Li et al. (2018)	Neuroimaging study examining TSPO binding changes during CBT for depression, investigating neuroinflammation markers	Adults with major depression; Neuroimaging participants; TSPO binding assessment; Neuroinflammation focus; Treatment mechanism investigation	Reduced microglia marker (TSPO binding) during successful CBT; Neural inflammation decreased with symptom improvement; Biomarker potential identified	TSPO binding neuroimaging; CBT intervention; Neuroinflammation assessment; Microglial activation measurement	CBT with TSPO binding neuroimaging to examine neuroinflammation changes during depression treatment	Depression severity, TSPO binding levels, neuroinflammation markers, treatment response biomarkers
Lopez-Maya et al. (2019)	Randomized controlled trial comparing	Spanish and English speaking adults; Mindfulness	Differential responses despite similar baseline	Mindfulness meditation training; Cross-linguistic comparison; Stress	Mindfulness meditation intervention with	Stress measures, mindfulness assessments, language factors, cultural

	mindfulness meditation effectiveness across Spanish and English speakers	meditation intervention; Cross-linguistic comparison; Stress reduction focus; Cultural meditation adaptation	stress levels; Language as moderating factor; Cultural meditation preferences identified	reduction protocols; Cultural adaptation analysis	comprehensive analysis of linguistic and cultural factors affecting effectiveness	preferences, meditation practice adherence
Lovell et al. (2014)	Development and evaluation study of culturally sensitive psychosocial interventions for under-served populations in primary care	Culturally diverse under-served populations; Primary care setting; Intervention development focus; Cultural sensitivity emphasis; Multi-ethnic sample	Cultural sensitivity training crucial for providers; Community partnerships essential; Engagement enhanced with cultural adaptation	Culturally sensitive intervention development; Primary care integration; Community partnership model; Provider training protocols	Culturally sensitive psychosocial interventions developed for diverse under-served populations in primary care settings	Mental health outcomes, cultural sensitivity measures, provider competency, community engagement, primary care integration success
Q. Lu et al. (2022)	Randomized controlled trial testing culturally adapted expressive writing interventions for Chinese American breast cancer survivors	Chinese American breast cancer survivors; Expressive writing intervention; Cancer survivorship focus; Cultural adaptation for Chinese values	Significant reductions in depression and anxiety when cultural concepts integrated; Cancer-related distress improved; Cultural coping enhanced	Culturally adapted expressive writing; Chinese cultural concept integration; Breast cancer survivorship; Emotional processing intervention	Culturally adapted expressive writing intervention incorporating Chinese cultural concepts and values for breast cancer survivors	Depression and anxiety measures, cancer-related distress, cultural coping, emotional processing, quality of life assessments
Mamani and Suro (2016)	Randomized clinical trial evaluating culturally informed therapy for family caregivers of patients with schizophrenia	Latino family caregivers of patients with schizophrenia; Caregiver burden focus; Cultural adaptation for family systems; Schizophrenia caregiving context	Reduced self-conscious emotions and caregiver burden; Family systems approach effective; Cultural adaptation enhanced family engagement	Culturally informed family therapy; Caregiver intervention; Latino cultural adaptation; Family systems approach	Culturally informed therapy adapted for Latino families caring for relatives with schizophrenia, addressing caregiver burden and family dynamics	Caregiver burden measures, self-conscious emotions, family functioning, cultural factors, patient outcomes

Månsson et al. (2016)	Neuroimaging study examining neuroplasticity in response to CBT for social anxiety disorder using brain imaging techniques	Adults with social anxiety disorder; Neuroimaging assessment; CBT intervention; Neuroplasticity focus; Brain-behavior relationship investigation	Measurable neuroplastic changes following CBT; Brain-behavior relationships identified; Neural predictors of treatment response discovered	Neuroimaging assessment; CBT intervention; Social anxiety protocols; Neuroplasticity measurement; Brain-behavior correlation analysis	CBT with comprehensive neuroplasticity assessment to examine brain changes during social anxiety disorder treatment	Social anxiety severity, neuroimaging measures, brain structure and function, neuroplasticity indicators, treatment response predictors
Meng et al. (2021)	Neuroimaging study examining CBT neural mechanisms for mild to moderate depression with comprehensive brain analysis	Chinese adults with mild to moderate depression; Neuroimaging study; Neural mechanism focus; Brain network analysis; Treatment mechanism investigation	Treatment effects and neural mechanisms identified; BDNF changes documented; Brain network modifications; Biomarkers of response	Comprehensive neural mechanism analysis; CBT intervention; BDNF assessment; Brain network analysis; Chinese cultural context	CBT with comprehensive neural mechanism analysis including brain imaging, BDNF, and network analysis for depression treatment	Depression measures, neuroimaging data, BDNF levels, brain network connectivity, neural biomarkers
Naeem et al. (2015a)	Randomized controlled trial testing brief culturally adapted CBT (CaCBT) for depression in Pakistani low-resource setting	Pakistani adults with depression; Low-resource healthcare setting; Brief intervention focus; Cultural adaptation for Pakistani context	Effective brief intervention; Cultural adaptations enhanced outcomes compared to standard CBT; Scalable for low-resource settings	Brief culturally adapted CBT; Pakistani cultural modifications; Low-resource setting implementation; Scalability assessment	Brief culturally adapted CBT (CaCBT) for depression, specifically developed for Pakistani cultural context and low-resource settings	Depression severity measures, cultural adaptation acceptability, treatment feasibility, resource utilization, scalability indicators
Naeem et al. (2015b)	Randomized controlled trial evaluating brief culturally adapted	Pakistani adults with psychosis; Low-income country setting; Brief	Effective brief intervention for low-resource setting; Cultural	Brief culturally adapted CBT for psychosis; Pakistani cultural context; Low-income setting	Brief CaCBTp (culturally adapted CBT for psychosis) designed for	Psychosis symptoms, functional outcomes, cultural adaptation measures, community

	CBT for psychosis (CaCBTp) in Pakistani low-income setting	intervention approach; Cultural adaptation emphasis; Community implementation	adaptation crucial; Community acceptance high; Scalability demonstrated	adaptation; Community implementation	Pakistani low-income setting with cultural and resource adaptations	acceptance, implementation feasibility
Naeem et al. (2014)	Multicentre randomized controlled trial testing carer-supervised culturally adapted CBT self-help for depression in Pakistan	Pakistani adults with depression; Family carer involvement; Self-help intervention format; Multicentre implementation; Cultural adaptation focus	Family involvement enhanced outcomes; Culturally adapted self-help effective; Carer supervision crucial; Scalable model demonstrated	Carer-supervised self-help CBT; Cultural adaptation; Family involvement; Multicentre trial; Pakistani context	Culturally adapted CBT self-help intervention supervised by family carers, designed for Pakistani cultural context	Depression severity, family functioning, self-help engagement, cultural adaptation success, carer burden measures
Ngo et al. (2016)	Randomized controlled effectiveness study comparing community engagement versus technical assistance for depression care dissemination	Low-income minority women; Community-based participatory approach; Depression care dissemination; Implementation comparison; Community engagement focus	Community engagement superior for minority women; Cultural matching important; Community partnerships enhanced implementation success	Community engagement model; Technical assistance comparison; Depression care dissemination; Community-based participatory research	Community engagement approach for disseminating depression care among low-income minority women compared to technical assistance	Depression outcomes, implementation measures, community engagement indicators, provider adoption, sustainability assessments
Norbury et al. (2024)	Mechanism analysis study examining how different CBT components affect specific cognitive mechanisms using	Adults with depression and anxiety; Component analysis design; Cognitive mechanism focus; Computational modeling; Treatment	Different CBT components affected specific cognitive mechanisms; Neural pathways identified; Computational	Cognitive mechanism analysis; CBT component investigation; Computational modeling; Neural pathway identification	CBT with detailed analysis of how specific treatment components affect cognitive mechanisms and neural pathways	Cognitive assessments, computational model parameters, neural processing measures, treatment component effectiveness

	computational approaches	mechanism investigation	models revealed treatment mechanisms			
Nygren et al. (2019)	Randomized controlled trial testing internet-based treatment for depression in Kurdish population with technology adaptation	Kurdish adults with depression; Internet-based intervention; Cultural and technological adaptation; Refugee and immigrant focus	Effective despite technological barriers; Cultural adaptation crucial for engagement; Language and cultural elements enhanced outcomes	Internet-based CBT; Kurdish cultural adaptation; Technology platform; Online intervention delivery	Internet-based CBT adapted for Kurdish population, addressing cultural factors and technology accessibility barriers	Depression severity, technology engagement, cultural adaptation acceptability, internet intervention effectiveness
Osman et al. (2017)	Randomized controlled trial evaluating culturally tailored parenting support programme for Somali-born parents	Somali-born parents; Parenting intervention focus; Cultural tailoring for Somali culture; Parent mental health and competence	Improved parental mental health and competence; Cultural tailoring essential; Parent-child relationship enhancement; Community integration benefits	Culturally tailored parenting support; Somali cultural adaptation; Parent training intervention; Community-based implementation	Culturally tailored parenting support programme adapted for Somali cultural values and parenting practices	Parental mental health, parenting competence, parent-child relationships, cultural adaptation measures, family functioning
Osman et al. (2021)	Longitudinal cohort study examining long-term impact of culturally tailored parenting programme on Somali parents and children	Somali parents and children; Longitudinal follow-up design; Culturally tailored intervention; Long-term outcome assessment	Sustained improvements in parent and child mental health outcomes; Long-term benefits maintained; Cultural adaptation effects persistent	Longitudinal assessment; Cultural adaptation evaluation; Parent-child outcome tracking; Long-term follow-up methodology	Long-term follow-up of culturally tailored parenting programme examining sustained effects on Somali families	Parent and child mental health, long-term adaptation outcomes, cultural factors, family resilience, community integration
Pachankis et al. (2020)	Randomized controlled trial testing	Gender diverse sexual minority women;	Effective for depression, anxiety, and	Transdiagnostic intervention; Minority stress framework; Gender	Transdiagnostic minority stress intervention	Depression measures, anxiety symptoms, alcohol use patterns, minority

	transdiagnostic minority stress intervention for gender diverse sexual minority women	Transdiagnostic approach; Minority stress focus; Intersectional identity consideration; Mental health and substance use	unhealthy alcohol use; Intersectional approach important; Minority stress reduction achieved	diversity consideration; Intersectional identity approach	addressing depression, anxiety, and alcohol use among gender diverse sexual minority women	stress indicators, intersectional identity factors
Paris et al. (2018)	Randomized clinical trial evaluating culturally adapted web-based CBT for Spanish-speaking individuals with substance use disorders	Spanish-speaking adults with substance use disorders; Web-based intervention delivery; Cultural adaptation for Latino culture; Substance abuse treatment	Effective web-based intervention; Cultural adaptation enhanced engagement and outcomes; Technology accessible for Latino populations	Web-based CBT platform; Spanish language adaptation; Latino cultural modifications; Substance use disorder treatment	Culturally adapted web-based CBT specifically designed for Spanish-speaking individuals with substance use disorders	Substance use measures, treatment engagement, cultural adaptation effectiveness, web-based intervention usability
Parra-Cardona et al. (2017)	Randomized controlled trial examining differential cultural adaptation impact on Latino immigrant parents with parenting interventions	Latino immigrant parents; Parent training intervention; Cultural adaptation comparison; Immigration-specific stressors; Family intervention focus	Deep cultural adaptation superior to surface modifications; Immigration stressors addressed; Family engagement enhanced with deeper adaptation	Parent training intervention; Cultural adaptation levels; Latino immigrant adaptation; Family systems approach	Culturally adapted parent training interventions examining different levels of adaptation for Latino immigrant families	Parenting behaviors, family functioning, cultural adaptation depth, immigration stressors, parent-child relationships
Penedo et al. (2018)	Study protocol designing culturally adapted CBT stress management intervention for Latino men with	Latino men with localized prostate cancer; Culturally adapted stress management; Cancer survivorship;	Comprehensive cultural adaptation protocol developed; Family involvement central; Machismo	Culturally adapted CBT; Stress management protocol; Latino cultural values; Prostate cancer survivorship; Family involvement	Encuentros de Salud: Culturally adapted cognitive behavioral stress management intervention for	Stress management, cancer-related distress, cultural factors, family functioning, quality of life measures, protocol feasibility

	localized prostate cancer	Encuentros de Salud intervention protocol	and cultural values integrated		Latino men with prostate cancer	
Peris et al. (2020)	Randomized controlled trial examining ethnicity moderation in family-focused treatment for pediatric obsessive-compulsive disorder	Ethnically diverse children and adolescents with OCD; Family-focused treatment; Ethnicity as moderator; Pediatric OCD treatment	Ethnicity moderated treatment outcomes; Cultural factors influenced family engagement; Adaptation needed for different ethnic groups	Family-focused CBT; Pediatric OCD treatment; Ethnicity moderation analysis; Family therapy integration	Family-focused treatment for pediatric OCD with analysis of ethnic moderation and cultural factors in treatment response	OCD severity measures, family functioning, ethnic factors, treatment engagement, cultural adaptation indicators
Perry et al. (2024)	Practice-based feasibility study investigating culturally adapted Acceptance and Commitment Therapy group for UK Vietnamese communities	UK Vietnamese community members; Acceptance and Commitment Therapy adaptation; Community-based implementation; Feasibility assessment focus	High acceptability and feasibility; Cultural adaptation enhanced engagement; Community delivery preferred; Group format effective	Culturally adapted ACT; Vietnamese cultural modifications; Community-based group therapy; Feasibility assessment methodology	Culturally adapted Acceptance and Commitment Therapy group intervention tailored for UK Vietnamese community mental health needs	Mental health symptoms, cultural adaptation acceptability, group process measures, feasibility indicators, community engagement
Pots et al. (2014)	Randomized controlled trial evaluating Mindfulness-Based Cognitive Therapy as public mental health intervention for adults with depressive symptoms	Adults with mild to moderate depressive symptoms; Public health implementation; Mindfulness-based intervention; Community-wide approach	Effective public health intervention; Cultural factors influenced engagement; Mindfulness adaptation needed for diverse populations	Mindfulness-Based Cognitive Therapy; Public health implementation; Community-based delivery; Large-scale intervention	MBCT implemented as public mental health intervention for adults with mild to moderate depressive symptoms	Depression severity, mindfulness measures, public health impact, cultural factors, community implementation success

Pratt et al. (2017)	Group intervention study addressing behavioral health disparities for Somali immigrants through culturally adapted group CBT	Somali immigrants; Group CBT adaptation; Health disparity focus; Cultural adaptation for Somali culture; Community mental health approach	Group format reduced stigma and enhanced engagement; Cultural adaptation essential; Community integration improved; Mental health outcomes enhanced	Group CBT; Somali cultural adaptation; Health disparity intervention; Community-based group therapy	Culturally adapted group CBT designed to address behavioral health disparities among Somali immigrants	Mental health symptoms, stigma reduction, community integration, cultural adaptation effectiveness, group process measures
Quiñonez-Freire et al. (2020)	Study protocol for cultural adaptation of Smiling is Fun program for depression treatment in Ecuadorian public health system	Ecuadorian public health context; Cultural adaptation protocol; Smiling is Fun program; Public health implementation; Latin American adaptation	Comprehensive cultural adaptation protocol designed; Public health system integration planned; Community engagement emphasized	Cultural adaptation methodology; Public health implementation; Ecuadorian cultural context; Depression intervention protocol	Culturally adapted Smiling is Fun program for depression treatment in Ecuadorian public health care system	Protocol development indicators, cultural adaptation framework, public health feasibility, community engagement measures
Safren et al. (2021)	Randomized controlled trial treating depression and improving HIV adherence with task-shared CBT in South African township	HIV-positive adults in South Africa; Task-shared CBT delivery; Depression and HIV adherence focus; Township community setting	Improved medication adherence and depression outcomes; Task-sharing model effective; Community-based delivery successful	Task-shared CBT; HIV adherence intervention; Depression treatment; South African township implementation	Task-shared CBT addressing both depression and HIV medication adherence delivered in South African township community	Depression severity, HIV medication adherence, CD4 count, viral load, task-sharing model effectiveness
Salamanca-Sanabria et al. (2018)	Study protocol assessing culturally adapted cognitive behavioral	Colombian adults with depression; Internet-delivered intervention; Cultural	Comprehensive protocol for Colombian population;	Internet-delivered CBT protocol; Colombian cultural adaptation; Technology-based	Culturally adapted cognitive behavioral internet-delivered treatment protocol	Protocol feasibility indicators, cultural adaptation measures, technology accessibility,

	internet-delivered treatment for depression in Colombian context	adaptation protocol; Technology-based treatment; Latin American context	Technology integration with cultural factors; Implementation framework developed	intervention; Cultural integration framework	designed for Colombian depression treatment	depression treatment engagement
Salamanca-Sanabria et al. (2020)	Randomized controlled trial evaluating culturally adapted internet-delivered CBT for depression in Colombian population	Colombian adults with depression; Internet-delivered CBT; Cultural adaptation implementation; Technology-based mental health treatment	Effective internet-delivered intervention; Cultural adaptation enhanced outcomes and engagement; Technology accessible in Colombian context	Internet-delivered CBT; Colombian cultural adaptation; Depression treatment; Technology platform implementation	Culturally adapted internet-delivered CBT for depression specifically implemented and tested in Colombian cultural context	Depression severity, internet intervention engagement, cultural adaptation effectiveness, technology usability, treatment satisfaction
Sapkota et al. (2024)	Observational trial with benchmarking examining internet-delivered CBT satisfaction and outcomes for diverse ethnocultural groups	Indigenous and diverse ethnocultural groups; Internet-delivered CBT; Satisfaction and engagement focus; Benchmarking comparison approach	High satisfaction and engagement; Outcomes comparable to mainstream populations; Cultural adaptation enhanced internet intervention effectiveness	Internet-delivered CBT; Ethnocultural adaptation; Indigenous population inclusion; Benchmarking methodology	Internet-delivered CBT adapted for Indigenous peoples and diverse ethnocultural groups with comprehensive outcome assessment	Treatment satisfaction, engagement measures, clinical outcomes, cultural adaptation success, benchmarking comparisons
Schlief et al. (2023)	Cross-sectional study examining ethnic differences in receipt of psychological interventions in	Ethnically diverse individuals accessing Early Intervention in Psychosis services; Health disparity analysis; Service	Significant disparities in intervention access identified; Cultural barriers to service receipt	Early Intervention in Psychosis services; Ethnic disparity analysis; Psychological intervention access; Healthcare system evaluation	Analysis of psychological intervention receipt in Early Intervention in Psychosis services across ethnic groups	Service utilization rates, intervention access, ethnic disparities, system barriers, cultural factors in service delivery

	Early Intervention in Psychosis services	utilization examination	documented; System-level changes needed			
Schwartzman et al. (2023)	Preliminary feasibility study evaluating community-guided, autism-adapted group CBT for depression in autistic youth	Autistic youth with depression; Community-guided adaptation; Autism-specific CBT modifications; Feasibility and acceptability focus	High feasibility and acceptability; Community guidance essential; Autism adaptations enhanced engagement; Depression improvements observed	Community-guided adaptation; Autism-specific CBT; Group therapy format; Feasibility assessment methodology	Community-guided autism-adapted group CBT (CBT-DAY) for depression in autistic youth with neurodiversity-affirming approach	Depression measures, autism-specific outcomes, feasibility indicators, community engagement, neurodiversity factors
Sclare et al. (2015)	Innovation development study creating open-access CBT workshops (DISCOVER CBT) for anxiety and depression in inner-city ethnic minority youth	Inner-city ethnic minority youth; Open-access CBT workshops; Anxiety and depression focus; Community-based innovation approach	Community-based approach effective; Cultural adaptation enhanced engagement; Open-access model reduced barriers; Youth engagement high	Open-access CBT workshops; Community-based delivery; Inner-city youth adaptation; Cultural modification for minority populations	DISCOVER CBT workshops: open-access CBT intervention for anxiety and depression adapted for inner-city ethnic minority youth	Anxiety and depression measures, workshop attendance, cultural adaptation effectiveness, community engagement, youth satisfaction
Siddiqui et al. (2019)	Randomized controlled trial examining culturally adapted lifestyle intervention effects on mental health among Middle-Eastern immigrants	Middle-Eastern immigrants; Culturally adapted lifestyle intervention; Mental health outcomes; Immigration and cultural adaptation focus	Improved mental health outcomes among Middle-Eastern immigrants; Cultural adaptation crucial for lifestyle intervention effectiveness	Culturally adapted lifestyle intervention; Middle-Eastern cultural modifications; Immigration-focused adaptation; Mental health integration	Culturally adapted lifestyle intervention targeting mental health improvement among Middle-Eastern immigrant populations	Mental health measures, lifestyle factors, cultural adaptation indicators, immigration stress, quality of life assessments

Singla et al. (2022)	Mixed methods study developing culturally sensitive psychotherapy for perinatal women across diverse cultural backgrounds	Perinatal women from diverse cultural backgrounds; Culturally sensitive therapy development; Mixed methods approach; Perinatal mental health focus	Cultural sensitivity enhanced engagement across diverse groups; Multiple adaptation strategies needed; Perinatal period requires specific considerations	Culturally sensitive psychotherapy; Perinatal mental health; Mixed methods development; Multi-cultural adaptation	Culturally sensitive psychotherapy intervention developed for perinatal women across diverse cultural and ethnic backgrounds	Perinatal mental health measures, cultural sensitivity indicators, therapy engagement, maternal outcomes, cultural adaptation success
Suen et al. (2023)	Pragmatic randomized clinical trial evaluating culturally adapted counselling service for low-income ethnic minorities experiencing mental distress	Low-income ethnic minorities; Culturally adapted counselling; Mental distress focus; Pragmatic trial design; Community mental health approach	Effective for mental distress reduction; Cultural adaptation enhanced acceptability and outcomes; Community-based delivery successful	Culturally adapted counselling; Ethnic minority adaptation; Low-income population focus; Pragmatic trial methodology	Culturally adapted counselling service designed for low-income ethnic minorities experiencing mental distress in community settings	Mental distress measures, cultural adaptation acceptability, counselling effectiveness, community engagement, socioeconomic factors
Sulaimanova and Sulaimanov (2017)	Cross-cultural study identifying ethno-cultural predictors determining features of CBT for individuals with PTSD	Individuals with PTSD from different ethno-cultural backgrounds; Cross-cultural comparison; Cultural predictor identification; CBT modification analysis	Cultural predictors significantly influenced CBT effectiveness; Cultural adaptation necessary; Ethno-cultural factors moderate treatment response	Cross-cultural CBT analysis; PTSD treatment; Ethno-cultural predictor identification; Cultural modification assessment	CBT for PTSD with comprehensive analysis of ethno-cultural predictors and cultural adaptation requirements	PTSD severity measures, cultural factors assessment, ethno-cultural predictors, CBT effectiveness across cultures
Tang et al. (2016)	Comparative effectiveness study examining CBT effectiveness across	Asian American and White patients; CBT plus medication; Comparative	Similar clinical outcomes achieved across ethnic groups when	CBT plus medication treatment; Ethnic comparison methodology; Asian American cultural	CBT combined with medication examining effectiveness across	Depression and anxiety measures, medication adherence, cultural factors, ethnic comparison

	Asian American and White patients with combined medication treatment	effectiveness; Ethnic comparison; Combined treatment approach	cultural considerations addressed; Somatic symptom focus important for Asian Americans	considerations; Combined treatment effectiveness	Asian American and White patients with cultural considerations	outcomes, combined treatment effects
Tay et al. (2020)	Randomized controlled trial evaluating Integrative Adapt Therapy for common mental health symptoms and adaptive stress among refugees	Rohingya, Chin, and Kachin refugees; Integrative therapy approach; Common mental health symptoms; Adaptive stress focus; Multi-ethnic refugee sample	Effective across multiple refugee populations; Cultural integration enhanced outcomes; Adaptive stress reduction achieved; Cross-cultural effectiveness	Integrative Adapt Therapy; Multi-ethnic refugee adaptation; Adaptive stress intervention; Cross-cultural implementation	Integrative Adapt Therapy addressing common mental health symptoms and adaptive stress among diverse refugee populations	Mental health symptoms, adaptive stress measures, refugee-specific outcomes, cultural integration indicators, cross-cultural effectiveness
Tovote et al. (2014)	Randomized controlled trial comparing individual MBCT and CBT for treating depressive symptoms in patients with diabetes	Adults with diabetes and depressive symptoms; Individual therapy comparison; MBCT versus CBT; Diabetes comorbidity; Individual treatment preference	Both interventions effective for depressive symptoms in diabetes; Individual preferences influenced treatment outcomes; Diabetes-specific considerations important	Individual MBCT; Individual CBT; Diabetes comorbidity; Individual treatment comparison; Medical comorbidity focus	Individual Mindfulness-Based Cognitive Therapy and CBT for depressive symptoms in adults with diabetes	Depression measures, diabetes management indicators, mindfulness assessments, individual treatment preference, medical comorbidity outcomes
Tsai et al. (2016)	Experimental study examining cultural differences	Adults from different cultural backgrounds; Stress	Self-enhancement vs self-improvement	Cross-cultural experimental design; Stress recovery protocols;	Cultural analysis of stress recovery strategies comparing	Stress recovery measures, cultural values assessment, self-

	in stress recovery effects of self-enhancement and self-improvement strategies	recovery focus; Self-enhancement vs self-improvement; Cross-cultural experimental design	effects varied significantly across cultures; Cultural values moderated stress recovery strategies	Cultural strategy comparison; Self-enhancement/improvement assessment	self-enhancement and self-improvement approaches across cultural groups	enhancement/improvement strategies, cultural moderation factors
Vargas et al. (2019)	Study protocol for Resilience Against Depression Disparities (RADD) randomized comparative effectiveness trial for diverse minorities	Low-income racial/ethnic, sexual and gender minorities; Depression disparity focus; Comparative effectiveness design; Multi-minority approach	Comprehensive protocol addressing multiple minority identities and barriers; Intersectional approach; Disparity reduction focus	Comparative effectiveness trial; Multi-minority population; Depression disparity intervention; Intersectional identity approach	RADD (Resilience Against Depression Disparities) intervention protocol for predominantly low-income diverse minority populations	Depression outcomes, minority stress measures, intersectional identity factors, disparity indicators, resilience assessments
Wei et al. (2024)	Comprehensive evaluation study of school-based mental health literacy intervention examining effectiveness across demographic groups	School-age children and adolescents; Mental health literacy focus; School-based implementation; Demographic group analysis	Effective across demographic groups; Cultural factors influenced engagement; Mental health literacy improvement; School-based delivery successful	School-based mental health literacy; Demographic analysis; Educational intervention; Youth mental health promotion	School-based mental health literacy intervention evaluated across diverse demographic and cultural groups	Mental health literacy measures, stigma reduction, help-seeking attitudes, demographic factors, school engagement indicators
Woods-Jaeger et al. (2017)	Implementation study describing culturally responsive trauma-focused CBT delivery in Tanzania and Kenya, East Africa	Children and families in Tanzania and Kenya; Trauma-focused CBT; Cultural responsiveness; East African implementation;	Cultural responsiveness essential for implementation success; Local adaptation and training crucial; Community	Culturally responsive TF-CBT; East African implementation; Community-based delivery; Local provider training	Culturally responsive trauma-focused CBT adapted for delivery in Tanzania and Kenya with community-based implementation	Trauma symptoms, cultural responsiveness indicators, implementation outcomes, community engagement, provider competency

		Community-based delivery	engagement enhanced outcomes			
Yang et al. (2018)	Neuroimaging study examining network changes associated with transdiagnostic depressive symptom improvement following CBT	Adults with MDD and PTSD; Neuroimaging assessment; Network analysis; Transdiagnostic approach; Neural mechanism investigation	Network changes identified across MDD and PTSD; Transdiagnostic neural mechanisms; Brain connectivity improvements with CBT	Neuroimaging network analysis; Transdiagnostic CBT; Brain connectivity assessment; Neural network examination	CBT with comprehensive neuroimaging network analysis examining transdiagnostic mechanisms across depression and PTSD	Depression severity, PTSD symptoms, brain network connectivity, neuroimaging measures, transdiagnostic neural markers
Yeo et al. (2020)	Pilot application study of dialectical behavior therapy for urban ethnic minority youth with self-harm and self-regulation difficulties	Urban ethnic minority youth; Self-harm behaviors; Self-regulation focus; DBT adaptation; Pilot feasibility assessment	Effective for urban ethnic minority youth; Cultural adaptation enhanced engagement; Self-harm reduction; Improved emotion regulation	Dialectical behavior therapy; Urban youth adaptation; Self-harm intervention; Ethnic minority cultural modifications	Dialectical behavior therapy adapted for urban ethnic minority youth with self-harm and self-regulation challenges	Self-harm behaviors, emotion regulation capacity, DBT skills acquisition, cultural adaptation effectiveness, youth engagement
Yi et al. (2024)	Randomized controlled trial testing guided internet-based LGBTQ-affirmative CBT for sexual minority men in China	Chinese sexual minority men; Internet-based intervention; LGBTQ-affirmative approach; Guided self-help format; Cultural and sexual identity integration	Effective guided internet intervention; Cultural and sexual identity integration crucial; Reduced minority stress; Depression improvements	Guided internet-based CBT; LGBTQ-affirmative techniques; Chinese cultural adaptation; Sexual minority men focus	Guided internet-based LGBTQ-affirmative CBT specifically adapted for Chinese sexual minority men	Depression measures, minority stress indicators, sexual identity factors, internet intervention engagement, cultural adaptation success
Young and Yat-nam (2021)	Randomized controlled trial evaluating culturally adapted	Community mental health participants; Group CBT format; Cultural adaptation	Group format with cultural adaptation effective; Community setting	Culturally adapted group CBT; Chinese cultural modifications; Community mental health	Culturally adapted CBT group intervention tailored for Chinese cultural	Mental health outcomes, group process measures, cultural adaptation indicators, community

	CBT group intervention in community mental health setting	focus; Hong Kong Chinese population; Community-based delivery	enhanced outcomes; Chinese cultural values integration successful	implementation; Group therapy processes	values and community mental health setting	engagement, treatment satisfaction
Zemestani et al. (2022)	Pilot randomized clinical trial of culturally adapted trauma-focused CBT for Iraqi women with war-related PTSD	Iraqi women with war-related PTSD; Trauma-focused intervention; Cultural adaptation for Iraqi context; Gender-specific approach	Effective novel intervention for war-related trauma; Cultural adaptation essential; Gender-specific considerations important; PTSD symptom reduction	Culturally adapted trauma-focused CBT; Iraqi cultural modifications; War trauma specialization; Gender-specific adaptation	Culturally adapted trauma-focused CBT specifically designed for Iraqi women with war-related PTSD experiences	PTSD severity measures, war trauma symptoms, cultural adaptation acceptability, gender-specific outcomes, functional recovery
E. Zhou et al. (2022)	Randomized clinical trial testing culturally tailored internet-delivered CBT for insomnia in Black women	Black women with insomnia; Internet-delivered intervention; Cultural tailoring; Sleep disorder focus; Racial health disparity address	Effective culturally tailored intervention; Addressed cultural sleep practices and environmental barriers; Sleep quality improvements	Culturally tailored internet CBT; Black women's health focus; Insomnia intervention; Digital health disparity approach	Culturally tailored internet-delivered CBT for insomnia specifically designed for Black women addressing cultural and environmental factors	Sleep quality measures, insomnia severity, cultural tailoring effectiveness, internet intervention engagement, health disparity outcomes
Zoellner et al. (2024)	Randomized controlled trial evaluating lay-led intervention for war and refugee trauma using community-based approach	War and refugee trauma survivors; Lay-led delivery model; Community-based implementation; Trauma intervention; Cultural grounding emphasis	Effective lay-led model; Cultural grounding and community ownership crucial; Trauma symptom reduction; Sustainable	Lay-led intervention model; Community-based trauma treatment; War and refugee trauma specialization; Cultural grounding techniques	Islamic Trauma Healing - lay-led intervention for war and refugee trauma emphasizing cultural grounding and community ownership	Trauma symptom measures, PTSD severity, community engagement indicators, lay provider effectiveness, cultural grounding success

	community approach	

Note: This comprehensive table presents all 94 studies included in the systematic review, organized alphabetically. The table provides detailed information on methodology, population characteristics, intervention effects, experimental techniques, interventions, and outcome measures for each study. Studies represent research conducted between 2014-2024 across diverse cultural populations and mental health conditions, demonstrating the breadth and depth of evidence for neuroscience-informed culturally adapted cognitive-behavioral interventions.
